# Repositioning antiviral phytoconstituents as broad-spectrum inhibitors of influenza A neuraminidase and human metapneumovirus fusion protein

**DOI:** 10.1371/journal.pone.0348517

**Published:** 2026-09-02

**Authors:** Nouh Mounadi, Hassan Nour, M’hammed El Kouali, Abdelouahid Samadi, Samir Chtita

**Affiliations:** 1 Laboratory of analytical and molecular chemistry, Faculty of Sciences Ben M’Sik, Hassan II University of Casablanca, Casablanca, Morocco; 2 Department of Chemistry, College of Science, United Arab Emirates University, Abu Dhabi, United Arab Emirates; Bai Jerbai Wadia Hospital for Children, INDIA

## Abstract

Viral respiratory infections still exist as a major global health concern. Influenza A viruses and human metapneumoviruses are among the main pathogens responsible for infections that can progress to severe acute respiratory forms. Despite the availability of certain therapeutic options, the need for effective treatment remains an issue. The exploration of innovative approaches based on medicinal plants, capable of simultaneously targeting several viruses, represents a promising strategic option for limiting the viral activity of these pathogens. In this work, an integrated computational workflow combining molecular docking, ADMET prediction, molecular dynamics (MD) simulations, and MM-PBSA binding free-energy analysis was applied to evaluate previously reported antiviral phytoconstituents for their broad-spectrum antiviral potential by simultaneously targeting influenza A virus neuraminidase (NA) (PDB ID: 2HT7) and the human metapneumovirus (HMPV) fusion protein (F) (PDB ID: 7SEJ). Molecular docking analyses identified 37 phytoconstituents with favorable multi-target binding affinities toward both NA and F proteins, exhibiting more favorable predicted binding energies than the reference compounds oseltamivir and ribavirin. In addition, ADMET prediction, including bioavailability assessment and analysis of pharmacokinetic and toxicological properties, identified several compounds with favorable profiles, notably compounds E10, M294, M329, C11, C5, and M274. MD simulations followed by MM-PBSA binding free-energy calculations further validated the stability and binding behavior of the highest-ranked protein–ligand complexes identified through the sequential screening workflow. Overall, compounds M294 and C11 emerged as the most promising broad-spectrum antiviral lead candidates against both influenza A NA and HMPV F protein. Nevertheless, these findings are based on computational predictions and require confirmation through in vitro antiviral assays followed by in vivo studies.

## 1. Introduction

Viral respiratory infections remain a major global public health concern. Among the most significant respiratory pathogens, Influenza A virus and human metapneumovirus (HMPV) are associated with a wide spectrum of respiratory diseases, ranging from mild symptoms to severe complications, particularly in vulnerable populations such as children and the elderly [1,2]. Influenza A viruses have historically caused major pandemics due to their high mutation rates and genetic reassortment capacity, which facilitate immune escape and reduce the effectiveness of existing antiviral therapies [3]. Although vaccines and antiviral drugs are available, therapeutic options remain limited, especially with the continuous emergence of new viral variants. These challenges highlight the urgent need to develop novel and effective broad-spectrum antiviral strategies. Neuraminidase (NA) is a key viral enzyme that has been extensively investigated as a therapeutic target for influenza A treatment, leading to the development of antiviral drugs such as oseltamivir and zanamivir. It plays a key role in the release of viral particles by cleaving the sialic acid residues present on the surface of host cells [4]. In addition, the fusion protein (F) plays an important role in HMPV virus entry by mediating fusion between the viral membrane and the host cell membrane [5]. Targeting NA and F addresses distinct, non-redundant processes, viral release and viral entry/fusion, respectively, thereby broadening the antiviral scope. By simultaneously targeting both proteins, our approach aims to achieve a multi-step inhibition of viral propagation, which is a promising strategy for broad-spectrum antiviral development.

Currently, antiviral treatments are often specific to a single virus and may have side effects. As for the HMPV virus, there is no specific treatment recommended to date. These limitations highlight the need to explore broad-spectrum antivirals with very low toxicity and good tolerance. Phytoconstituents represent an interesting natural source owing to their favorable safety profile and well-documented biological activities. Building on several of our previous studies, which led to the discovery of natural antivirals against SARS-CoV-2 and influenza A (H3N2) and have been complemented by numerous studies reporting their diverse biological activities, this project aims to evaluate their multi-target broad-spectrum antiviral potential.

Unlike most computational antiviral studies that focus on a single viral target, this study evaluates previously reported antiviral phytoconstituents against two distinct respiratory viruses by simultaneously targeting influenza A neuraminidase (NA) and the HMPV fusion protein (F), thereby providing an integrated framework for the identification of potential broad-spectrum antiviral candidates. The objective of this study was to identify the most promising candidates through an integrated computational workflow combining molecular docking, ADMET assessment, molecular dynamics simulations, and MM-PBSA binding free-energy calculations.

## 2. Materials and methods

### 2.1. Dataset sources

#### 2.1.1. Dataset background.

In our previous studies, we investigated several phytochemical datasets derived from Moroccan medicinal plants, cannabis derivatives, and eugenol-based compounds [6,12,13]. These datasets had previously demonstrated promising antiviral activities against influenza A (H3N2), particularly through hemagglutinin (HA) targeting, as well as against SARS-CoV-2 main protease (Mpro), in addition to other biological activities [6–13]. In the present work, these previously established datasets were reused for screening against influenza A neuraminidase (NA) and HMPV fusion (F) protein. The cannabis- and eugenol-derived datasets were directly retained based on their previously reported antiviral potential, whereas the Moroccan medicinal plant dataset underwent an additional docking-based screening step against both NA and F targets to prioritize the most promising compounds.

A total of 477 phytochemicals from these datasets were initially considered, of which 121 compounds were subsequently retained for further computational analyses, including molecular docking, ADMET and toxicity prediction, and molecular dynamics (MD) simulations. This study aims to promote a broad-spectrum multi-target strategy using natural resources as safe and well-tolerated solutions, while deepening our understanding of the molecular interactions involved in viral entry/fusion and viral release mechanisms. The structures and related information, including systematic names, identifiers (IDs), and phytochemical families, are provided in Supplementary S1 Table.

#### 2.1.2. Biological target.

The biological targets covered by this study include two proteins derived from viruses known to cause respiratory infections. The first is neuraminidase (NA), a key protein involved in viral release and spread (PDB identifier: 2HT7) [14,15]. NA is a well-validated antiviral target and has been extensively exploited in drug development (e.g., oseltamivir), complementing previous work on hemagglutinin (HA) to inhibit viral entry. The second is the fusion protein (F), designed to stabilize its pre-fusion conformation. This protein plays a key role in the entry of the HMPV virus by mediating the fusion between the viral membrane and the host cell membrane (PDB identifier: 7SEJ). Moreover, the F protein currently lacks approved therapeutics, making it a critical target for broad-spectrum antiviral discovery. Both crystallographic structures were extracted from the Protein Data Bank (PDB) due to their functional relevance and high-resolution data, making them suitable for molecular modeling and molecular dynamics simulations.

In addition, the selected PDB structures represent functionally relevant regions that are relatively conserved among viral strains. The catalytic site of influenza A neuraminidase is highly conserved across major influenza A subtypes, providing the structural basis for the broad activity of clinically approved neuraminidase inhibitors. Likewise, the HMPV fusion (F) protein contains conserved functional regions involved in viral membrane fusion across the principal HMPV genotypes. Therefore, PDB structures 2HT7 and 7SEJ constitute biologically relevant models for the identification of potential broad-spectrum antiviral compounds.

Selecting these two targets allows a multi-target approach that addresses distinct, non-redundant steps in the viral life cycle (entry/fusion vs. release), increasing the likelihood of identifying effective antiviral phytoconstituents. Although most broad-spectrum antiviral studies focus on multiple strains of the same virus or related viral families, this study targets two distinct respiratory viruses, influenza A and HMPV, representing different families and critical stages of the viral life cycle. This combination allows evaluation of natural compounds for potential broad-spectrum activity across unrelated viruses, which is relatively uncommon in the literature.

Furthermore, this study extends beyond conventional computational screening approaches by integrating molecular docking, ADMET and toxicity profiling, and molecular dynamics simulations within a unified workflow to systematically prioritize promising broad-spectrum antiviral candidates for subsequent experimental investigation.

### 2.2. Dataset preparation

#### 2.2.1. Ligands and protein preparation.

In order to ensure optimal results, good conformational stability of the system comprising ligands and proteins must be guaranteed. To this end, these structures were optimized. For the ligands, geometry optimization and energy minimization were performed in Avogadro using the MMFF94 force field and the conjugate gradient algorithm with a simple line-search technique. The convergence criterion was set to an energy threshold of 10^-4^ units with a maximum of 2500 optimization steps. Ligands and proteins were subsequently prepared using AutoDockTools [16]. The ligands were prepared by assigning Gasteiger charges and adding polar hydrogens. For the proteins, the structures of NA (PDB ID: 2HT7) and HMPV-F (ID: 7SEJ) were prepared by removing water molecules and crystallized co-ligands, followed by the addition of missing hydrogen atoms and the assignment of Kollman charges.

#### 2.2.2. Grid box setup.

**Fig 1:**
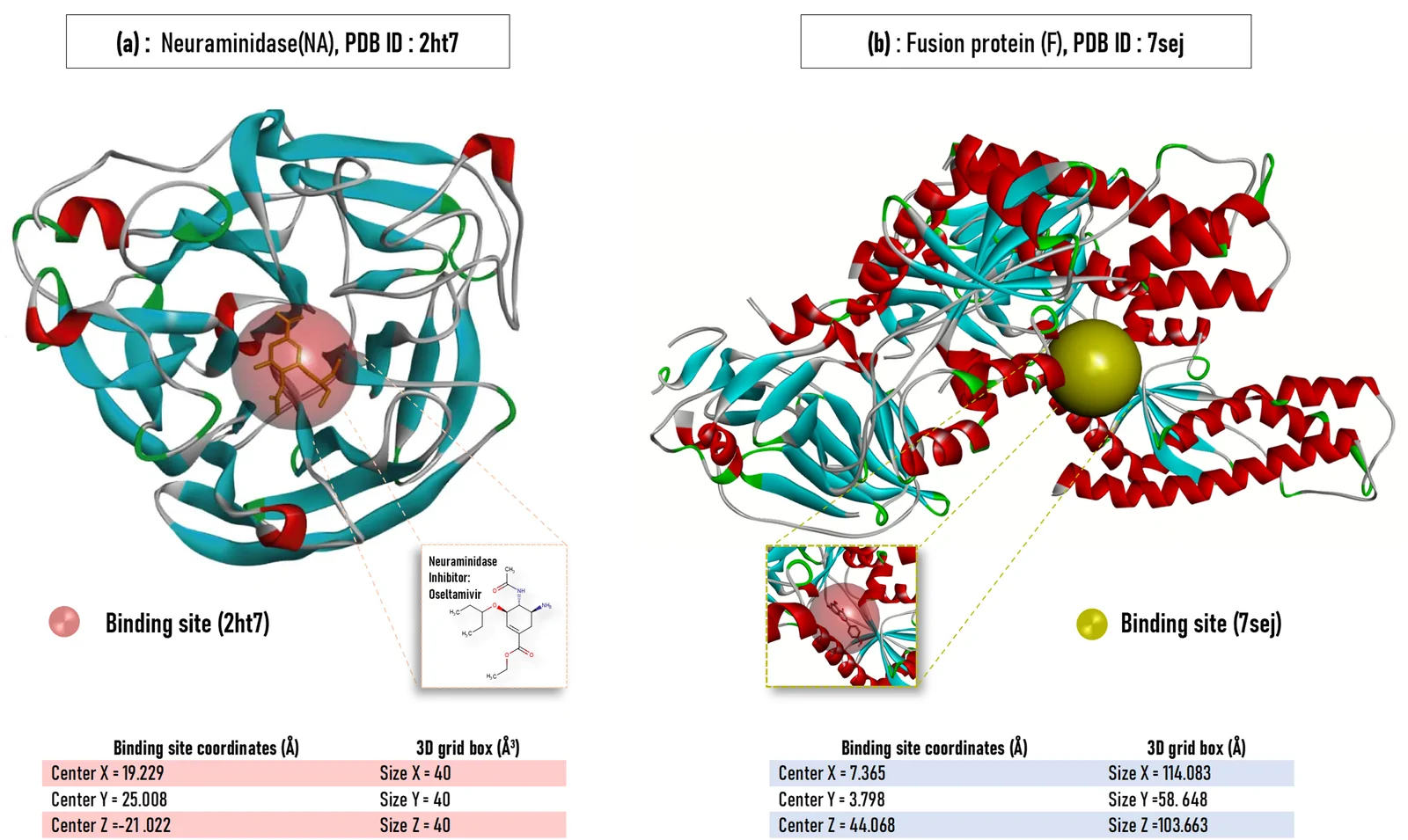
Three-dimensional visualization of the binding cavities selected for molecular docking, along with their corresponding coordinates: (a) 2HT7 neuraminidase receptor and (b) 7SEJ Fusion receptor.

In order to ensure accurate coverage of the active site of NA (PDB ID: 2HT7), the grid parameters representing the protein-ligand binding region were deduced using Discovery Studio, 2021 [17]. The center of the grid was determined based on the position of the co-crystallized ligand within the binding pocket, namely Oseltamivir, a well-known NA inhibitor and a drug widely used in the treatment of influenza A. For the F protein (PDB ID: 7SEJ), the grid center corresponding to the primary protease was determined using AutoDockTools [16]. The docking grids and their dimensions, illustrated in Fig 1, provide sufficient space to allow for ligand flexibility and exhaustive exploration of the entire binding cavity.

### 2.3. Molecular docking procedure of receptor–ligand complexes

Following the preparation of the database, molecular docking was performed using AutoDock Vina software (version 1.1.2), with the aim of evaluating ligand-protein affinity through the analysis of binding energies and corresponding interactions [16]. All phytoconstituents were docked onto the two viral targets studied (NA and F). The results were evaluated based on energy scores, binding mode, and finally the nature of the molecular interactions established with key and important residues of the active sites.

### 2.4. ADMET analysis

ADMET analysis (absorption, distribution, metabolism, excretion, and toxicity), including bioavailability and pharmacokinetic and toxicological properties, was performed using two online tools: SwissADME and ProTox-II (UN, 2013) [18,19]. This analysis was performed following the selection of molecules with a high affinity for both viral receptors studied. The first step in this process was to identify the molecules most suitable for oral administration. To this end, bioavailability was studied according to Lipinski’s and Veber’s rules [20,21]. Table 1 describes the various criteria and parameters on which we based our decision at this stage. Next, all of the selected molecules that showed favorable bioavailability compared to the reference drugs were analyzed in terms of their pharmacokinetic and toxicological profiles, with the aim of proposing molecules that were more suitable for safe medicinal use. Finally, the best selection was chosen for further study using molecular dynamics simulations.

**Table 1:** Crietria of Lipinski’s and Veber’s rules.

Rule	Parameter	Criterion
Lipinski’s Rule of Five	Hydrogen bond donors (HBD)	≤ 5
	Hydrogen bond acceptors (HBA)	≤ 10
	Molecular weight (MW)	≤ 500 Daltons
	Log P (partition coefficient)	≤ 5
Veber’s Rule	Rotatable bonds (RB)	≤ 10
	Topological polar surface area (TPSA)	≤ 140 Å^2^

### 2.5. Molecular dynamics simulations

Molecular dynamics simulations were conducted using Maestro, part of the Schrödinger software suite, coupled with Desmond, in order to examine the stability of complexes, ligand-protein behavior, and their interactions over time [22]. Following the selection of the most favorable candidates through various molecular docking and ADMET analyses, these compounds were prepared in complex with the viral targets studied. System preparation consisted of the removal of water molecules and structural optimization, followed by solvation in an orthorhombic box with a 10 Å buffer around the complex, using the simple point charge (SPC) water model, as well as neutralization by adding Na^+^ and Cl^−^ ions at a concentration of 0.15 M [23,24]. After system relaxation, the simulations were subjected to a 1 ns equilibration phase under the NVT ensemble, followed by a 100 ns production run under the NPT ensemble, at a temperature set at 300 K and a pressure of 1 atm [25]. Trajectories were analyzed to extract key structural and dynamic parameters, including root-mean-square deviation (RMSD), root-mean-square fluctuation (RMSF), and ligand–protein contact diagrams.

In addition, binding free-energy calculations were performed using the Molecular Mechanics Poisson–Boltzmann Surface Area (MM-PBSA) approach to further evaluate the binding affinity of the selected protein–ligand complexes. The calculations were carried out using the g_mmpbsa tool based on the equilibrated MD trajectories [26]. The resulting binding free-energy values (ΔG_bind) were used as a complementary energetic measure of protein–ligand binding, where more negative values indicate more favorable interactions.

## 3. Results and discussion

### 3.1. Molecular docking simulation

A molecular docking study was conducted on the entire ligand library, specifically targeting the active sites of NA (PDB ID: 2HT7) and the fusion protein (PDB ID: 7SEJ), representing multi-targeted influenza A and HMPV viruses, respectively. The objective of this study was to evaluate the potential of these phytoconstituents to exhibit broad-spectrum antiviral activity by examining their binding capacity and affinity toward the selected biological targets studied. In this context, two drugs were carefully selected as reference compounds for comparison with the candidate ligands: oseltamivir (NA) and ribavirin (F). The docking results revealed that, among the 121 phytoconstituents tested, several ligands exhibited more favorable predicted binding energies than the two reference drugs (oseltamivir: −6.2 kcal/mol, ribavirin: −5.8 kcal/mol) (S2 Table). Among them, 37 ligands showed a particularly high affinity toward both NA and F receptors. The selection criteria applied were: BE(NA) ≤ −7.4 kcal/mol; BE(F) ≤ −7.0 kcal/mol (Table 2). These results suggest that several phytochemical compounds may interact favorably with the binding sites of NA and F, supporting their prioritization as candidate inhibitors for further investigation.

**Table 2:** Molecular docking binding energies (BE) of the top selected ligands targeting both NA (PDB ID: 2HT7) and HMPV-F proteins (PDB ID: 7SEJ) compared with reference compounds.

Famille/ Origine	Ligand	BE (kcal/mol) (PDB ID: 2HT7)	BE (kcal/mol) (PDB ID: 7SEJ)	Criteria Met
*Cannabis* *(Cannabinoïdes)*	C5	−7.7	−7.4	Yes
	C7	−7.6	−7.4	Yes
	C11	−8.0	−7.3	Yes
*Eugenol derivatives*	E10	−8.2	−8.5	Yes
	E18	−7.9	−7.1	Yes
	E19	−8.0	−7.3	Yes
	E51	−9.5	−9.2	Yes
	E57	−7.4	−7.1	Yes
	E58	−8.4	−7.2	Yes
	E59	−8.3	−7.7	Yes
*Marocain Medicinal Plants*	M110	−10.4	−9.1	Yes
	M17	−10.0	−8.2	Yes
	M293	−10.0	−9.1	Yes
	M319	−9.1	−8.0	Yes
	M322	−9.1	−8.1	Yes
	M292	−9.0	−8.1	Yes
	M324	−9.0	−8.7	Yes
	M378	−9.0	−8.4	Yes
	M269	−8.9	−8.5	Yes
	M285	−8.9	−8.1	Yes
	M16	−8.7	−8.0	Yes
	M320	−8.7	−8.7	Yes
	M321	−8.7	−8.5	Yes
	M286	−8.6	−8.2	Yes
	M274	−8.5	−8.2	Yes
	M109	−8.4	−7.3	Yes
	M111	−8.4	−8.1	Yes
	M270	−8.4	−9.1	Yes
	M267	−8.1	−8.6	Yes
	M268	−8.1	−8.6	Yes
	M295	−8.1	−8.3	Yes
	M148	−8.1	−7.0	Yes
	M22	−8.0	−7.3	Yes
	M276	−8.0	−7.2	Yes
	M329	−8.0	−7.3	Yes
	M294	−7.4	−8.0	Yes
	M81	−7.4	−7.4	Yes
References		**−6.2** ^**a**^	**−5.8** ^**b**^	

**BE**: binding energies, **Reference**^**a:**^ Oseltamivir, **Reference**^**b:**^ Ribavirin

**Fig 2:**
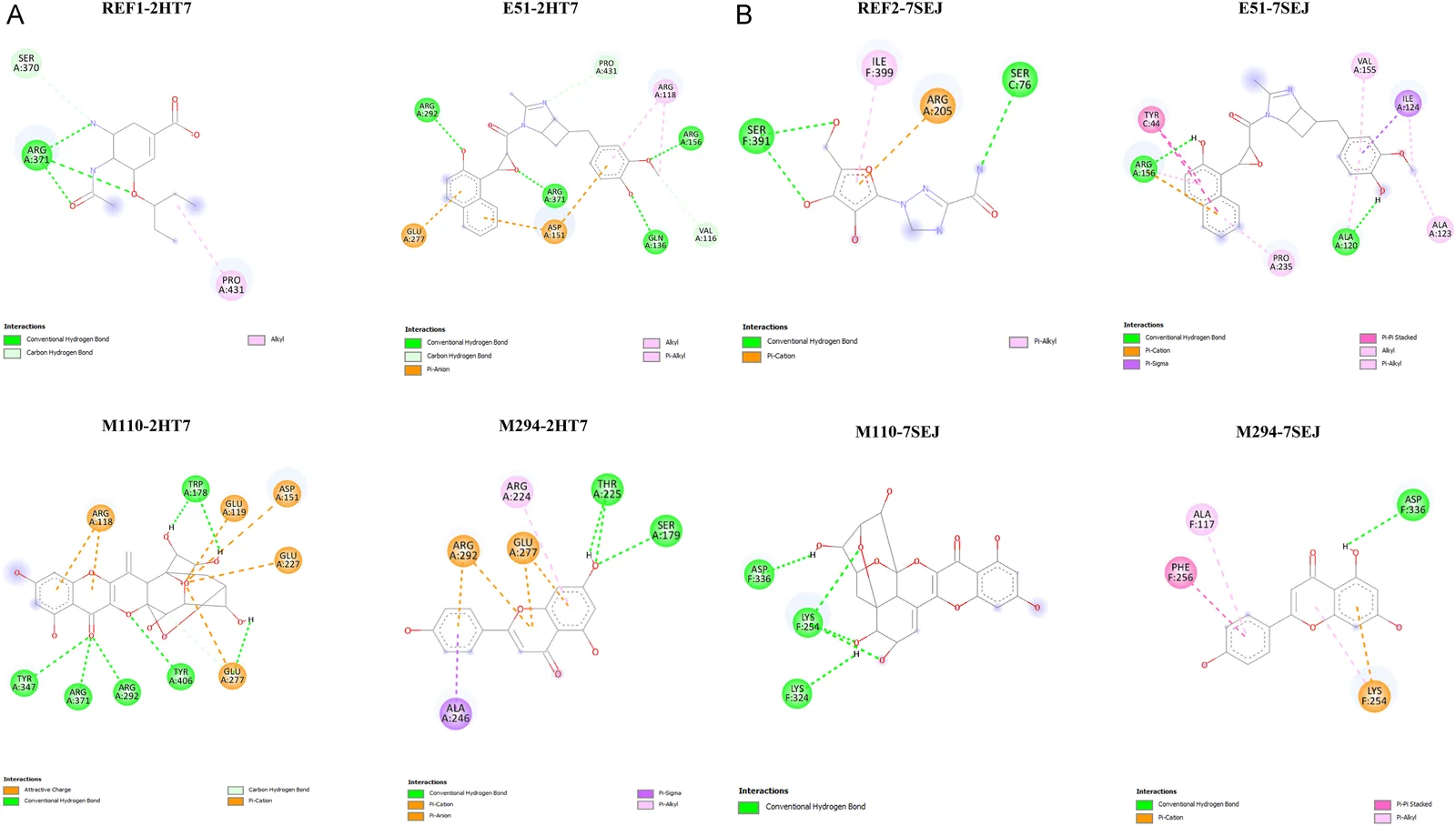
Representative 2D interaction diagrams of the best multitarget candidates targeting both NA (a) (PDB ID: 2HT7) and (b) HMPV-F proteins (PDB ID: 7SEJ), compared with reference compounds (REF.

Representative 2D interaction diagrams of the best multitarget candidates and reference drugs are presented in Figs 2(a–b). The selected compounds exhibited stable binding within the active pockets of both NA and F proteins through multiple conventional hydrogen bonds, π-interactions, hydrophobic contacts, and electrostatic interactions with key amino acid residues.

In the NA active site, residues such as Arg292, Arg371, Glu277, and Asp151 were among the key residues involved in ligand stabilization through hydrogen bonding and electrostatic interactions. In the F protein pocket, Lys254, Arg156, and Asp336 were the residues most frequently involved in ligand binding. Compared with the reference drugs, compounds E51 and M110 displayed more extensive interaction networks and lower binding energies, supporting their favorable multitarget binding profiles and potential antiviral activity.

### 3.2. ADMET predictions

#### 3.2.1. Evaluation of Drug-likeness properties.

Based on the results of molecular docking, 37 ligands were selected for evaluation of drug-likeness properties. The objective of this step is to select the compounds best suited for drug development, compared to the drug references studied, and presenting optimal oral bioavailability. To do this, Lipinski’s and Veber’s 5 rules were used as mandatory selection criteria. S3 Table presents the predicted data from this study. According to the results obtained, only 13 ligands, summarized in Table 3, were favored and selected because they meet all the considered criteria without any violations, and demonstrated a predicted oral bioavailability superior to that of both reference drugs. This promising selection was taken for further analysis.

**Table 3:** Physicochemical properties of the top selected ligands meeting Lipinski’s and Veber’s rules in comparison with reference compounds.

Ligand	Formula	Lipinski and Veber parameters						Number of violations		Criteria Met
		Mw	LogP	RB	HBA	HBD	PSA	Lipinski	Veber	
C5	C19H26O2	286.41	4.68	2	2	1	29.46	0	0	Yes
C11	C22H30O4	358.47	4.94	5	4	2	66.76	0	0	Yes
E10	C22H3O7	379.26	0.78	5	7	3	80.14	0	0	Yes
E18	C17H2NO2	252.20	2.40	4	2	1	30.49	0	0	Yes
E19	C18H2NO3	280.21	2.30	5	3	1	39.72	0	0	Yes
E51	C27H2N2O5	434.32	1.53	6	6	2	82.53	0	0	Yes
E57	C19ClNO2	309.66	3.42	5	3	0	31.35	0	0	Yes
E59	C27HClN2O4	452.76	2.83	9	5	1	59.00	0	0	Yes
M285	C27H42O7	478.62	1.76	2	7	5	127.59	0	0	Yes
M274	C24H32O6	416.51	1.93	1	6	3	104.06	0	0	Yes
M22	C15H10O6	286.24	1.73	1	6	4	111.13	0	0	Yes
M329	C17H14O7	330.29	2.11	3	7	3	109.36	0	0	Yes
M294	C15H10O5	270.24	2.11	1	5	3	90.90	0	0	Yes
Ref.^1^	C16H28N2O4	312.40	1.38	9	5	2	90.65	0	0	N/A
Ref.^2^	C8H12N4O5	244.20	−2.14	3	7	4	143.72	0	1	N/A
			≤ 5	≤10	≤ 10	≤ 5	≤ 140	≤2	≤1	

**Reference**^**1:**^ Oseltamivir, **Reference**^**2:**^ Ribavirin.

#### 3.2.2. Evaluation of pharmacokinetics and toxicity properties.

Next, the selection obtained from the bioavailability assessment was evaluated in terms of pharmacokinetic properties and toxicity in order to highlight the therapeutic potential of the compounds and their suitability as leading candidates for broad-spectrum antiviral applications. This study includes the evaluation of several parameters, namely water solubility (WS), Pain alerts, gastrointestinal absorption (G.I.A), blood-brain barrier permeability (BBB), inhibition of cytochrome P-450 enzymes (CYP2D6, CYP3A4, CYP1A2, CYP2C19, and CYP2C9), and skin permeability (S.P). As for toxicity, the prediction parameters considered include LD50, neurotoxicity, cytotoxicity, carcinogenicity, mutagenicity, and hepatotoxicity.

According to the results summarized in Table 4, the compounds were classified according to their water solubility as follows: very soluble (V.S.) for Ref.1 and Ref.2; soluble (S) for E10, M285, and M274; moderately soluble (M.S.) for C5, E18, E19, E51, E57, M22, M329, and M294; and poorly soluble (P.S.) for C11 and E59. Among the studied compounds, C11 and E59 exhibited the lowest solubility, whereas M22 showed moderate solubility. Overall, most compounds were classified as soluble or very soluble. In addition, all investigated compounds were predicted to be free of PAINS alerts, except compound M22. Most compounds were also predicted to be unable to permeate the blood–brain barrier (BBB), with the exception of C5 and E57. Despite this, they demonstrated high gastrointestinal absorption and acceptable skin permeability, with permeability values ranging from −10.0 to −1.0 cm/s. Assessing the inhibitory power of cytochrome P450 enzymes is essential when selecting drug candidates. This assessment provides information on whether these candidates are likely to inhibit the enzymes responsible for breaking down active substances. Such inhibition can lead to an accumulation of these active substances and/or their metabolites in the human body, with extremely toxic consequences.

**Table 4:** Pharmacokinetic properties (ADME) of the top selected ligands compared with reference compounds.

Ligand	Pharmacokinetic parameters									
	WS	Pain alert	G.I. A	BBB	CYP2D6	CYP3A4	CYP1A2	CYP2C19	CYP2C9	S.P (cm/s)
C5	M.S	0	High	Yes	Yes	No	No	Yes	Yes	-3.87
C11	P.S	0	High	No	No	Yes	No	No	Yes	-3.48
E10	S	0	High	No	No	No	No	No	Yes	-6.91
E18	M.S	0	High	No	No	No	No	No	Yes	-4.77
E19	M.S	0	High	No	Yes	No	No	Yes	Yes	-4.96
E51	M.S	0	High	No	No	No	No	No	Yes	-6.14
E57	M.S	0	High	Yes	No	No	Yes	Yes	Yes	-4.36
E59	P.S	0	High	No	No	No	No	Yes	Yes	-5.02
M285	S	0	High	No	No	No	No	No	No	-8.73
M274	S	0	High	No	No	No	No	No	No	-8.07
M22	M.S	1	High	No	Yes	Yes	Yes	No	No	-6.25
M329	M.S	0	High	No	Yes	Yes	Yes	No	Yes	-6.14
M294	M.S	0	High	No	Yes	Yes	Yes	No	No	-5.80
Ref.1	V.S	0	High	No	No	No	No	No	No	-7.42
Ref.2	V.S	0	Low	No	No	No	No	No	No	-9.10

**M.S**: Moderately soluble, **P.S**: Poorly soluble, **WS**: Water Solubility, **BBB:** Blood–Brain Barrier, **G.I.A**: Gastrointestinal Absorption, **S.P**: Skin Permeation, **Reference**^**1:**^ Oseltamivir, **Reference**^**2:**^ Ribavirin.

According to the results obtained, compounds M285, M274, E10, E51, and E18 are the most favorable in terms of non-inhibition of cytochrome P450 enzymes, with the exception of compounds E10, E51, and E18, which show a single inhibition related to the CYP2C9 enzyme.

Compound C11 was predicted to inhibit CYP2C9 and CYP3A4 enzymes, while compound E59 was predicted to inhibit CYP3A4 and CYP2C19 enzymes, respectively.

Compounds C5, E57, and E19 reported inhibition violations involving three different enzymes. Indeed, these compounds were predicted to be common inhibitors of CYP2C19 and CYP2C9. In addition, compound C5 is estimated to be inhibit CYP2D6, while compound E57 appears to be an inhibitor of CYP1A2. Finally, compound E19 does not inhibit the CYP2D6 enzyme. Compounds M329, M294, and M22 were predicted to be inhibitors of the CYP2D6, CYP3A, and CYP1A2 enzymes, with additional inhibition of CYP2C9 for compound M329.

The evaluation of toxicity parameters is a crucial step in an ADMET study, as it allows for the prediction of safe use of candidates in drug development. In this regard, low and acceptable toxicity is highly desirable. At this stage, the information obtained from these parameters makes it possible to reduce the number of selected compounds and differentiate them from the two reference drugs, while taking into account the other criteria evaluated.

According to the results obtained (Table 5), compounds E10, M329, and M294 showed no violation of toxicity parameters. Compounds C11, and C5 meet all predicted criteria, except for LD50 (less than 1000 mg/kg). Compound M274 showed a single violation related to carcinogenicity, while compounds E19 and E59 were predicted to be neurotoxic and hepatotoxic. Compounds E51 and E57 showed low LD50; in addition, compound E51 appears to be neurotoxic and cytotoxic, while compound E57 is neurotoxic and hepatotoxic.

**Table 5:** Toxicity prediction of the top selected ligands compared with reference compounds.

Ligand	Toxicity												
	LD50 (mg/kg)	Neuro-toxicity	Neuro-toxicity Prob.	Cyto-toxicity	Cyto-toxicity Prob.	Carcino-genicity	Carcino-genicity Prob.	Muta- genicity	Muta-genicity Prob.	Hepato- toxicity	Hepato-toxicity Prob.	Classe	Yes/No
C5	482	Inactive	0.58	Inactive	0.83	Inactive	0.84	Inactive	0.74	Inactive	0.85	4	Yes
C11	500	Inactive	0.76	Inactive	0.79	Inactive	0.70	Inactive	0.68	Inactive	0.82	4	Yes
E10	5000	Inactive	0.83	Inactive	0.78	Inactive	0.72	Inactive	0.73	Inactive	0.76	5	Yes
E18	1600	Active	0.58	Inactive	0.77	Active	0.54	Active	0.55	Active	0.56	4	No
E19	1190	Active	0.50	Inactive	0.78	Inactive	0.53	Inactive	0.61	Active	0.55	4	No
E51	709	Active	0.62	Active	0.62	Inactive	0.64	Inactive	0.51	Inactive	0.61	4	No
E57	500	Active	0.66	Inactive	0.70	Inactive	0.50	Inactive	0.57	Active	0.55	4	No
E59	1190	Active	0.74	Inactive	0.69	Inactive	0.63	Inactive	0.72	Active	0.57	4	No
M285	34	Inactive	0.91	Inactive	0.79	Active	0.60	Inactive	0.76	Inactive	0.82	2	No
M274	2450	Inactive	0.91	Inactive	0.77	Active	0.54	Inactive	0.90	Inactive	0.84	5	Yes
M22	3919	Inactive	0.89	Inactive	0.99	Active	0.68	Active	0.51	Inactive	0.69	5	No
M329	3919	Inactive	0.87	Inactive	0.90	Inactive	0.69	Inactive	0.91	Inactive	0.71	5	Yes
M294	2500	Inactive	0.86	Inactive	0.87	Inactive	0.62	Inactive	0.57	Inactive	0.68	5	Yes
Ref.1	260	Inactive	0.58	Inactive	0.68	Inactive	0.65	Inactive	0.69	Inactive	0.72	3	N/A
Ref.2	2700	Active	0.63	Inactive	0.72	Active	0.56	Inactive	0.85	Inactive	0.52	5	N/A

**prob.:** Probability, **Reference**^**1**^**:** Oseltamivir, **Reference**^**2**^**:** Ribavirin

In this table, “**Active**” denotes a positive toxicity prediction for the corresponding endpoint, and “**inactive**” denotes a negative prediction. Prediction probabilities >0.70 are considered meaningful.

Finally, compounds M285, E18, and M22 were excluded from selection for further analysis due to unfavorable toxicological profiles. Compound M285 has an extremely low LD50 (34 mg/kg) and predicted carcinogenicity, while compound M22 is predicted to be both mutagenic and carcinogenic. In addition, compound E18 has several violations, including cytotoxicity, carcinogenicity, mutagenicity, and hepatotoxicity.

In general, ADMET analysis led to a compromise in the selection of the most favorable candidates in terms of their pharmacokinetic and toxicological profiles. Compared to the two reference drugs studied, Oseltamivir, which has an extremely low LD50, and Ribavirin, which appears to be both neurotoxic and carcinogenic, with low gastrointestinal absorption, compounds E10, M294, and M329 stand out with impeccable toxicological properties, no violations observed, and an acceptable ADME profile, placing them in first position as the best candidates. Furthermore, Compounds C11 and C5 present a single toxicity concern (LD50 < 1000 mg/kg), while M274 shows only a predicted carcinogenicity flag. All other ADMET endpoints are favorable. It is important to emphasize that these compounds are exploratory in silico leads intended for further investigation rather than confirmed drug candidates. The predicted ADMET and toxicity profiles should be interpreted with caution, as they are derived from computational models that provide preliminary estimates rather than definitive pharmacokinetic or toxicological outcomes. Although these predictions are valuable for early-stage compound prioritization, they cannot replace experimental pharmacokinetic and toxicological evaluations. Their overall ADMET profiles are more favorable than several discarded compounds and, in some cases, more favorable than at least one reference drug, supporting their inclusion as candidates for further study. On the other hand, compounds showing multiple toxicity violations and interactions with CYP450 enzymes, such as compounds M285, E18, and M22, E51, E57, E19, and E59, are discarded.

### 3.3. Molecular dynamics simulations

#### 3.3.1. RMSD profiles.

**Fig 3:**
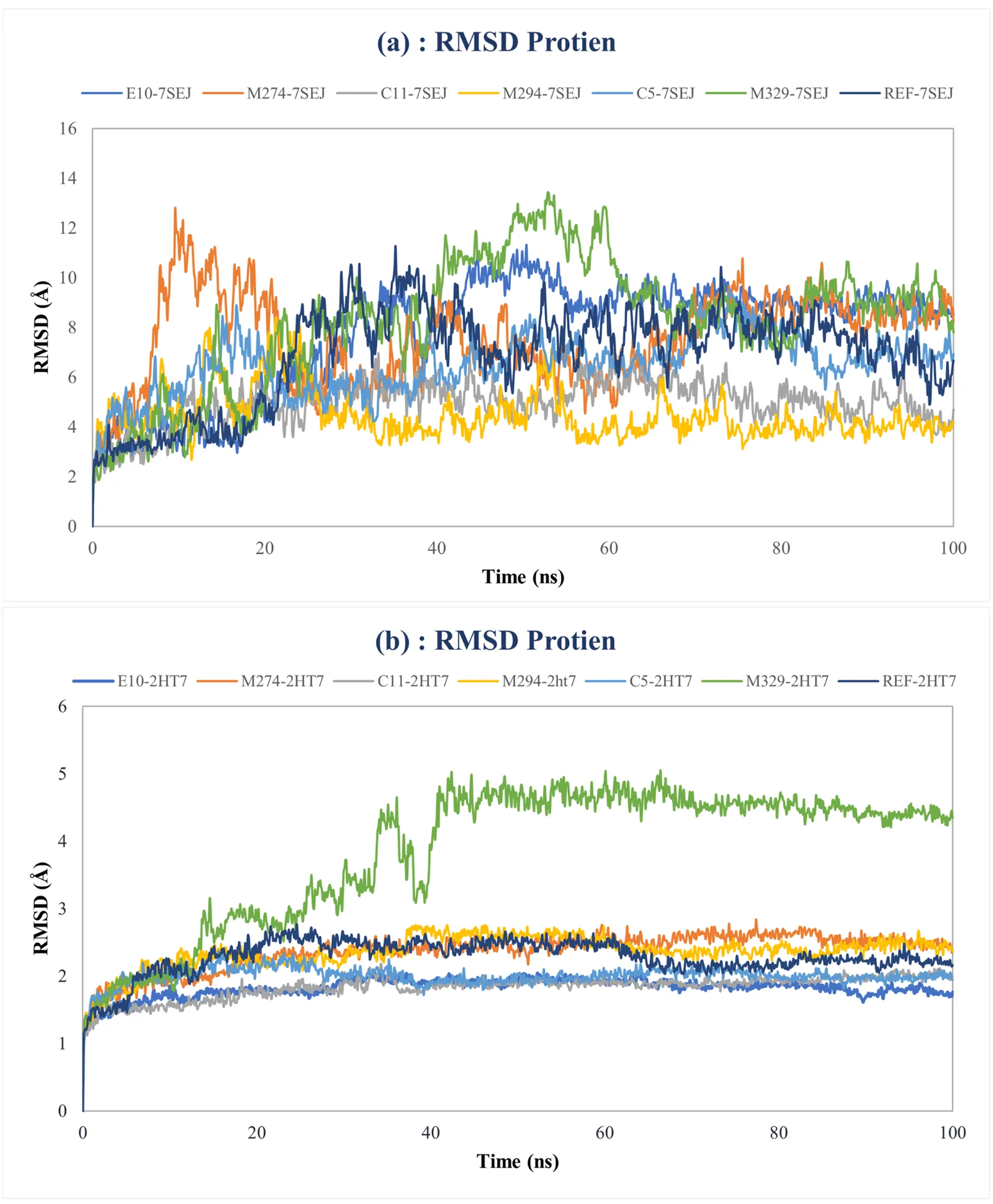
(a): The RMSD profile of the target protein (7SEJ) in complex with the studied ligands. (b): The RMSD profile of the target protein (2HT7) in complex with the studied ligands.

**RMSD of the protein:** The RMSD (root mean square deviation) profiles of each system are shown in Fig 3(a,b) as a function of simulation time. Regarding the target protein (7SEJ), the first 30 nanoseconds of the trajectory exhibit a clear common pattern: all systems show a significant increase in RMSD, resulting from a period of structural adjustment and equilibration of the protein-ligand complexes. After this adaptation period, the profiles show a relatively flat section where only limited and acceptable fluctuations are present, indicating that conformational stability is achieved for these systems throughout the simulation. Notably, the RMSD values of the systems in the presence of ligand M294 and C11 are consistently lower than those of the other complexes or the reference system, suggesting that ligand M294 and C11 confer greater structural stability to the complexes formed.

Regarding the second target (2HT7), after an initial adaptation period during which the protein adjusted to the simulation environment and RMSD values gradually increased, all simulated systems reached a dynamic equilibrium state. The trajectories converged after equilibration and remained relatively constant for the remainder of each simulation. Although all ligand-bound systems exhibited comparable trajectory stability, the presence of ligand M329 produced slightly higher RMSD values than the other ligand-bound systems, suggesting that ligand M329 induces a greater conformational change in the protein, potentially due to an adjustment of the binding region and/or the protein substructure located in or near this region. These data demonstrate that, on a global scale, the second target possesses a greater degree of conformational stability resulting from ligand binding than does the first target; however, in the specific case of ligand M329, it appears to have induced a modest amount of structural alteration compared to the other protein-ligand complex system observed in this study.

**Fig 4:**
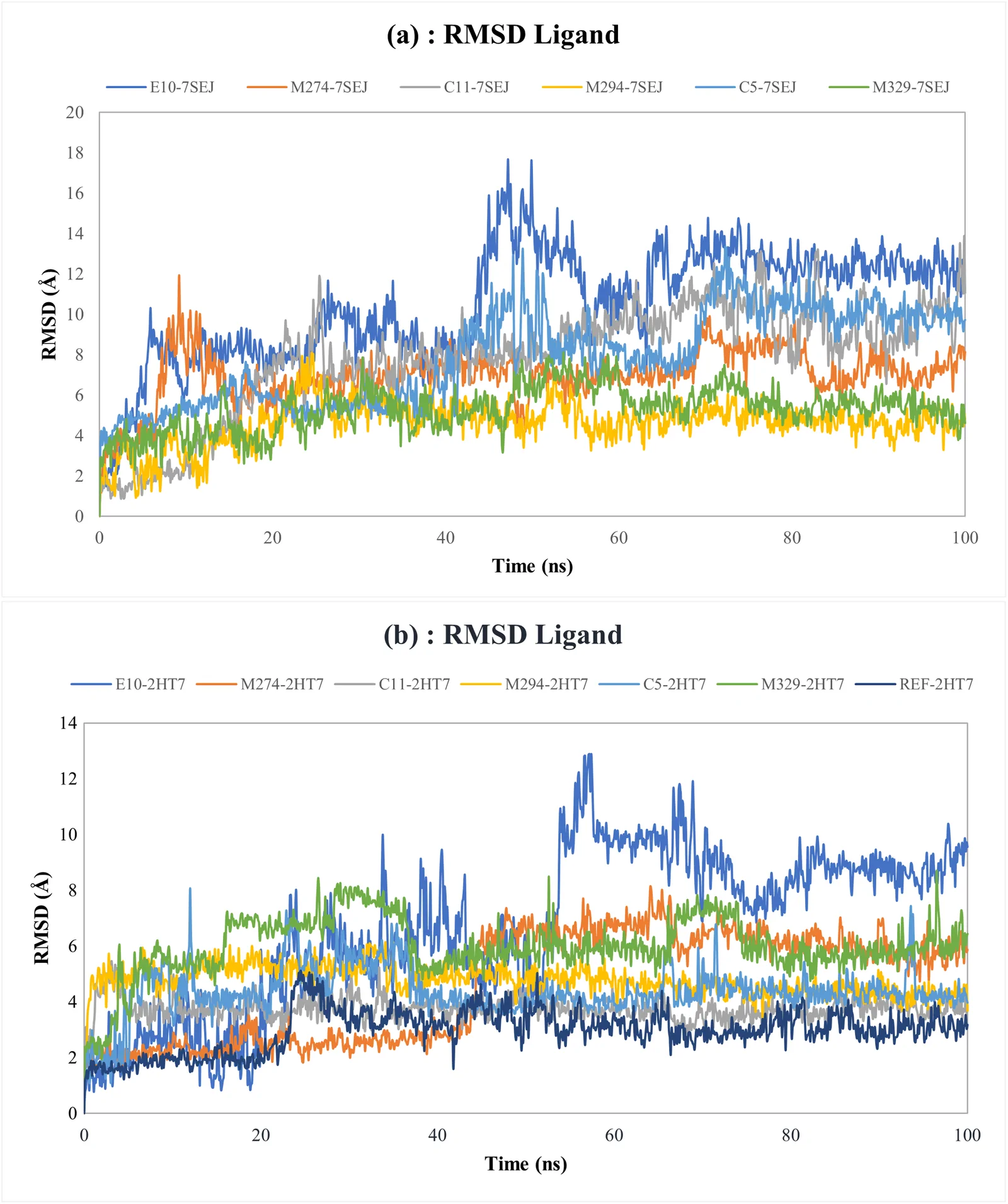
(a): The RMSD profile of the ligands in complex with 7SEJ during the simulation. (b): The RMSD profile of the ligands in complex with 2HT7 during the simulation.

RMSD of the ligand: Regarding the RMSD values of the ligands (Fig 4(a)), in complex with the protein 7SEJ, ligands M294, M329, and M274 exhibited initial fluctuations at the beginning of the simulation, then stabilized and maintained a relatively stable conformation at the binding site. Conversely, other ligands with RMSD values showed greater mobility, indicating that these ligands exhibited greater flexibility, weaker binding interactions, or less stable interactions with the active site.

The RMSD profiles of all the ligands studied in complex with the 2HT7 protein during the simulation were found to be relatively stable (Fig 4(b)). This suggests that these ligands maintain fairly constant binding conformations within the active site of the target protein. After a short equilibration period, the RMSD trajectories of each of these ligands reached a plateau, exhibiting only small fluctuations. This implies that the ligands are tightly anchored in their binding pockets and maintain stable intermolecular interactions with the amino acids located in the protein binding site. Conversely, ligand E10 exhibited comparatively larger RMSD fluctuations over time than all the other compounds tested, which could indicate increased mobility and more pronounced conformational changes within the binding cavity.

#### 3.3.2. RMSF profiles.

**Fig 5:**
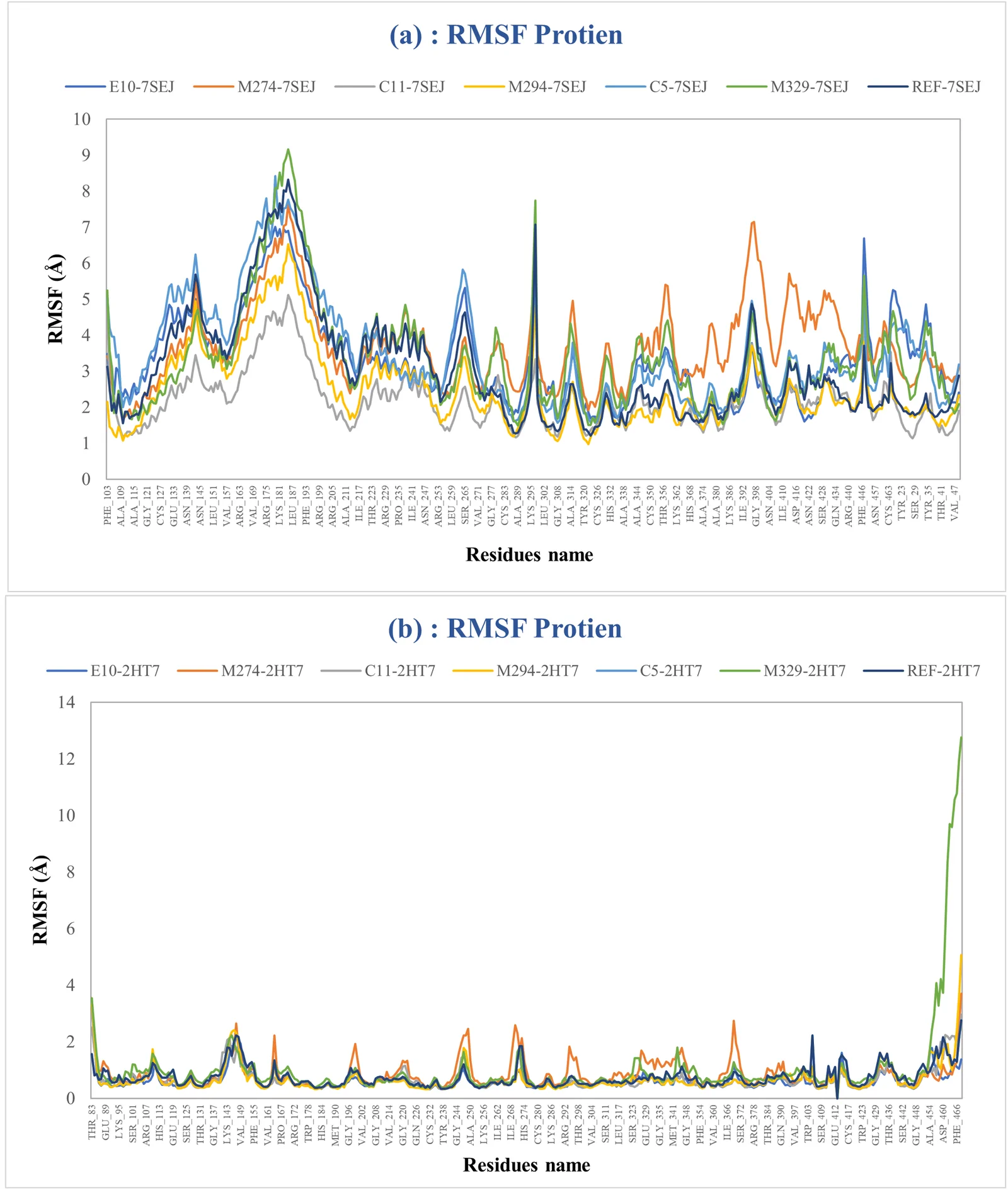
(a): The RMSF profile of the protein 7SEJ in complex with the ligands studied. (b): The RMSF profile of the protein 2HT7 in complex with the ligands studied.

Fig 5(a,b) shows the RMSF of the systems studied as a function of the simulation time. Most residues of protein 7SEJ fluctuated between 1 and 4 Å according to the RMSF analysis results. This indicates that all proteins in each simulated system maintained an overall stable structure. A marked flexibility peak was observed for amino acids 160–190 in all complexes, demonstrating the presence of a highly mobile loop. The amplitude of fluctuations in the C11-F and M294-7SEJ complexes was lower than that of the REF-7SEJ complex, suggesting a significant stabilizing effect due to ligand binding. Furthermore, the REF-7SEJ complex exhibited significant fluctuations around amino acid 300, fluctuations that disappeared in the complex systems, indicating stiffening induced by ligand binding. Additionally, fluctuations in the 380−420 region of the M274-7SEJ complex were significantly greater than those of the REF-7SEJ complex, suggesting a potential local conformational change or weak stabilizing interactions. In general, ligand binding induces a reduction in residue movement relative to the reference structure. C11-7SEJ and M294-7SEJ ligands produced greater stabilizing effects on protein dynamics than all other complexes.

The 2HT7 protein is relatively rigid and exhibits very low intrinsic flexibility, as indicated by its consistently low RMSF values across all residues. This low fluctuation is likely related to the maintenance of a stable tertiary structure in 2HT7 throughout the simulation, as well as its very low local mobility. The relative rigidity of the overall structure may allow the protein to maintain its functional conformation in physiological environments, which is important for interactions with ligands and its ability to bind them. The limited mobility at the residue level suggests that the active and binding sites of 2HT7 are largely conserved, thus providing a structural context favorable to ligand interaction.

#### 3.3.3. Protein–ligand contact analysis.

**Fig 6:**
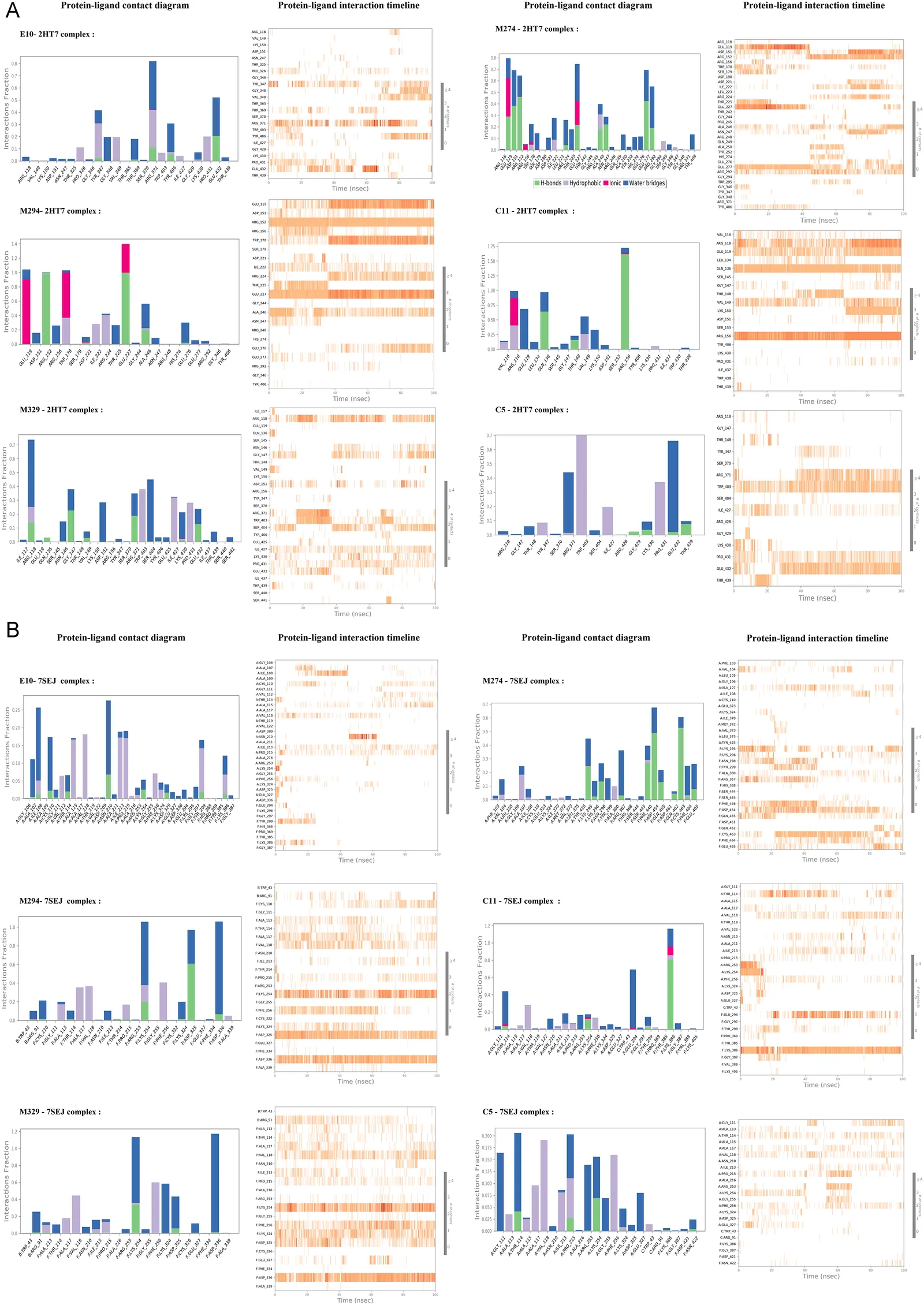
Contact diagrams and Interaction timelines of Protein-Ligand Complexes for selected ligands with (a) NA (PDB ID: 2HT7) and (b) F (PDB ID: 7SEJ).

The results of the protein-ligand contact histogram and the chronology of interactions for each complex studied with the different viral targets NA (2HT7) and F (7SEJ) are presented in Fig 6(a,b) as a function of simulation time. Analysis of the interactions in the M294–2HT7 complex revealed several key residues involved in binding, among which GLU119, ARG152, TRP178, and GLU227 showed dominant, persistent, and stable interactions over time, with significant contributions from hydrogen bonds, ionic interactions, and hydrophobic interactions. These interactions contribute to the stabilization of ligand M294 in the active site of the 2HT7 structure, allowing it to remain firmly anchored during the simulation, which is consistent with the low RMSD values observed for the ligand. For the C11–2HT7 complex, ligand stabilization was maintained through major interactions with several key residues, notably ARG156, GLN136, ARG118, and GLU119, forming hydrogen bonds, ionic interactions and hydrophobic interactions with the 2HT7 protein, thus indicating their crucial role in ligand binding within the active site of the protein. These results confirm the structural stability observed in previous analyses. Ligand M274 establishes a diverse network of interactions with protein 2HT7 through several hydrogen bonds, ionic interactions, and water bridges, mainly with residues GLU119, ASP151, GLU227, ARG152, GLU277, and ARG292. These interactions contribute to the stabilization of ligand M274 within the binding site of protein 2HT7. However, they appear to be intermittent and moderately dynamic over time, suggesting moderate stability.

In addition, ligand M329 forms a complex with 2HT7 through hydrogen bonds, several water bridges, and hydrophobic interactions, involving several residues such as ARG118, GLY147, ARG371, and TRP403. Among these interactions, the ARG118 residue appears to be the most frequent with a significant interaction fraction, playing a key role in the stabilization and binding of M329 within 2HT7. However, according to the interaction timeline of this complex, the majority of interactions appear to be alternative and time-limited, giving the binding of ligand M329 a more dynamic character compared to the other ligands studied.

On the other hand, analysis of the interactions of M294–7SEJ complex reveals stability similar to that observed with the NA(2HT7) receptor, characterized by persistent interactions with a high fraction of interactions involving several key residues. These interactions include hydrogen bonds, water bridges, and hydrophobic interactions, particularly with residues ASP325, ASP336, and LYS254. Additional moderate interactions also contribute to the stabilization of the ligand in the binding site of the 7SEJ protein, such as those involving PHE256, VAL118, and ALA117, throughout the simulation. All of these interactions contribute to the solid maintenance of the ligand’s anchorage in the active site of the 7sej protein. Regarding the C11–7SEJ complex, analysis of the interactions shows that the C11 ligand interacts mainly with the LYS386 residue, forming a persistent hydrogen bond interaction. Other interactions, notably ionic and water bridges, are also observed with residues GLY294 and THR114. Overall, thanks to these interactions, and particularly the one with residue LYS386, the C11 ligand remains firmly anchored in the active site of the 7SEJ protein.

Ligand M274 interacts with protein 7SEJ via a diverse network of interactions including hydrogen bonds, water bridges, and hydrophobic interactions, primarily with residues ASP454, CYS463, PHE446, LYS295, among others. Some of these interactions are persistent throughout much of the simulation, while others appear more dynamically over time. Overall, these interactions contribute to the maintenance of ligand M274 within the binding site of protein 7SEJ, suggesting moderate binding stability. Furthermore, ligand M329 maintains its stability with the 7SEJ protein mainly through hydrogen bonds, water bridges, and hydrophobic interactions, involving key residues such as LYS254 and ASP336, which form persistent and relatively continuous interactions over time. In addition, other interactions appear intermittently, via residues PHE256, ASP325, LYS324, and VAL118, indicating a moderately stable binding mode.

In contrast, analysis of the interactions of ligand E10 with the two viral targets 2HT7 and 7SEJ also reveals several interactions, including hydrogen bonds, water bridges, and hydrophobic interactions. However, these interactions are not continuous over time, indicating greater mobility of ligand E10 within the binding site of both receptors. This observation is consistent with the higher RMSD values of the ligand observed during the simulation.

Overall, the molecular dynamics analyses demonstrated that the selected protein–ligand complexes exhibited varying degrees of stability throughout the simulations, with M294 and C11 showing the most persistent interactions and the most favorable stability profiles against both viral targets. To further quantify the binding strength of these complexes, MM-PBSA binding free-energy calculations were subsequently performed.

#### 3.3.4. MM-PBSA binding free energy analysis.

To further validate the stability and binding affinity of the selected protein–ligand complexes, binding free energies were estimated using the Molecular Mechanics Poisson–Boltzmann Surface Area (MM-PBSA) method based on the equilibrated MD simulation trajectories. Unlike RMSD and RMSF analyses, which primarily evaluate structural stability and flexibility during the simulation, MM-PBSA provides a quantitative energetic assessment of protein–ligand binding, where more negative binding free-energy values indicate stronger and more favorable interactions.

As summarized in Table 6, distinct binding-energy profiles were observed for the two viral targets. For neuraminidase (2HT7), C11 exhibited the most favorable binding free energy (−42.91 kcal/mol), outperforming both M294 (−20.83 kcal/mol) and the reference inhibitor oseltamivir (−23.21 kcal/mol), suggesting a stronger interaction with the catalytic binding site. Conversely, for the HMPV fusion protein (7SEJ), M294 displayed the lowest binding free energy (−33.49 kcal/mol), followed by C11 (−25.57 kcal/mol), with both compounds exhibiting more favorable binding energies than the reference antiviral ribavirin (−18.23 kcal/mol).

**Table 6:** Binding free-energy calculations using the MM-PBSA method.

Compound	NA	F
M294	−20.83	**−33.49**
C11	**−42.91**	−25.57
Oseltamivir	−23.21	–
Ribavirin	–	−18.23

These energetic results are fully consistent with the molecular dynamics analyses. Throughout the simulations, M294 and C11 maintained stable protein conformations, limited residue fluctuations, and persistent interactions with key binding-site residues, indicating stable complex formation. The MM-PBSA calculations therefore provide quantitative energetic support for the structural observations obtained from the MD simulations. Although each compound exhibited its strongest predicted affinity toward a different viral target—C11 for neuraminidase and M294 for the HMPV fusion protein—both compounds consistently demonstrated favorable performance throughout the complete computational workflow, further supporting their selection as the most promising broad-spectrum antiviral lead candidates.

Overall, the final prioritization of lead compounds was based on the sequential integration of molecular docking, drug-likeness evaluation, ADMET and toxicity profiling, molecular dynamics simulations, and MM-PBSA binding free-energy calculations (Table 7). Among the six compounds retained after ADMET assessment, M294 and C11 consistently satisfied all selection criteria by combining favorable multi-target docking performance, acceptable predicted pharmacokinetic and toxicological profiles, stable structural behavior throughout the MD simulations, and favorable MM-PBSA binding free energies toward both viral targets. Although C5, M274, and M329 also exhibited stable interactions with one or both proteins, their overall performance across the integrated computational workflow was comparatively less consistent, supporting the prioritization of M294 and C11 as the final broad-spectrum antiviral lead compounds.

**Table 7:** Sequential prioritization framework for the selection of the final broad-spectrum antiviral lead compounds.

Screening step	Selection criterion	Outcome
Initial phytoconstituent library	Literature-derived compounds	121 compounds
Molecular docking	Multi-target binding affinity	37 compounds
Drug-likeness	Lipinski + Veber	13 compounds
ADMET & toxicity	Favorable pharmacokinetic and toxicity profile	E10, M294, M329, C11, C5, M274
Molecular dynamics and MM-PBSA	RMSD, RMSF, protein–ligand interactions, binding free-energy analysis	M294 and C11 identified as final broad-spectrum lead compounds

## 4. Conclusion

This study explored the broad-spectrum antiviral potential of phytoconstituents, which have been the subject of several previous studies, targeting two proteins involved in respiratory infections: neuraminidase (NA) from influenza A virus and fusion protein (F) from HMPV. A molecular modeling approach was adopted, integrating molecular docking, ADMET property prediction, molecular dynamics (MD) simulations, and MM-PBSA binding free-energy calculations. The results of molecular docking revealed promising multi-target binding potential for a set of 37 phytoconstituents, with binding affinities higher than those of the reference molecules, oseltamivir and ribavirin. After a thorough evaluation of their ADMET drug profile, including bioavailability analysis and analysis of pharmacokinetic and toxicological properties, phytoconstituents numbered E10, M294, M329, C11, C5, and M274 were selected as candidate compounds due to their good bioavailability, favorable ADME profile, and absence of major violations concerning their toxicological properties. Molecular dynamics (MD) simulations, further supported by MM-PBSA binding free-energy calculations, demonstrated the stable binding behavior of compounds M294 (apigenin, a Moroccan medicinal plant derived from *Petroselinum crispum* L., Apiaceae) and C11 (tetrahydrocannabinolic acid, extracted from *Cannabis sativa* L., Cannabaceae) with the two viral targets NA and F over the duration of the simulation. Although C5 (tetrahydrocannabivarin), M274 (Sidisterone), and M329 (Jaceosidin) also showed favorable target-specific stability during the MD simulations, their overall performance across the integrated computational workflow was less consistent than that of M294 and C11, which emerged as the most promising broad-spectrum antiviral candidates. Overall, this computational study identified compounds M294 and C11 as promising natural broad-spectrum antiviral candidates targeting both influenza A neuraminidase and the HMPV fusion protein. The originality of this work lies in the integrated computational evaluation of previously reported antiviral phytoconstituents against two distinct respiratory viruses through a multi-target strategy combining molecular docking, ADMET and toxicity profiling, molecular dynamics simulations, and MM-PBSA binding free-energy analysis, thereby providing a rational framework for prioritizing candidates for future experimental validation.

However, the identified compounds should be considered computational lead candidates. Their predicted antiviral potential requires experimental validation, particularly through in vitro antiviral assays, followed by in vivo investigations.

## Supporting information

S1 Table Names of the studied phytoconstituents, their sources, and chemical structures. (DOCX)

S2 Table Molecular docking binding energies (BE) of the studied phytoconstituents targeting both NA (PDB ID: 2ht7) and HMPV-F (PDB ID: 7sej) proteins compared with reference compounds. (DOCX)

S3 Table Physicochemical properties of the studied phytoconstituents based on to Lipinski’s and Veber’s rules in comparison with reference compounds. (DOCX)
