# Supplementary material for: Repositioning antiviral phytoconstituents as broad-spectrum inhibitors of influenza A neuraminidase and human metapneumovirus fusion protein
Source: PLoS One. 2026 Sep 2;21(9):e0348517. doi: 10.1371/journal.pone.0348517 (PMC13537546; doi:10.1371/journal.pone.0348517)
Supplement: Supplementary file 2 [file pone.0348517.s006.pdf]

Recommendation: Accept with minor revisions

General assessment

This manuscript presents a well-executed in silico study repositioning antiviral phytoconstituents as potential broad-spectrum inhibitors of influenza A neuraminidase (NA) and HMPV fusion protein (F). The work is clearly written, methodologically sound, and builds logically on the authors' previous investigations of related phytochemical libraries. The docking, ADME/T profiling, and MD simulations are generally appropriate, and the conclusions are supported by the presented data. I believe the manuscript can be accepted after the authors address the minor issues listed below, which mainly concern clarity, completeness of methodological description, and alignment between text, tables, and figures.

1. Graphical abstract

The graphical abstract is clear but would benefit from additional quantitative detail. I recommend that the authors indicate the number of compounds at key stages of the workflow (initial library, post-docking/ADMET filtering, and final hits) directly in the figure. Adding these counts will improve transparency of the screening process and help readers quickly understand the scale and progression of the study from raw phytochemicals to proposed lead candidates.

2. Rationale for target selection (NA and F)

In the Introduction, the rationale for selecting neuraminidase (NA) and HMPV fusion protein (F) as targets—particularly in the context of the authors' prior work on hemagglutinin (HA) and SARS-CoV-2 Mpro—is not fully articulated. The authors should expand the relevant paragraph to: (i) explain how targeting NA and F complements or extends the previous HA/Mpro findings, (ii) justify these proteins as logical choices for a broad-spectrum antiviral strategy, and (iii) clarify how they address distinct, non-redundant steps in the viral life cycle (entry/fusion vs. release). This will strengthen the conceptual framework of the study.

3. Ligand preparation: optimization details

The Methods state that "for the ligands, the MMFF94 force field and the gradient descent algorithm implemented in Avogadro were applied." To enhance reproducibility, the authors should specify the convergence criteria used for ligand geometry optimization (e.g., energy or gradient threshold, maximum number of steps, and any stopping conditions). These details are important for others wishing to replicate the ligand preparation workflow.

4. ADMET: definition of "optimal water solubility"

In the ADMET section, the phrase "all the compounds studied… have optimal water solubility" is vague. The term "optimal" should be replaced or explicitly mapped to the solubility categories used in Table 4 (e.g., soluble, moderately soluble, partially soluble). I suggest that the authors (i) state how many compounds fall into each category and (ii) clearly describe the exceptions (C11, E59, M22) using the same terminology as in Table 4, to ensure consistency between text and table.

5. Justification for retaining C11, C5, and M274

The conclusion that "compounds C11, C5, and M274 are generally considered acceptable… justifying their inclusion in the list of candidates for further exploration" is reasonable but should be made more explicit. I recommend that the authors:

- Restate the specific toxicity issues for each compound (C11 and C5: only LD50 < 1000 mg/kg; M274: only a carcinogenicity flag, with other endpoints inactive).
- Clearly indicate that these are **in silico** leads for further investigation, not ready drug candidates, given the presence of these safety flags.
- Optionally note that their overall ADME/T profiles are more favorable than some discarded molecules and at least one reference drug, which supports their retention as exploratory leads.

These clarifications will make the selection criteria more transparent.

6. ProTox-II outputs: "active/inactive" and probability

In Table 5, ProTox-II results are reported only as "active" or "inactive" for each toxicity endpoint (neurotoxicity, cytotoxicity, carcinogenicity, mutagenicity, hepatotoxicity), without explanation. The authors should:

- Clarify in the Methods or a table footnote that "active" denotes a positive toxicity prediction for that endpoint and "inactive" denotes a negative prediction.
- Include the ProTox-II prediction probabilities (or accuracy/confidence scores) for these calls, at least for key candidate compounds, and specify the probability threshold considered meaningful (e.g., >0.70).

Reporting and interpreting these probabilities will help readers assess the robustness of the toxicity predictions.

7. Figure 3a: missing legends

In Figure 3a (RMSD of 7sej complexes), some curves appear to lack corresponding entries in the legend, which makes it difficult to identify each protein–ligand trajectory. The

authors should ensure that every RMSD curve is clearly assigned to a ligand in the legend (or directly annotated) and that the legend matches the ligands discussed in the Results.

Overall recommendation:

The manuscript addresses an interesting and relevant topic, the data are solid, and the required changes are mainly clarifications and minor presentation improvements. Provided the authors satisfactorily address the above points, I recommend **acceptance after minor revision**.
