# Supplementary material for: Repositioning antiviral phytoconstituents as broad-spectrum inhibitors of influenza A neuraminidase and human metapneumovirus fusion protein
Source: PLoS One. 2026 Sep 2;21(9):e0348517. doi: 10.1371/journal.pone.0348517 (PMC13537546; doi:10.1371/journal.pone.0348517)
Supplement: Supplementary file 3 [file pone.0348517.s005.pdf]

PLOS One

Repositioning antiviral phytoconstituents as broad-spectrum inhibitors of influenza A neuraminidase and human metapneumovirus fusion protein

--Manuscript Draft--

**Manuscript Number:**	PONE-D-26-18338
**Article Type:**	Research Article
**Full Title:**	Repositioning antiviral phytoconstituents as broad-spectrum inhibitors of influenza A neuraminidase and human metapneumovirus fusion protein
**Short Title:**	Repositioning antiviral phytoconstituents as inhibitors of influenza
**Corresponding Author:**	Samir Chtita<br>Hassan II University of Casablanca Faculty of Sciences Ben M'Sik: Universite Hassan II de Casablanca Faculte des Sciences Ben M'Sik<br>Casa, 5000 MOROCCO
**Keywords:**	Antiviral activity; Influenza A; Neuraminidase (NA); Molecular Dynamics; Molecular Docking; ADMET; Phytoconstituents; Broad-spectrum inhibitors
**Abstract:**	Viral respiratory infections still exist as a major global health concern. Influenza A viruses and human metapneumoviruses are among the main pathogens responsible for infections that can progress to severe acute respiratory forms. Despite the availability of certain therapeutic options, the need for effective treatment remains an issue. The exploration of innovative approaches based on medicinal plants, capable of simultaneously targeting several viruses, represents a promising strategic option for limiting the viral activity of these pathogens. In this work, a set of antiviral phytoconstituents, which have been the subject of several previous studies, were studied using molecular modeling techniques to exploit their broad-spectrum antiviral potential against influenza A virus neuraminidase (NA) (PDB ID: 2ht7) and the fusion protein (F) of HMPV (PDB ID: 7sej). Molecular docking analyses identified 37 phytoconstituents with multi-target binding affinity for NA and F receptors, with energy scores higher than those of oseltamivir and ribavirin, used as reference molecules. In addition, ADMET prediction, including bioavailability assessment and analysis of pharmacokinetic and toxicological properties, identified several compounds with favorable profiles, notably compounds E10, M294, M329, C11, C5, and M274. A more in-depth analysis using molecular dynamics simulation was performed to evaluate the stability of complexes formed between the the best compounds and the viral targets studied. The results obtained suggest that compounds M294 and C11 are likely to be potential inhibitors of the NA and F viral receptors of influenza A and HMPV. However, these results require experimental validation, particularly through in vivo and in vitro studies.
**Order of Authors:**	Nouh Mounadi
	Hassan Nour
	Mhammed El Kouali
	Abdelouahid Samadi
	Samir Chtita
**Additional Information:**	
**Question**	**Response**
**Financial Disclosure**<br><br>Enter a financial disclosure statement that describes the sources of funding for the work included in this submission. Review the submission guidelines for detailed requirements. View published research	Yes

Powered by Editorial Manager® and ProduXion Manager® from Aries Systems Corporation

articles from *PLOS ONE* for specific examples.<br><br>This statement is required for submission and **will appear in the published article** if the submission is accepted. Please make sure it is accurate.<br><br>**Funded studies**<br>Enter a statement with the following details:<br>• Initials of the authors who received each award<br>• Grant numbers awarded to each author<br>• The full name of each funder<br>• URL of each funder website<br>• Did the sponsors or funders play any role in the study design, data collection and analysis, decision to publish, or preparation of the manuscript?<br><br>Did you receive funding for this work?	
Please add funding details.<br>as follow-up to "**Financial Disclosure**<br><br>Enter a financial disclosure statement that describes the sources of funding for the work included in this submission. Review the submission guidelines for detailed requirements. View published research articles from *PLOS ONE* for specific examples.<br><br>This statement is required for submission and **will appear in the published article** if the submission is accepted. Please make sure it is accurate.	This work was supported by the Zayed Bin Sultan Center for Health Sciences and UAEU-University of Sharjah joint Grant (#12R282).

Powered by Editorial Manager® and ProduXion Manager® from Aries Systems Corporation

**Funded studies**<br>Enter a statement with the following details:<br>• Initials of the authors who received each award<br>• Grant numbers awarded to each author<br>• The full name of each funder<br>• URL of each funder website<br>• Did the sponsors or funders play any role in the study design, data collection and analysis, decision to publish, or preparation of the manuscript?<br><br>Did you receive funding for this work?"	
Please select the country of your main research funder (please select carefully as in some cases this is used in fee calculation).<br>as follow-up to "**Financial Disclosure**<br><br>Enter a financial disclosure statement that describes the sources of funding for the work included in this submission. Review the submission guidelines for detailed requirements. View published research articles from *PLOS ONE* for specific examples.<br><br>This statement is required for submission and **will appear in the published article** if the submission is accepted. Please make sure it is accurate.<br><br>**Funded studies**<br>Enter a statement with the following details:<br>• Initials of the authors who received each award<br>• Grant numbers awarded to each author<br>• The full name of each funder<br>• URL of each funder website<br>• Did the sponsors or funders play any role in the study design, data collection and analysis, decision to publish, or preparation of the manuscript?<br><br>Did you receive funding for this work?"	UNITED ARAB EMIRATES - AE
**Competing Interests**	The authors declare no competing financial interests.

Use the instructions below to enter a competing interest statement for this submission. On behalf of all authors, disclose any competing interests that could be perceived to bias this work—acknowledging all financial support and any other relevant financial or non-financial competing interests.<br><br>This statement is required for submission and **will appear in the published article** if the submission is accepted. Please make sure it is accurate and that any funding sources listed in your Funding Information later in the submission form are also declared in your Financial Disclosure statement.<br><br>View published research articles from *PLOS ONE* for specific examples.<br><br>**NO authors have competing interests**<br><br>Enter: *The authors have declared that no competing interests exist.*<br><br>**Authors with competing interests**<br><br>Enter competing interest details beginning with this statement:<br><br>*I have read the journal's policy and the authors of this manuscript have the following competing interests: [insert competing interests here]*<br><br>* typeset	
**Ethics Statement**<br><br>Enter an ethics statement for this submission. This statement is required if the study involved:<br><br>• Human participants<br>• Human specimens or tissue<br>• Vertebrate animals or cephalopods<br>• Vertebrate embryos or tissues	N/A

• Field research<br><br>Write "N/A" if the submission does not require an ethics statement.<br><br>General guidance is provided below. Consult the submission guidelines for detailed instructions. **Make sure that all information entered here is included in the Methods section of the manuscript.**	

**Format for specific study types**<br><br>**Human Subject Research (involving human participants and/or tissue)**<br>• Give the name of the institutional review board or ethics committee that approved the study<br>• Include the approval number and/or a statement indicating approval of this research<br>• Indicate the form of consent obtained (written/oral) or the reason that consent was not obtained (e.g. the data were analyzed anonymously)<br><br>**Animal Research (involving vertebrate animals, embryos or tissues)**<br>• Provide the name of the Institutional Animal Care and Use Committee (IACUC) or other relevant ethics board that reviewed the study protocol, and indicate whether they approved this research or granted a formal waiver of ethical approval<br>• Include an approval number if one was obtained<br>• If the study involved *non-human primates*, add *additional details* about animal welfare and steps taken to ameliorate suffering<br>• If anesthesia, euthanasia, or any kind of animal sacrifice is part of the study, include briefly which substances and/or methods were applied<br><br>**Field Research**<br><br>Include the following details if this study involves the collection of plant, animal, or other materials from a natural setting:<br>• Field permit number<br>• Name of the institution or relevant body that granted permission	
**Data Availability**<br><br>Authors are required to make all data underlying the findings described fully available, without restriction, and from the time of publication. PLOS allows rare exceptions to address legal and ethical concerns. See the PLOS Data Policy and FAQ for detailed information.	Yes - all data are fully available without restriction

A Data Availability Statement describing where the data can be found is required at submission. Your answers to this question constitute the Data Availability Statement and **will be published in the article**, if accepted.<br><br>**Important:** Stating 'data available on request from the author' is not sufficient. If your data are only available upon request, select 'No' for the first question and explain your exceptional situation in the text box.<br><br>Do the authors confirm that all data underlying the findings described in their manuscript are fully available without restriction?	
**Describe where the data may be found in full sentences. If you are copying our sample text, replace any instances of XXX with the appropriate details.**<br><br>• If the data are **held or will be held in a public repository**, include URLs, accession numbers or DOIs. If this information will only be available after acceptance, indicate this by ticking the box below. For example: *All XXX files are available from the XXX database (accession number(s) XXX, XXX.).*<br>• If the data are all contained **within the manuscript and/or Supporting Information files**, enter the following: *All relevant data are within the manuscript and its Supporting Information files.*<br>• If neither of these applies but you are able to provide **details of access elsewhere**, with or without limitations, please do so. For example:<br><br>*Data cannot be shared publicly because of [XXX]. Data are available from the XXX Institutional Data Access / Ethics Committee (contact via XXX) for researchers who meet the criteria for access to confidential data.*<br><br>*The data underlying the results presented in the study are available from (include the name of the third party*	All relevant data are within the manuscript and its Supporting Information files.

Powered by Editorial Manager® and ProduXion Manager® from Aries Systems Corporation

and contact information or URL).<br>• This text is appropriate if the data are owned by a third party and authors do not have permission to share the data.<br><br>* typeset	
Additional data availability information:	

Click here to access/download;Manuscript;Manuscript.docx

Repositioning antiviral phytoconstituents as broad-spectrum inhibitors of influenza A neuraminidase and human metapneumovirus fusion protein

Nouh Mounadi [1], Hassan Nour [1], M'hammed El Kouali [1],
Abdelouahid Samadi [(2,*)], Samir Chtita [(*, 1)]
[1] Laboratory of analytical and molecular chemistry, Faculty of Sciences Ben M'Sik, Hassan II University of Casablanca, Morocco.
[2] Department of Chemistry, College of Science, United Arab Emirates University, Al Ain P.O. Box 15551, United Arab Emirates.
* *Correspondence:* samadi@uaeu.ac.ae, samirchtita@gmail.com

Abstract:
Viral respiratory infections still exist as a major global health concern. Influenza A viruses and human metapneumoviruses are among the main pathogens responsible for infections that can progress to severe acute respiratory forms. Despite the availability of certain therapeutic options, the need for effective treatment remains an issue. The exploration of innovative approaches based on medicinal plants, capable of simultaneously targeting several viruses, represents a promising strategic option for limiting the viral activity of these pathogens. In this work, a set of antiviral phytoconstituents, which have been the subject of several previous studies, were studied using molecular modeling techniques to exploit their broad-spectrum antiviral potential against influenza A virus neuraminidase (NA) (PDB ID: 2ht7) and the fusion protein (F) of HMPV (PDB ID: 7sej). Molecular docking analyses identified 37 phytoconstituents with multi-target binding affinity for NA and F receptors, with energy scores higher than those of oseltamivir and ribavirin, used as reference molecules. In addition, ADMET prediction, including bioavailability assessment and analysis of pharmacokinetic and toxicological properties, identified several compounds with favorable profiles, notably compounds E10, M294, M329, C11, C5, and M274. A more in-depth analysis using molecular dynamics simulation was performed to evaluate the stability of complexes formed between the the best compounds and the viral targets studied. The results obtained suggest that compounds M294 and C11 are likely to be potential inhibitors of the NA and F viral receptors of influenza A and HMPV. However, these results require experimental validation, particularly through in vivo and in vitro studies.

Keywords: Antiviral activity; Influenza A; Neuraminidase (NA); Molecular Dynamics; Molecular Docking; ADMET; Phytoconstituents; Broad-spectrum inhibitors.

Graphical Abstract:

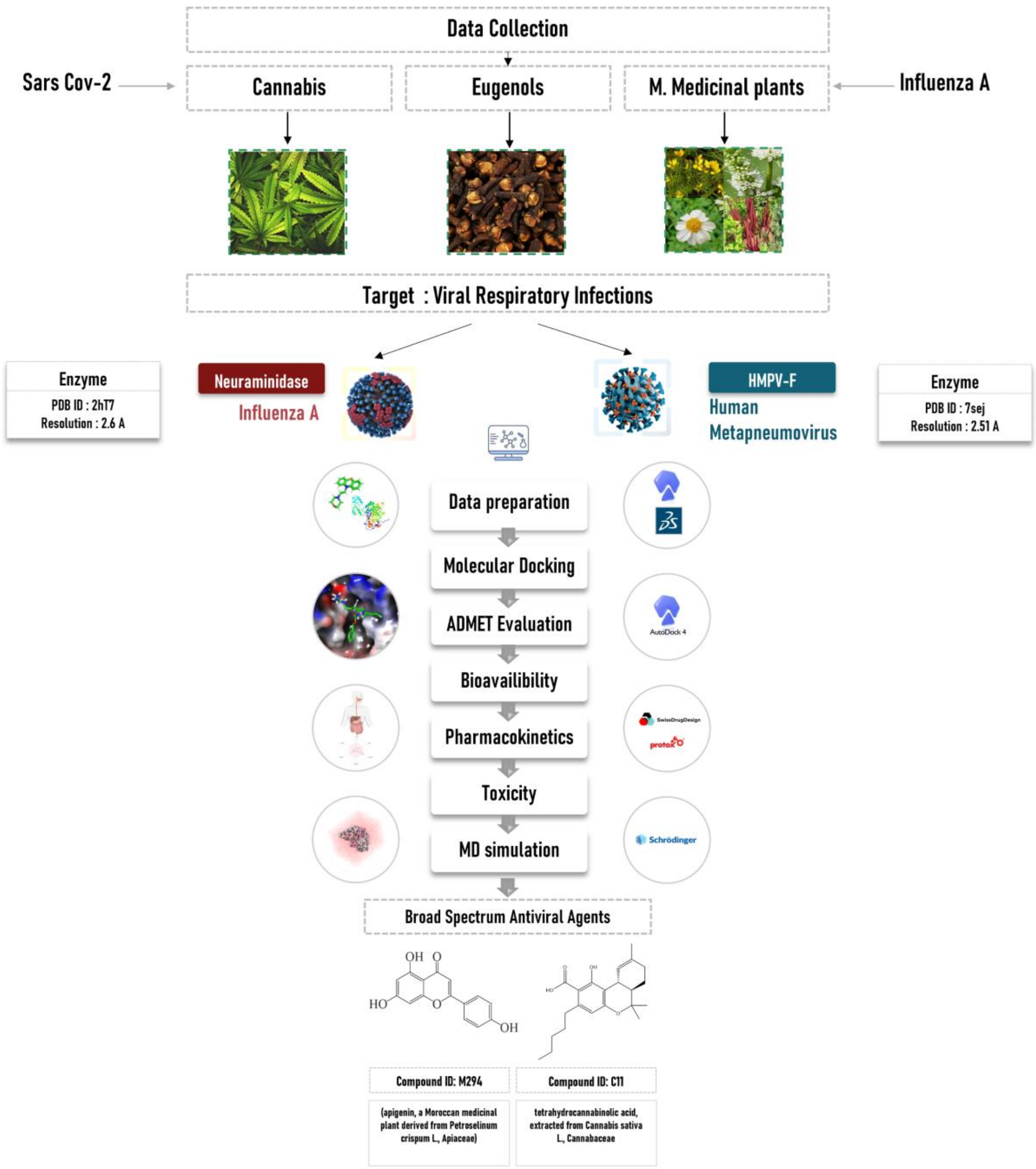

1. Introduction

Viral respiratory infections remain a global public health problem. Influenza A virus and human metapneumovirus (HMPV) are among the agents responsible for severe acute respiratory problems, leading to high mortality and morbidity, particularly among vulnerable individuals such as children and the elderly **[1,2]**. These viruses have a considerable epidemiological impact, influenza A, in particular, has been known to cause major pandemics. This is due to its high mutation rate and its ability to genetically reassort, which makes it highly resistant to the immune system and existing antiviral drugs **[3]**. Despite the availability of vaccines and antivirals, treatment options aimed at limiting their pathogenic actions remain limited, especially when it comes to the emergence of new variants. These evolving characteristics highlight the need for further research to develop new, effective, broad-spectrum antiviral therapeutic strategies. Historically identified in 2001, HMPV belongs to the Pneumoviridae family and is recognized as an important agent responsible for upper and lower respiratory tract infections. Influenza A and HMPV commonly share certain symptoms of respiratory infections, ranging from mild upper respiratory tract infections to serious infections such as bronchitis or pneumonia, which makes clinical diagnosis more difficult without laboratory tests.

Neuraminidase (NA) is an enzyme and a well-known viral target, having been the subject of extensive research and development of treatments for influenza A, such as oseltamivir and zanamivir. It plays a key role in the release of viral particles by cleaving the sialic acid residues present on the surface of host cells **[4]**. In addition, the fusion protein (F) plays an important role in HMPV virus entry by mediating fusion between the viral membrane and the host cell membrane **[5]**. An approach targeting both NA and F simultaneously is therefore an innovative and promising strategy for limiting viral activity of these two pathogens.

Currently, antiviral treatments are often specific to a single virus and may have side effects. As for the HMPV virus, there is no specific treatment recommended to date. These limitations highlight the need to explore broad-spectrum antivirals with very low toxicity and good tolerance. Phytoconstituents are an interesting natural source, offering advantages due to their safety and well-known effects.

Building on several of our previous studies, which led to the discovery of natural antivirals against SARS-CoV-2 and influenza A (H3N2) and which have been the subject of numerous other studies on various activities, this project aims to evaluate the multi-target, broad-spectrum antiviral potential of these phytoconstituents. By integrating molecular modeling with

pharmacological knowledge, the objective is to identify promising candidates for the development of new therapeutic strategies targeting both influenza A (NA) and HMPV (F).

2. Materials and Methods

2.1 Dataset sources

2.1.1 Dataset Background

In our previous studies, we investigated several phytochemical compounds derived from Moroccan medicinal plants belonging to different botanical families, as well as cannabis and eugenol derivatives **[6-13]**. This diverse database has been the subject of numerous antiviral studies, particularly against influenza A (H3N2) virus by targeting hemagglutinin, SARS-CoV-2 by targeting the main protease (Mpro), as well as other biological activities. These studies have identified several phytoconstituents with significant antiviral potential. In this study, we focused simultaneously on influenza A neuraminidase and HMPV fusion (F) protein, in an approach aimed at promoting a broad-spectrum multi-target strategy. The goal of this approach is to promote the use of natural resources as safe and well-tolerated solutions, while deepening our understanding of the molecular interactions involved in viral entry/fusion and viral release mechanisms. The structures and information, such as systematic names, identifiers (IDs), and families, relating to the database of the different phytoconstituents analyzed in the present study are presented in Supplementary **Table S1**.

2.1.2 Biological Target

The biological targets covered by this study include two proteins derived from viruses known to cause respiratory infections. The first is neuraminidase (NA), a key protein involved in viral release and spread (PDB identifier: 2ht7) **[14,15]**. The second is the fusion protein (F), designed to stabilize its pre-fusion conformation. This protein plays a key role in the entry of the HMPV virus by mediating the fusion between the viral membrane and the host cell membrane. (PDB identifier: 7sej). Both crystallographic structures were extracted from the Protein Data Bank (PDB) due to their functional relevance and high-resolution data, making them suitable for molecular modeling and molecular dynamics simulations (**Figure 1**).

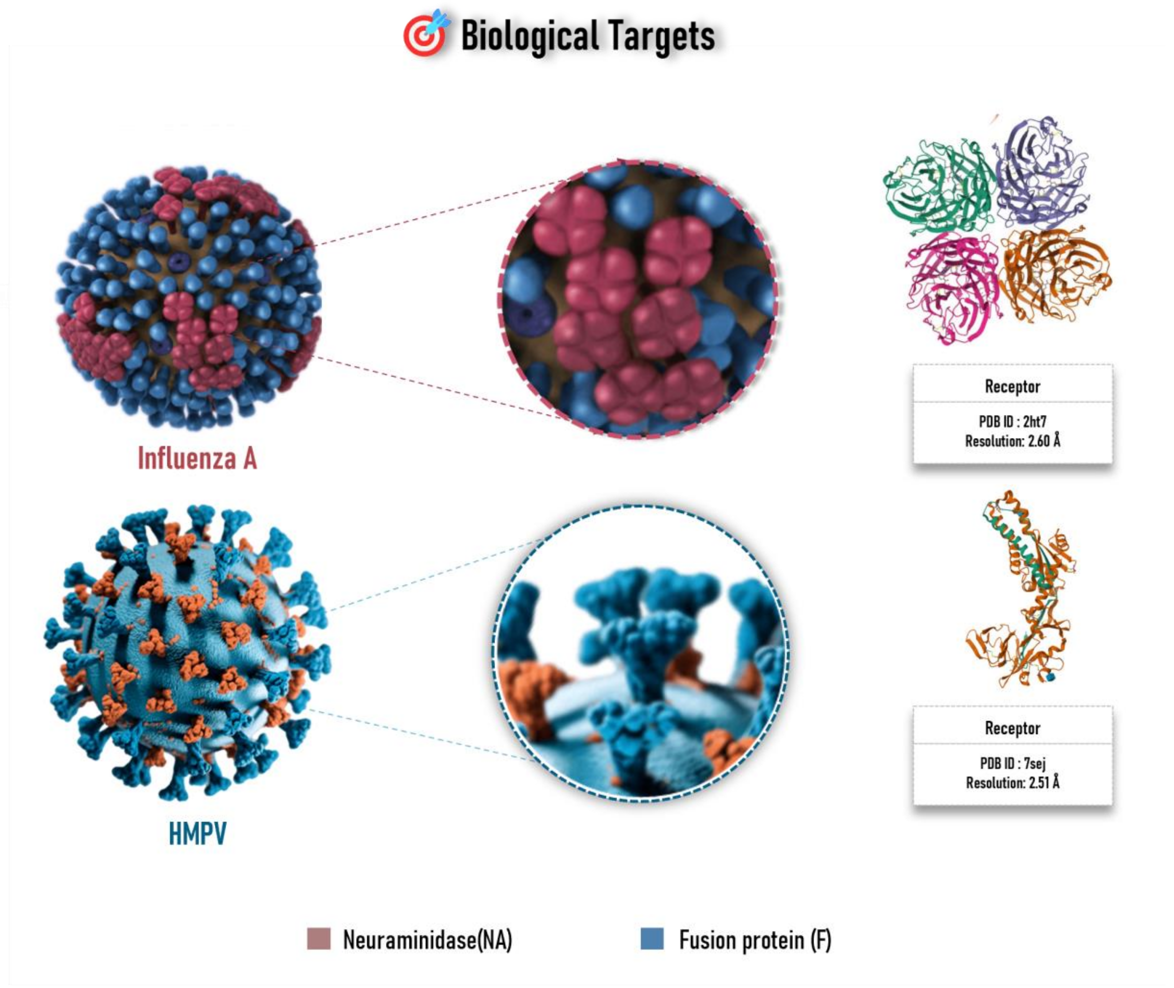

Figure 1. Representation of the studied biological structures of the two viral proteins: the HMPV fusion protein (F) and influenza A neuraminidase (NA), with their corresponding PDB identifiers.

2.2 Dataset Preparation

2.2.1 Ligands and Protein Preparation

In order to ensure optimal results, good conformational stability of the system comprising ligands and proteins must be guaranteed. To this end, these structures have been optimized. For the ligands, the MMFF94 force field and the gradient descent algorithm implemented in Avogadro were applied. They were prepared by assigning Gasteiger charges and adding polar hydrogens. For the proteins, the structures of NA (PDB ID: 2ht7) and HMPV-F (ID: 7sej) were prepared by first removing water molecules and crystallized co-ligands, then adding the missing hydrogen atoms and assigning Kollman charges. All these preparations were performed using AutoDockTools**[16]**.

2.2.2 Grid Box Setup

In order to ensure accurate coverage of the active site of NA (PDB ID: 2ht7), the grid parameters representing the protein-ligand binding region were deduced using Discovery Studio 2021 **[17]**. The center of the grid was determined based on the position of the co-crystallized ligand within the binding pocket, namely Oseltamivir, a well-known NA inhibitor and a drug widely used in the treatment of influenza A. For the F protein (PDB ID: 7sej), the grid center corresponding to the primary protease was determined using Autodocktools **[16]**. The docking grids and their dimensions, illustrated in **Figure 2**, provide sufficient space to allow for ligand flexibility and exhaustive exploration of the entire binding cavity.

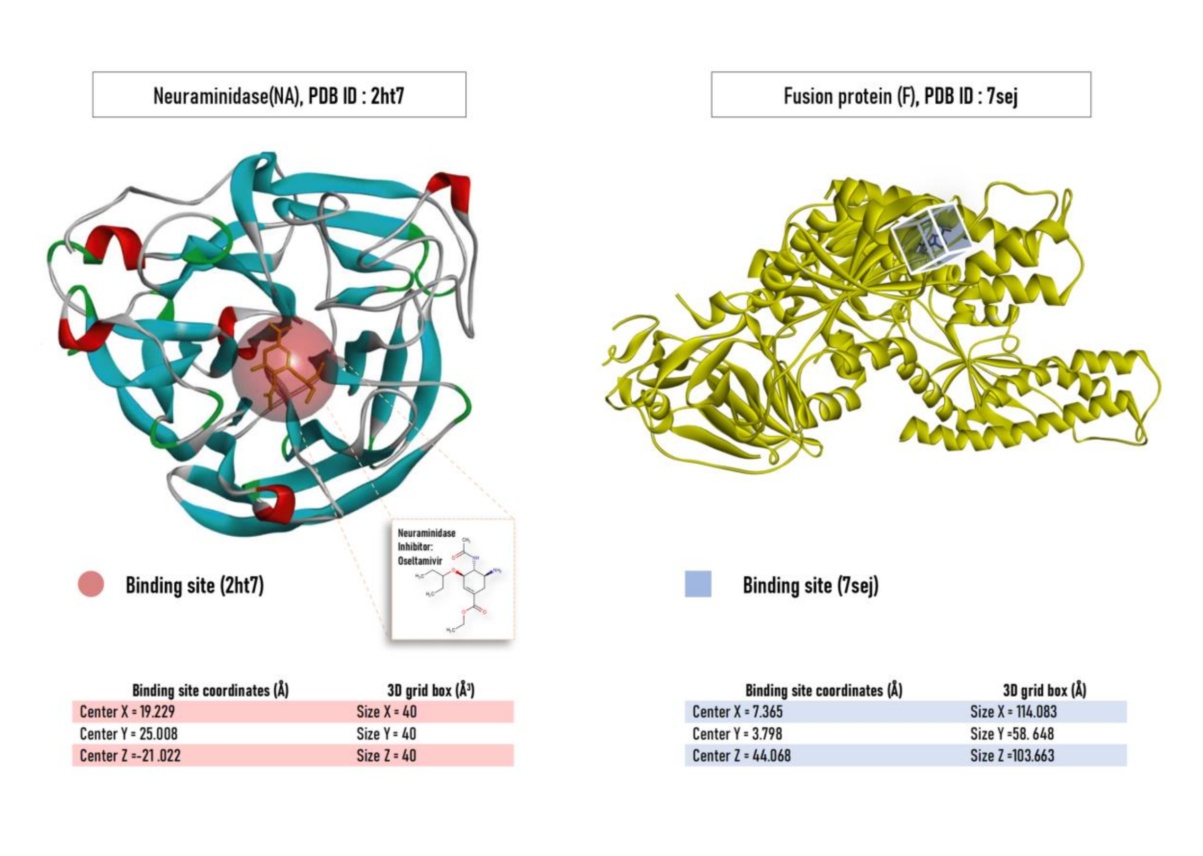

Figure 2. Three-dimensional visualization of the binding cavities selected for molecular docking, along with their corresponding coordinates: (a) 2ht7 neuraminidase receptor and (b) 7sej Fusion receptor.

2.3 Molecular Docking Procedure of Receptor–Ligand Complexes

Following the preparation of the database, molecular docking was performed using AutoDock Vina software (version 1.1.2), with the aim of evaluating ligand-protein affinity through the analysis of binding energies and corresponding interactions **[16]**. All phytoconstituents were docked onto the two viral targets studied (NA and F). The results were evaluated based on

energy scores, binding mode, and finally the nature of the molecular interactions established with key and important residues of the active sites.

2.4 ADMET analysis

ADMET analysis (absorption, distribution, metabolism, excretion, and toxicity), including bioavailability and pharmacokinetic and toxicological properties, was performed using two online tools: SwissADME and ProTox-II (UN, 2013) **[18,19]**. This analysis was performed following the selection of molecules with a high affinity for both viral receptors studied. The first step in this process was to identify the molecules most suitable for oral administration. To this end, bioavailability was studied according to Lipinski's and Veber's rules **[20,21]**. **Table 1** describes the various criteria and parameters on which we based our decision at this stage. Next, all of the selected molecules that showed favorable bioavailability compared to the reference drugs were analyzed in terms of their pharmacokinetic and toxicological profiles, with the aim of proposing molecules that were more suitable for safe medicinal use. Finally, the best selection was chosen for further study using molecular dynamics simulations.

Table 1. Crietrias of Lipinski's and Veber's rules

Rule	Parameter	Criterion
**Lipinski's Rule of Five**	Hydrogen bond donors (HBD)	≤ 5
	Hydrogen bond acceptors (HBA)	≤ 10
	Molecular weight (MW)	≤ 500 Daltons
	Log P (partition coefficient)	≤ 5
**Veber's Rule**	Rotatable bonds (RB)	≤ 10
	Topological polar surface area (TPSA)	≤ 140 Å²

2.5 Molecular dynamics simulation

Molecular dynamics simulations were conducted using Maestro, part of the Schrödinger software suite, coupled with Desmond, in order to examine the stability of complexes, ligand-protein behavior, and their interactions over time **[22]**. Following the selection of the most favorable candidates through various molecular docking and ADMET analyses, these compounds were prepared in complex with the viral targets studied. System preparation consisted of the removal of water molecules and structural optimization, followed by solvation in an orthorhombic box with a 10 Å buffer around the complex, using the simple point charge (SPC) water model, as well as neutralization by adding Na^+ and Cl^- ions at a concentration of 0.15 M **[23,24]**. After system relaxation, the simulations were subjected to a 1 ns equilibration phase under the NVT ensemble, followed by a 100 ns production run under the NPT ensemble, at a temperature set at 300 K and a pressure of 1 atm **[25]**. Trajectories were analyzed to extract

key structural and dynamic parameters, including root-mean-square deviation (RMSD), root-mean-square fluctuation (RMSF), and ligand–protein contact diagrams.

3. Results and discussion

3.1 Molecular docking simulation

A molecular docking study was conducted on the entire ligand library, specifically targeting the active sites of NA (PDB ID: 2ht7) and the fusion protein (PDB ID: 7sej), representing multi-targeted influenza A and HMPV viruses, respectively. The objective of this study is to evaluate the potential of these phytoconstituents to exhibit a broad antiviral spectrum by examining their binding capacity and potential affinity for the biological targets studied. In this context, two drugs were carefully selected as comparative references for the candidate ligands: oseltamivir (NA) and ribavirin (F).

The docking results revealed that, among the 121 phytoconstituents tested, several ligands outperformed the two different references studied in terms of energy scores (oseltamivir: -6.2 kcal/mol, ribavirin: -5.8 kcal/mol), exhibiting more negative binding energies (**Table S2**). Among them, 37 ligands showed a particularly high affinity, simultaneously targeting both NA and F receptors. For these ligands, the criteria used were: BE(NA) ≤ −7.4 kcal/mol; BE(F) ≤ −7.0 kcal/mol (**Table 2**). These results suggest that several phytochemical compounds could interact more effectively with the catalytic site of NA and F, indicating potentially superior inhibitory activity compared to reference drugs.

Table 2. Molecular docking binding energies (BE) of the top selected ligands targeting both NA (PDB ID: 2ht7) and HMPV-F proteins (PDB ID: 7sej) compared with reference compounds.

Famille / Origine	Ligand	BE (kcal/mol) (PDB ID: 2ht7)	BE (kcal/mol) (PDB ID: 7sej)	Criteria Met
Cannabis (Cannabinoïdes)	C5	-7.7	-7.4	☑
	C7	-7.6	-7.4	☑
	C11	-8.0	-7.3	☑
Eugenol derivatives	E10	-8.2	-8.5	☑
	E18	-7.9	-7.1	☑
	E19	-8.0	-7.3	☑
	E51	-9.5	-9.2	☑
	E57	-7.4	-7.1	☑
	E58	-8.4	-7.2	☑
	E59	-8.3	-7.7	☑
Marocain Medicinal Plants	M110	-10.4	-9.1	☑
	M17	-10.0	-8.2	☑
	M293	-10.0	-9.1	☑
	M319	-9.1	-8.0	☑
	M322	-9.1	-8.1	☑
	M292	-9.0	-8.1	☑
	M324	-9.0	-8.7	☑

	M378	-9.0	-8.4	☑
	M269	-8.9	-8.5	☑
	M285	-8.9	-8.1	☑
	M16	-8.7	-8.0	☑
	M320	-8.7	-8.7	☑
	M321	-8.7	-8.5	☑
	M286	-8.6	-8.2	☑
	M274	-8.5	-8.2	☑
	M109	-8.4	-7.3	☑
	M111	-8.4	-8.1	☑
	M270	-8.4	-9.1	☑
	M267	-8.1	-8.6	☑
	M268	-8.1	-8.6	☑
	M295	-8.1	-8.3	☑
	M148	-8.1	-7.0	☑
	M22	-8.0	-7.3	☑
	M276	-8.0	-7.2	☑
	M329	-8.0	-7.3	☑
	M294	-7.4	-8.0	☑
	M81	-7.4	-7.4	☑
**References**		**-6.2**[a]	**-5.8**[b]	

BE: binding energies, **Reference**[a:] Oseltamivir, **Reference**[b:] Ribavirin

3.2 ADMET predictions

3.2.1 Evaluation of Drug-likeness properties

Based on the results of molecular docking, 37 ligands were selected for evaluation of drug-likeness properties. The objective of this step is to select the compounds best suited for drug development, compared to the drug references studied, and presenting optimal oral bioavailability. To do this, Lipinski's and Veber's 5 rules were used as mandatory selection criteria. **Table S3** presents the predicted data from this study. According to the results obtained, only 13 ligands, summarized in **Table 3**, were favoured and selected because they meet all the considered criteria without any violations, and demonstrated a predicted oral bioavailability superior to that of both references drug. This promising selection was taken for further analysis.

Table 3. Physicochemical properties of the top selected ligands meeting Lipinski's and Veber's rules in comparison with reference compounds.

**Ligand**	**Formula**	**Lipinski and Veber parameters**						**Number of violations**		**Criteria Met**
		**Mw**	**LogP**	**RB**	**HBA**	**HBD**	**PSA**	**Lipinski**	**Veber**	
**C5**	C19H26O2	286.41	4.68	2	2	1	29.46	0	0	☑
**C11**	C22H30O4	358.47	4.94	5	4	2	66.76	0	0	☑
**E10**	C22H3O7	379.26	0.78	5	7	3	80.14	0	0	☑
**E18**	C17H2NO2	252.20	2.40	4	2	1	30.49	0	0	☑

**E19**	C18H2NO3	280.21	2.30	5	3	1	39.72	0	0	✓
**E51**	C27H2N2O5	434.32	1.53	6	6	2	82.53	0	0	✓
**E57**	C19ClNO2	309.66	3.42	5	3	0	31.35	0	0	✓
**E59**	C27HClN2O4	452.76	2.83	9	5	1	59.00	0	0	✓
**M285**	C27H42O7	478.62	1.76	2	7	5	127.59	0	0	✓
**M274**	C24H32O6	416.51	1.93	1	6	3	104.06	0	0	✓
**M22**	C15H10O6	286.24	1.73	1	6	4	111.13	0	0	✓
**M329**	C17H14O7	330.29	2.11	3	7	3	109.36	0	0	✓
**M294**	C15H10O5	270.24	2.11	1	5	3	90.90	0	0	✓
**Ref.**[1]	C16H28N2O4	312.40	1.38	9	5	2	90.65	0	0	N/A
**Ref.**[2]	C8H12N4O5	244.20	-2.14	3	7	4	143.72	0	1	N/A
			≤ 5	≤10	≤ 10	≤ 5	≤ 140	≤2	≤1	

3.2.2 Evaluation of pharmacokinetics and toxicity properties

Next, the selection obtained from the bioavailability assessment was evaluated in terms of pharmacokinetic properties and toxicity in order to highlight the therapeutic potential of the compounds and their suitability as leading candidates for broad-spectrum antiviral applications. This study includes the evaluation of several parameters, namely water solubility (WS), Pain alerts, gastrointestinal absorption (G.I.A), blood-brain barrier permeability (BBB), inhibition of cytochrome P-450 enzymes (CYP2D6, CYP3A4, CYP1A2, CYP2C19, and CYP2C9), and skin permeability (S.P). As for toxicity, the prediction parameters considered include LD50, neurotoxicity, cytotoxicity, carcinogenicity, mutagenicity, and hepatotoxicity.

According to the results summarized in **Table 4**, it appears that all the compounds studied, with the exception of C11, and compound No. E59, have optimal water solubility, without any Pain alert (except for compound No. M22). Furthermore, they are predicted to be impermeable to the BBB, with the exception of C5, and compound No. E57, while exhibiting high gastrointestinal absorption and acceptable skin permeability, ranging from −10.0 to −1.0 cm/s. Assessing the inhibitory power of cytochrome P450 enzymes is essential when selecting drug candidates. This assessment provides information on whether these candidates are likely to inhibit the enzymes responsible for breaking down active substances. Such inhibition can lead to an accumulation of these active substances and/or their metabolites in the human body, with extremely toxic consequences.

According to the results obtained, compounds M285, M274, E10, E51, and E18 are the most favorable in terms of non-inhibition of cytochrome P450 enzymes, with the exception of compounds E10, E51, and E18, which show a single inhibition related to the CYP2C9 enzyme. Compound C11 was predicted to inhibit CYP2C9 and CYP3A4 enzymes, while compound E59 was predicted to inhibit CYP3A4 and CYP2C19 enzymes, respectively.

Compounds C5, E57, and E19 reported inhibition violations involving three different enzymes. Indeed, these compounds were predicted to be common inhibitors of CYP2C19 and CYP2C9. In addition, compounds C5 is estimated to be inhibitors of CYP2D6, while compound E57 appears to be an inhibitor of CYP1A2. Finally, compound E19 does not inhibit the CYP2D6 enzyme. Compounds M329, M294, and M22 were predicted to be inhibitors of the CYP2D6, CYP3A, and CYP1A2 enzymes, with additional inhibition of CYP2C9 for compound M329.

Table 4. Pharmacokinetic properties (ADME) of the top selected ligands compared with reference compounds.

**Ligand**	**Pharmacokinetic parameters**									
	**WS**	**Pain alert**	**G.I. A**	**BBB**	**CYP 2D6**	**CYP3 A4**	**CYP1 A2**	**CYP2C 19**	**CYP2 C9**	**S.P (cm/s)**
**C5**	M.S	0	High	Yes	Yes	No	No	Yes	Yes	-3.87
**C11**	P.S	0	High	No	No	Yes	No	No	Yes	-3.48
**E10**	S	0	High	No	No	No	No	No	Yes	-6.91
**E18**	M.S	0	High	No	No	No	No	No	Yes	-4.77
**E19**	M.S	0	High	No	Yes	No	No	Yes	Yes	-4.96
**E51**	M.S	0	High	No	No	No	No	No	Yes	-6.14
**E57**	M.S	0	High	Yes	No	No	Yes	Yes	Yes	-4.36
**E59**	P.S	0	High	No	No	No	No	Yes	Yes	-5.02
**M285**	S	0	High	No	No	No	No	No	No	-8.73
**M274**	S	0	High	No	No	No	No	No	No	-8.07
**M22**	M.S	1	High	No	Yes	Yes	Yes	No	No	-6.25
**M329**	M.S	0	High	No	Yes	Yes	Yes	No	Yes	-6.14
**M294**	M.S	0	High	No	Yes	Yes	Yes	No	No	-5.80
**Ref.1**	V.S	0	High	No	No	No	No	No	No	-7.42
**Ref.2**	V.S	0	Low	No	No	No	No	No	No	-9.10

M. S: Moderately soluble, **P. S**: Poorly soluble, **WS**: Water Solubility, **BBB:** Blood–Brain Barrier, **G.I.A**: Gastrointestinal Absorption, **S.P**: Skin Permeation, **Reference[1]:** Oseltamivir, **Reference[2]:** Ribavirin

The evaluation of toxicity parameters is a crucial step in an ADMET study, as it allows for the prediction of safe use of candidates in drug development. In this regard, low and acceptable toxicity is highly desirable. At this stage, the information obtained from these parameters makes it possible to reduce the number of selected compounds and differentiate them from the two reference drugs, while taking into account the other criteria evaluated.

According to the results obtained (**Table 5)**, compounds E10, M329, and M294 showed no violation of toxicity parameters. Compounds C11, and C5 meet all predicted criteria, except for LD50 (less than 1000mg/kg). Compound M274 showed a single violation related to carcinogenicity, while compounds E19 and E59 were predicted to be neurotoxic and hepatotoxic. Compounds E51 and E57 showed low LD50; in addition, compound E51 appears to be neurotoxic and cytotoxic, while compound E57 is neurotoxic and hepatotoxic.

Finally, compounds M285, E18, and M22 were excluded from selection for further analysis due to unfavourable toxicological profiles. Compound M285 has an extremely low LD50 (34 mg/kg) and predicted carcinogenicity, while compound M22 is predicted to be both mutagenic and carcinogenic. In addition, compound E18 has several violations, including cytotoxicity, carcinogenicity, mutagenicity, and hepatotoxicity.

Table 5. Toxicity prediction of the top selected ligands compared with reference compounds.

**Ligand**	**Toxicity**							
	**LD50 (mg/kg)**	**Neuro-toxicity**	**Cyto-toxicity**	**Carcino-genicity**	**Muta-genicity**	**Hepato-toxicity**	**Classe**	**Yes/No**
**C5**	482	Inactive	Inactive	Inactive	Inactive	Inactive	4	☑
**C11**	500	Inactive	Inactive	Inactive	Inactive	Inactive	4	☑
**E10**	5000	Inactive	Inactive	Inactive	Inactive	Inactive	5	☑
**E18**	1600	Active	Inactive	Active	Active	Active	4	☒
**E19**	1190	Active	Inactive	Inactive	Inactive	Active	4	☒
**E51**	709	Active	Active	Inactive	Inactive	Inactive	4	☒
**E57**	500	Active	Inactive	Inactive	Inactive	Active	4	☒
**E59**	1190	Active	Inactive	Inactive	Inactive	Active	4	☒
**M285**	34	Inactive	Inactive	Active	Inactive	Inactive	2	☒
**M274**	2450	Inactive	Inactive	Active	Inactive	Inactive	5	☑
**M22**	3919	Inactive	Inactive	Active	Active	Inactive	5	☒
**M329**	3919	Inactive	Inactive	Inactive	Inactive	Inactive	5	☑

**M294**	2500	Inactive	Inactive	Inactive	Inactive	Inactive	5	☑
**Ref.1**	260	Inactive	Inactive	Inactive	Inactive	Inactive	3	N/A
**Ref.2**	2700	Active	Inactive	Active	Inactive	Inactive	5	N/A

Reference[1]: Oseltamivir, **Reference[2]:** Ribavirin

In general, ADMET analysis led to a compromise in the selection of the most favourable candidates in terms of their pharmacokinetic and toxicological profiles. Compared to the two reference drugs studied, Oseltamivir, which has an extremely low LD50, and Ribavirin, which appears to be both neurotoxic and carcinogenic, with low gastrointestinal absorption, compounds E10, M294, and M329 stand out with impeccable toxicological properties, no violations observed, and an acceptable ADME profile, placing them in first position as the best candidates. Furthermore, compounds C11, C5, and M274 are generally considered acceptable, given their favorable ADME profile and the absence of numerous violations related to their toxicological properties (only one violation was detected concerning either LD50 or carcinogenicity), justifying their inclusion in the list of candidates for further exploration. On the other hand, compounds showing multiple toxicity violations and interactions with CYP450 enzymes, such as compounds M285, E18, and M22, E51, E57, E19, and E59, are discarded.

3.3 Molecular dynamics simulation

3.3.1 RMSD profiles

RMSD of the protein:

The RMSD (root mean square deviation) profiles of each system are shown in **Figure 3** as a function of simulation time. Regarding the target protein (7sej), the first 30 nanoseconds of the trajectory exhibit a clear common pattern: all systems show a significant increase in RMSD, resulting from a period of structural adjustment and equilibration of the protein-ligand complexes. After this adaptation period, the profiles show a relatively flat section where only limited and acceptable fluctuations are present, indicating that conformational stability is achieved for these systems throughout the simulation. Notably, the RMSD values of the systems in the presence of ligand M294 and C11 are consistently lower than those of the other complexes or the reference system, suggesting that ligand M294 and C11 confer greater structural stability to the complexes formed.

Regarding the second target (2ht7), after an initial adaptation period during which the protein adjusted to the simulation environment and RMSD values gradually increased, all simulated systems reached a dynamic equilibrium state. The trajectories converged after equilibration and remained relatively constant for the remainder of each simulation. Although all ligand-bound

systems exhibited comparable trajectory stability, the presence of ligand M329 produced slightly higher RMSD values than the other ligand-bound systems, suggesting that ligand M329 induces a greater conformational change in the protein, potentially due to an adjustment of the binding region and/or the protein substructure located in or near this region. These data demonstrate that, on a global scale, the second target possesses a greater degree of conformational stability resulting from ligand binding than does the first target; however, in the specific case of ligand M329, it appears to have induced a modest amount of structural alteration compared to the other protein-ligand complex system observed in this study.

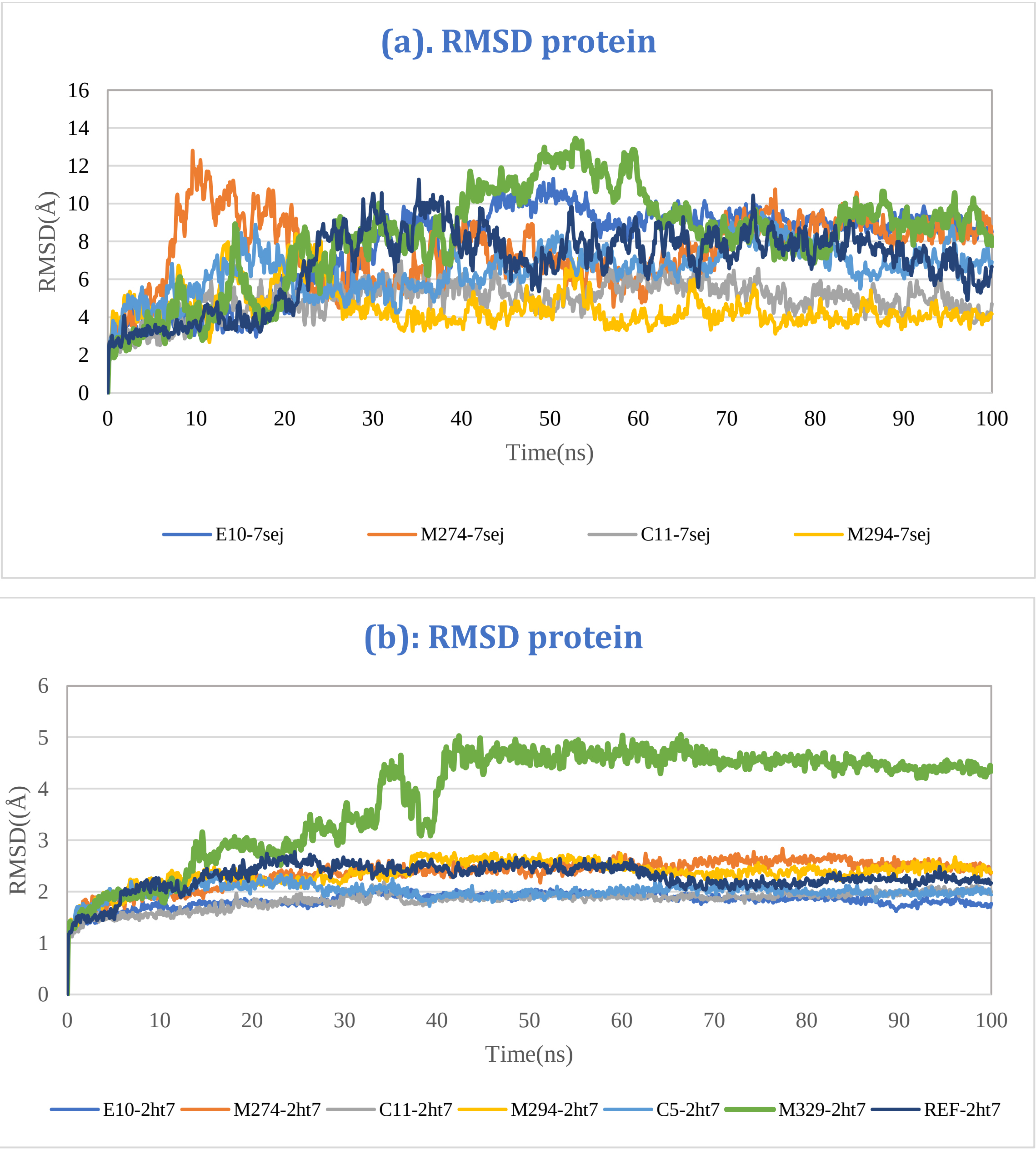

Figure 3. **(a)**: The RMSD profile of the target protein (7sej) in complex with the studied ligands. **(b)**: The RMSD profile of the target protein (2ht7) in complex with the studied ligands

RMSD of the ligand:

Regarding the RMSD values of the ligands (**Figure 4(a)**), in complex with the protein 7sej, ligands M294, M329, and M274 exhibited initial fluctuations at the beginning of the simulation, then stabilized and maintained a relatively stable conformation at the binding site. Conversely, other ligands with RMSD values showed greater mobility, indicating that these ligands exhibited greater flexibility, weaker binding interactions, or less stable interactions with the active site.

The RMSD profiles of all the ligands studied in complex with the 2ht7 protein during the simulation were found to be relatively stable. This suggests that these ligands maintain fairly constant binding conformations within the active site of the target protein. After a short equilibration period, the RMSD trajectories of each of these ligands reached a plateau, exhibiting only small fluctuations. This implies that the ligands are tightly anchored in their binding pockets and maintain stable intermolecular interactions with the amino acids located in the protein binding site. Conversely, ligand E10 exhibited comparatively larger RMSD fluctuations over time than all the other compounds tested, which could indicate increased mobility and more pronounced conformational changes within the binding cavity.

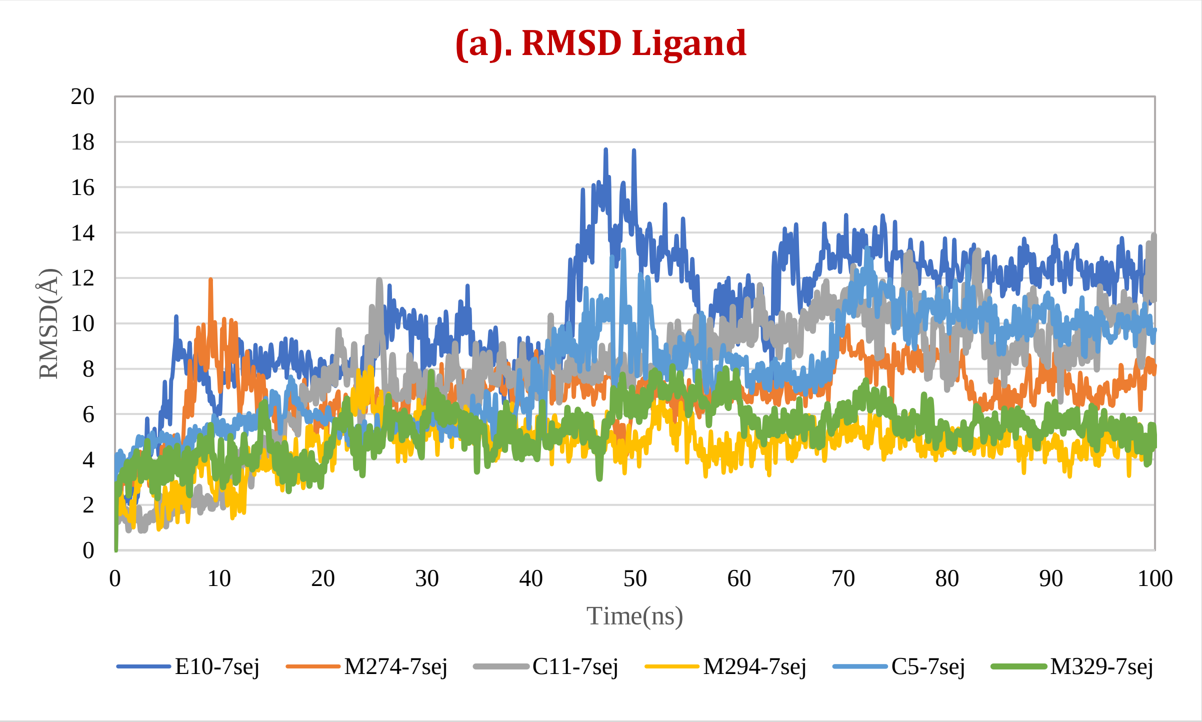

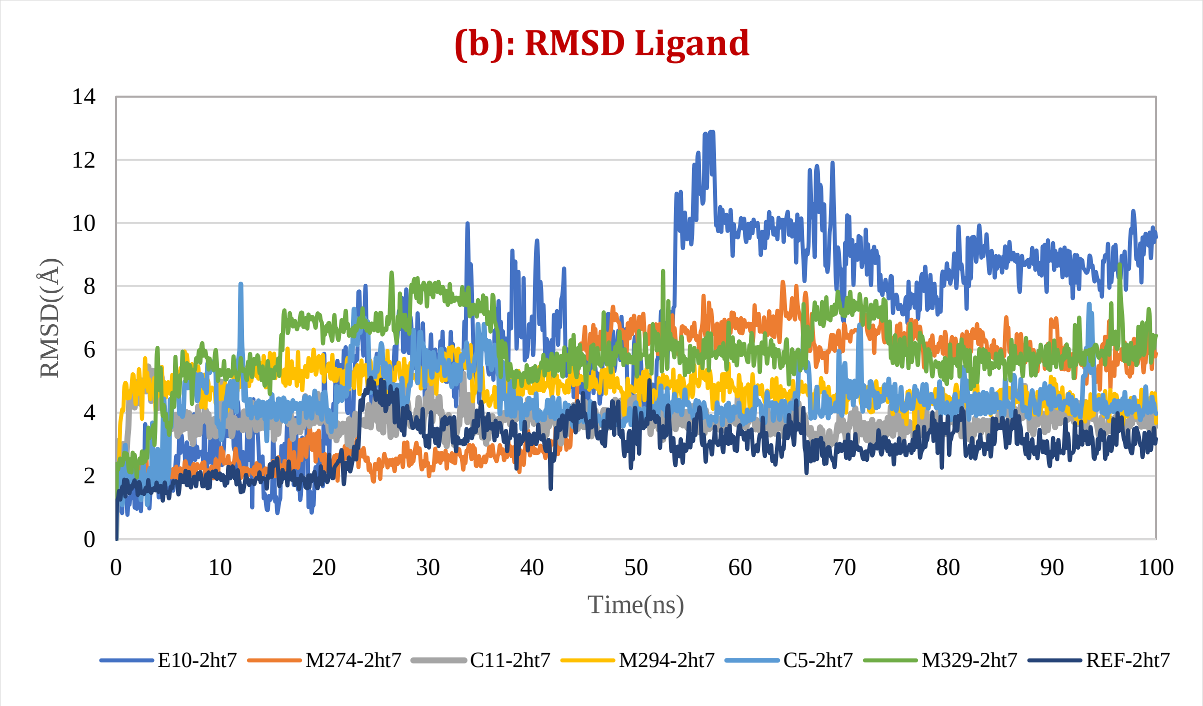

Figure 4. **(a)**: The RMSD profile of the ligands in complex with 7sej during the simulation. **(b)**: The RMSD profile of the ligands in complex with 2ht7 during the simulation.

3.3.2 RMSF profiles

Figure 5 shows the RMSF of the systems studied as a function of the simulation time. Most residues of protein 7sej fluctuated between 1 and 4 nm according to the RMSF analysis results. This indicates that all proteins in each simulated system maintained an overall stable structure. A marked flexibility peak was observed for amino acids 160 to 190 in all complexes, demonstrating the presence of a highly mobile loop. The amplitude of fluctuations in the C11-F and M294-7sej complexes was lower than that of the REF-7sej complex, suggesting a significant stabilizing effect due to ligand binding. Furthermore, the REF-7sej complex exhibited significant fluctuations around amino acid 300, fluctuations that disappeared in the complex systems, indicating stiffening induced by ligand binding. Additionally, fluctuations in the 380-420 region of the M274-7sej complex were significantly greater than those of the REF-7sej complex, suggesting a potential local conformational change or weak stabilizing interactions. In general, ligand binding induces a reduction in residue movement relative to the reference structure. C11-7sej and M294-7sej ligands produced greater stabilizing effects on protein dynamics than all other complexes.

The 2ht7 protein is relatively rigid and exhibits very low intrinsic flexibility, as indicated by its consistently low RMSF values across all residues. This low fluctuation is likely related to the maintenance of a stable tertiary structure in 2ht7 throughout the simulation, as well as its

very low local mobility. The relative rigidity of the overall structure may allow the protein to maintain its functional conformation in physiological environments, which is important for interactions with ligands and its ability to bind them. The limited mobility at the residue level suggests that the active and binding sites of 2ht7 are largely conserved, thus providing a structural context favorable to ligand interaction.

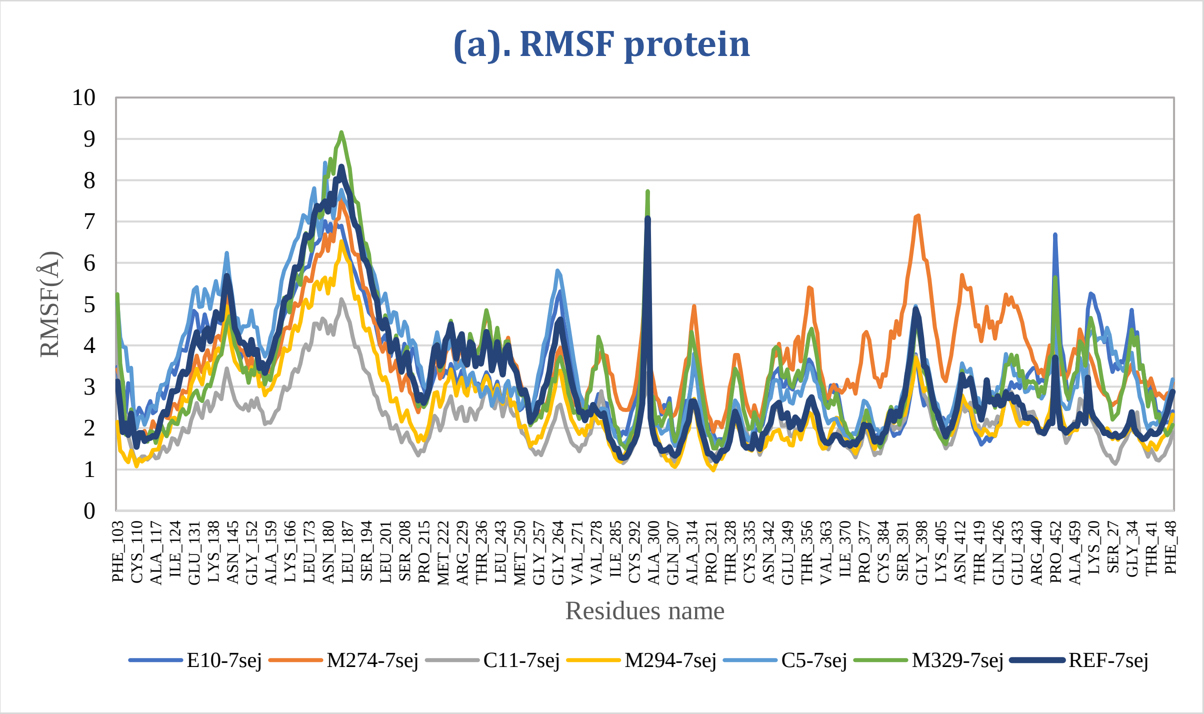

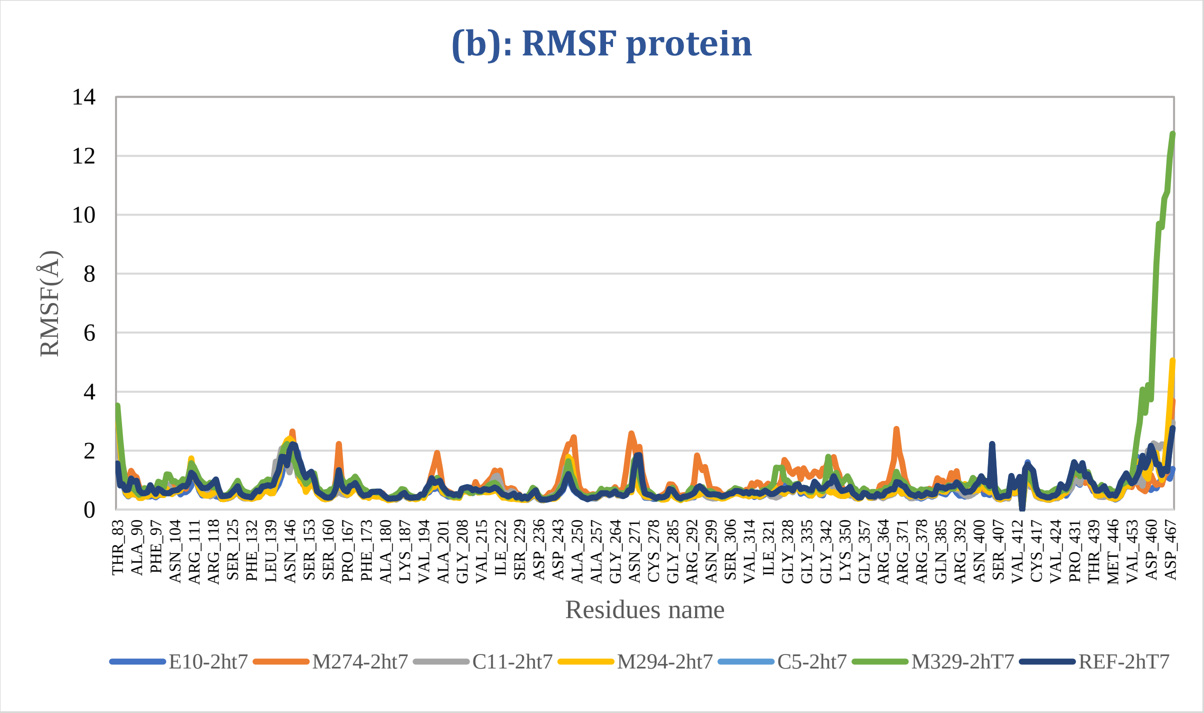

Figure 5. **(a)**: The RMSF profile of the protein 7sej in complex with the ligands studied. **(b)**: The RMSF profile of the protein 2ht7 in complex with the ligands studied

3.3.3 Protein–Ligand Contact Analysis

The results of the protein-ligand contact histogram and the chronology of interactions for each complex studied with the different viral targets NA (2ht7) and F (7sej) are presented in **Table 6(a,b)** as a function of simulation time. Analysis of the interactions in the M294–2ht7 complex revealed several key residues involved in binding, among which GLU119, ARG152, TRP178, and GLU227 showed dominant, persistent, and stable interactions over time, with significant contributions from hydrogen bonds, ionic interactions, and hydrophobic interactions. These interactions contribute to the stabilization of ligand M294 in the active site of the 2ht7 structure, allowing it to remain firmly anchored during the simulation, which is consistent with the low RMSD values observed for the ligand. For the C11–2ht7 complex, ligand stabilization was maintained through major interactions with several key residues, notably ARG156, GLN136, ARG118, and GLU119, forming hydrogen bonds, ionic interactions and hydrophobic interactions with the 2ht7 protein, thus indicating their crucial role in ligand binding within the active site of the protein. These results confirm the structural stability observed in previous analyses. Ligand M274 establishes a diverse network of interactions with protein 2ht7 through several hydrogen bonds, ionic interactions, and water bridges, mainly with residues GLU119, ASP151, GLU227, ARG152, GLU277, and ARG292. These interactions contribute to the stabilization of ligand M274 within the binding site of protein 2ht7. However, they appear to be intermittent and moderately dynamic over time, suggesting moderate stability.

In addition, ligand M329 forms a complex with 2ht7 through hydrogen bonds, several water bridges, and hydrophobic interactions, involving several residues such as ARG118, GLY147, ARG371, and TRP403. Among these interactions, the ARG118 residue appears to be the most frequent with a significant interaction fraction, playing a key role in the stabilization and binding of M329 within 2ht7. However, according to the interaction timeline of this complex, the majority of interactions appear to be alternative and time-limited, giving the binding of ligand M329 a more dynamic character compared to the other ligands studied.

On the other hand, analysis of the interactions of M294–7sej complex reveals stability similar to that observed with the NA(2ht7) receptor, characterized by persistent interactions with a high fraction of interactions involving several key residues. These interactions include hydrogen bonds, water bridges, and hydrophobic interactions, particularly with residues ASP325, ASP336, and LYS254. Additional moderate interactions also contribute to the

stabilization of the ligand in the binding site of the 7sej protein, such as those involving PHE256, VAL118, and ALA117, throughout the simulation. All of these interactions contribute to the solid maintenance of the ligand's anchorage in the active site of the 7sej protein. Regarding the C11–7sej complex, analysis of the interactions shows that the C11 ligand interacts mainly with the LYS386 residue, forming a persistent hydrogen bond interaction. Other interactions, notably ionic and water bridges, are also observed with residues GLY294 and THR114. Overall, thanks to these interactions, and particularly the one with residue LYS386, the C11 ligand remains firmly anchored in the active site of the 7sej protein.

Ligand M274 interacts with protein 7sej via a diverse network of interactions including hydrogen bonds, water bridges, and hydrophobic interactions, primarily with residues ASP454, CYS463, PHE446, LYS295, among others. Some of these interactions are persistent throughout much of the simulation, while others appear more dynamically over time. Overall, these interactions contribute to the maintenance of ligand M274 within the binding site of protein 7sej, suggesting moderate binding stability. Furthermore, ligand M329 maintains its stability with the 7sej protein mainly through hydrogen bonds, water bridges, and hydrophobic interactions, involving key residues such as LYS254 and ASP336, which form persistent and relatively continuous interactions over time. In addition, other interactions appear intermittently, via residues PHE256, ASP325, LYS324, and VAL118, indicating a moderately stable binding mode.

In contrast, analysis of the interactions of ligand E10 with the two viral targets 2ht7 and 7sej also reveals several interactions, including hydrogen bonds, water bridges, and hydrophobic interactions. However, these interactions are not continuous over time, indicating greater mobility of ligand E10 within the binding site of both receptors. This observation is consistent with the higher RMSD values of the ligand observed during the simulation.

In general, based on the results obtained, ligands M294 and C11 appear to be the most promising candidates, exhibiting persistent and more stable interactions with both NA(2ht7) and F(7sej) receptors over time. In addition, ligands C5 and M274 showed specific stability toward NA receptor, while ligand M329 showed particular stability toward F receptor.

Table 6. Contact diagrams and Interaction timelines of Protein-Ligand Complexes for selected ligands with **(a)** NA (PDB ID :2ht7) and **(b)** F (PDB ID: 7sej)

**(a)**	**Protein-ligand contact diagram**	**Protein-ligand interaction timeline**
**E10- 2ht7 complex**	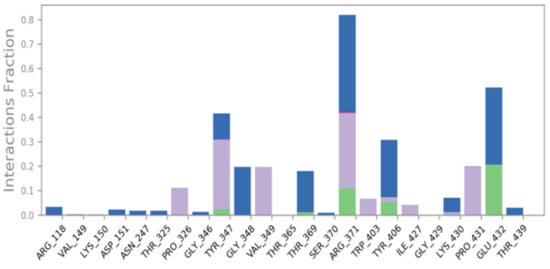	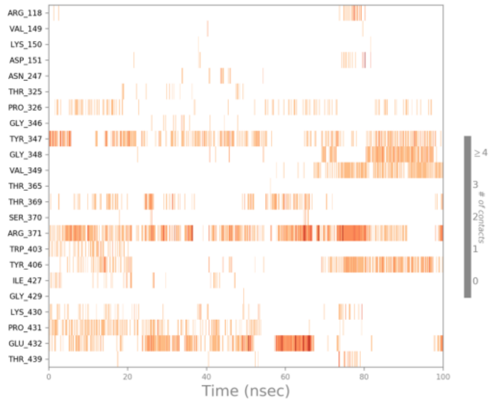
**M294- 2ht7 complex**	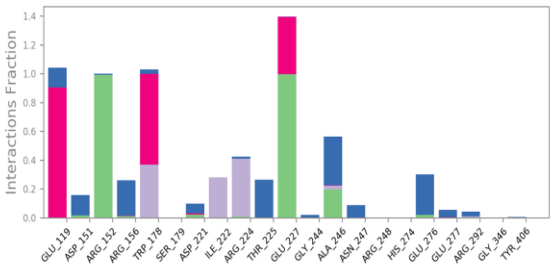	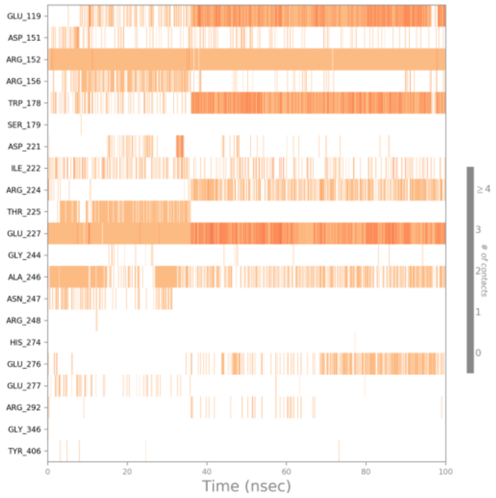

M329 - 2ht7 complex

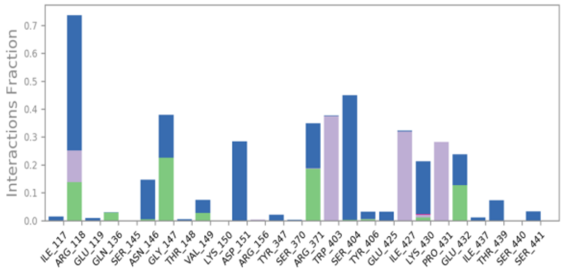

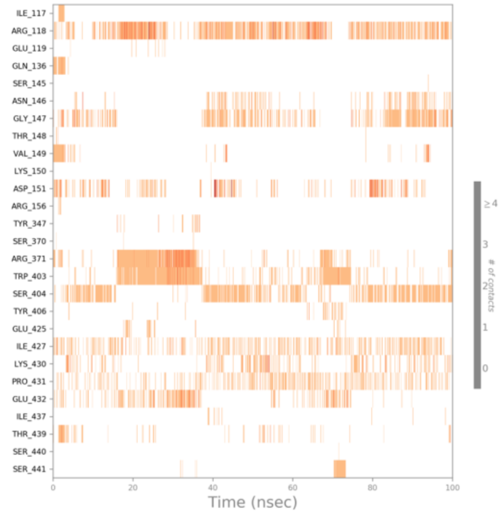

M274 - 2ht7 complex

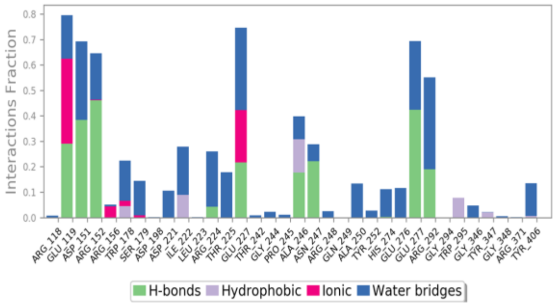

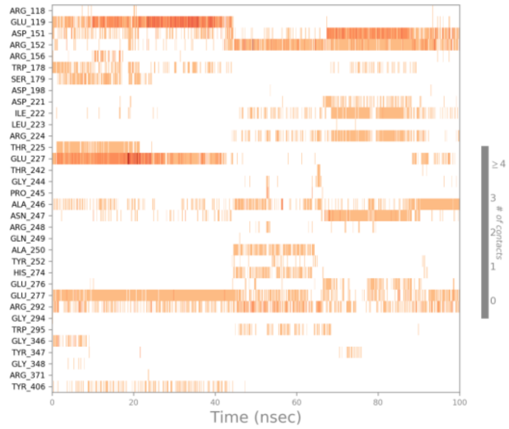

C11 - 2ht7 complex

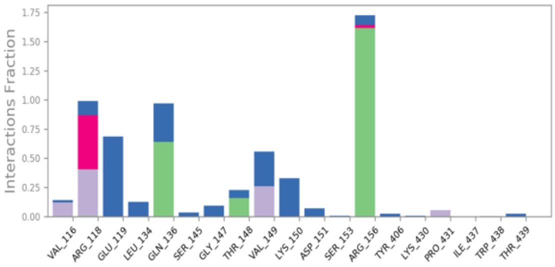

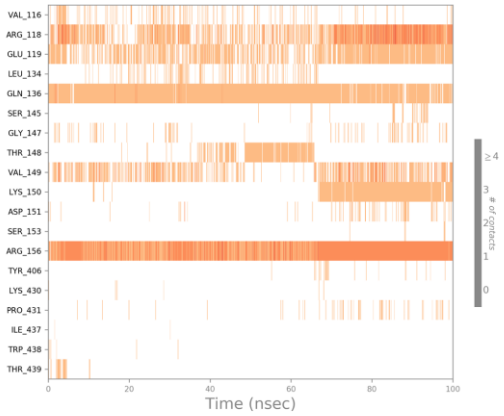

C5 - 2ht7 complex

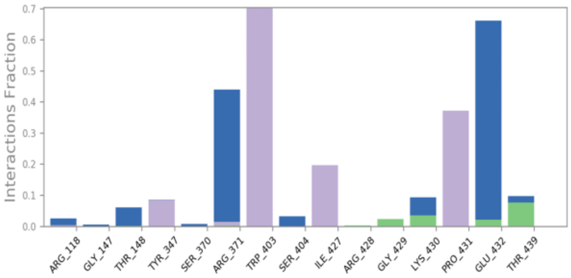

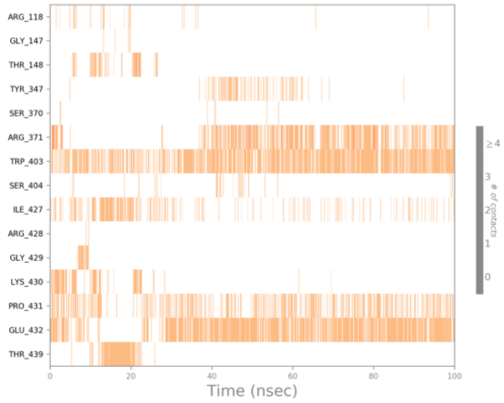

(b) **Protein-ligand contact diagram** **Protein-ligand interaction timeline**

E10- 7sej complex

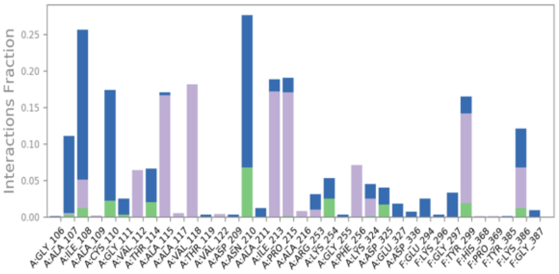

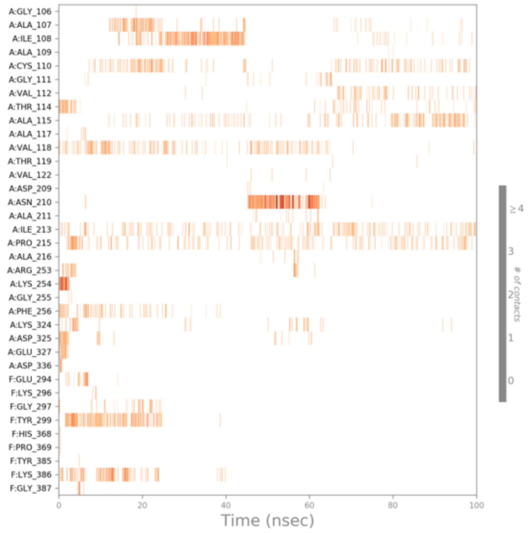

M294- 7sej complex

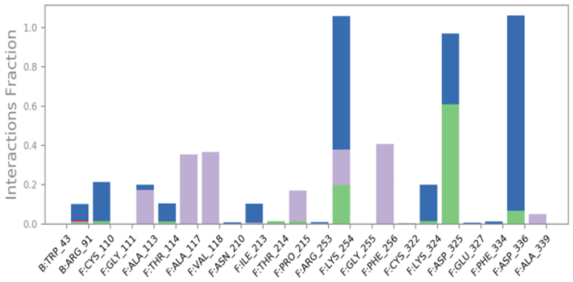

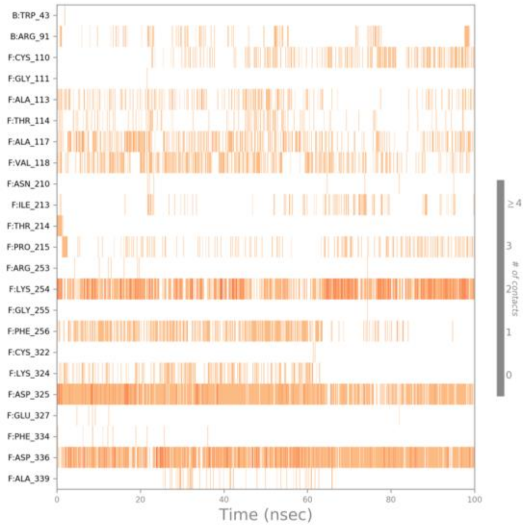

M329 -7sej complex

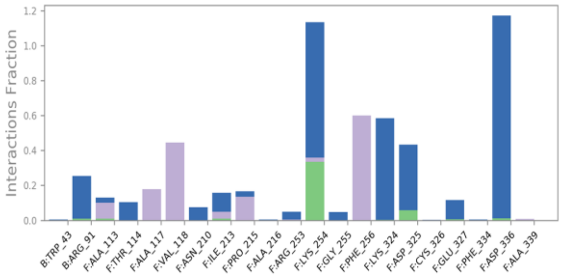

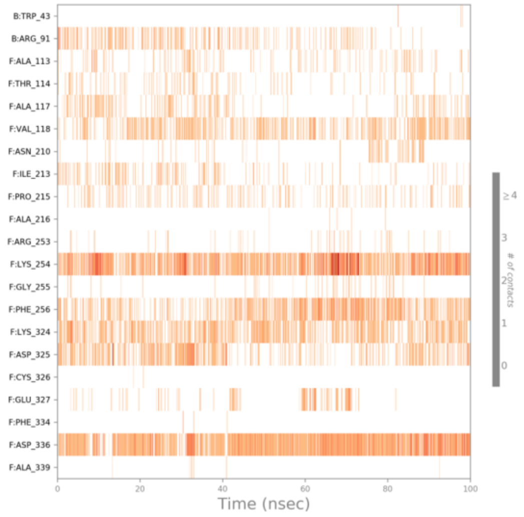

M274 - 7sej complex

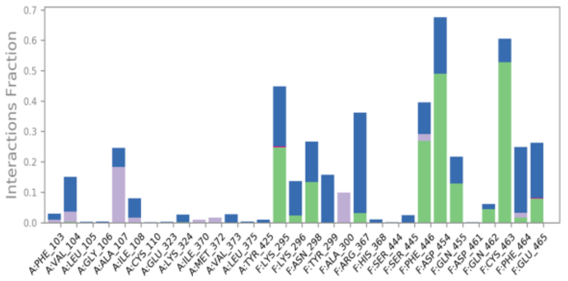

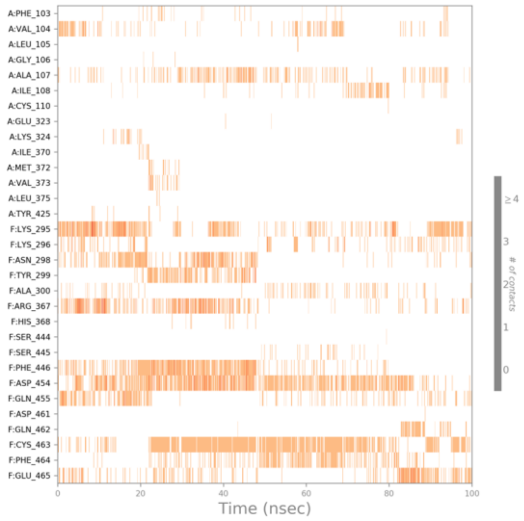

C11 - 7sej complex

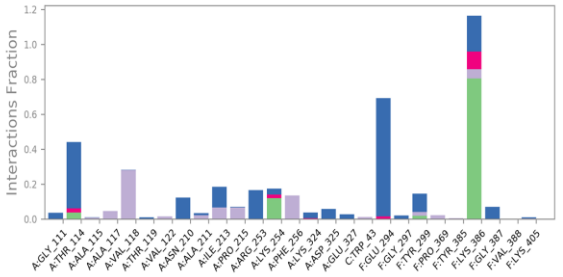

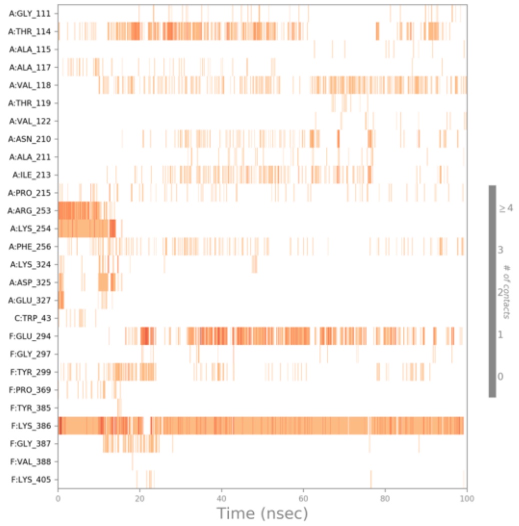

C5 - 7sej complex

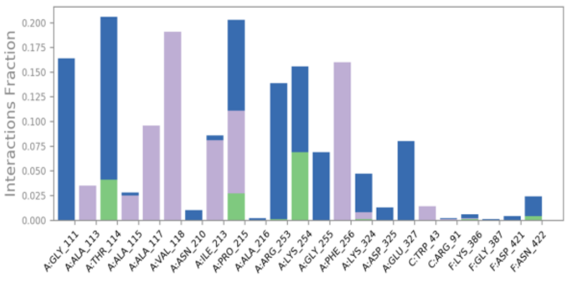

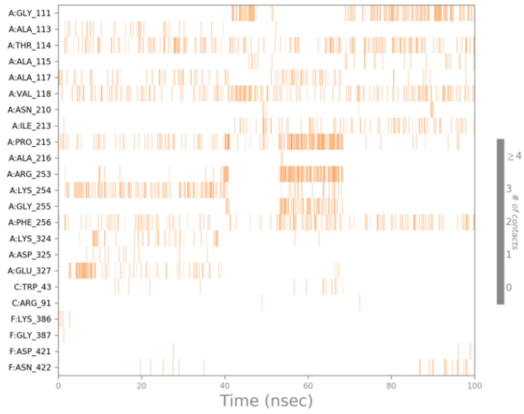

Conclusion:

This study explored the broad-spectrum antiviral potential of antiviral phytoconstituents, which have been the subject of several previous studies, targeting two proteins involved in respiratory infections: neuraminidase (NA) from influenza A virus and fusion protein (F) from HMPV. A molecular modeling approach was adopted as the main method, combining molecular docking, ADMET property prediction, and molecular dynamics simulation. The results of molecular docking revealed strong multi-target antiviral potential for a set of 37 phytoconstituents, with binding affinities higher than to those of the reference molecules, oseltamivir and ribavirin. After a thorough evaluation of their ADMET drug profile, including bioavailability analysis and analysis of pharmacokinetic and toxicological properties, phytoconstituents numbered E10, M294, M329, C11, C5, and M274 were selected as candidate compounds due to their good bioavailability, favorable ADME profile, and absence of major violations concerning their toxicological properties. Molecular dynamics (MD) simulations demonstrated the stability of compounds M294 (apigenin, a Moroccan medicinal plant derived from *Petroselinum crispum* L., Apiaceae) and C11 (tetrahydrocannabinolic acid, extracted from *Cannabis sativa* L., Cannabaceae) with the two viral targets NA and F over the duration of the simulation. In addition, the compounds C5 (tetrahydrocannabivarin, extracted from *Cannabis sativa* L., Cannabaceae) and M274 (Sidisterone, a Moroccan medicinal plant derived from *Ajuga iva* L., Lamiaceae) showed particular stability toward the NA of influenza A virus, forming stable bonds during 100 ns of simulation, while compound M329 (Jaceosidin, a Moroccan medicinal plant derived from *Mentha suaveolens* L., Lamiaceae) showed specific stability against the F protein of HMPV. Overall, this computational study has identified compounds M294 and C11 as natural broad-spectrum antiviral candidates, targeting both influenza A NA and HMPV-F protein. However, these results require further experimental validation, both in vivo and in vitro.

References:

1. Iuliano, A. D., Roguski, K. M., Chang, H. H., Muscatello, D. J., Palekar, R., & Tempia, S. (2018). Estimates of global seasonal influenza-associated respiratory mortality: A modelling study. *The Lancet, 391*(10127), 1285–1300. https://doi.org/10.1016/S0140-6736(17)33293-2
2. Steinberg, R., Marty, V., Korten, I., Aebi, C., Latzin, P., & Agyeman, P. K. (2024). Epidemiology and clinical characteristics of human metapneumovirus infections in hospitalized children in two consecutive postpandemic years. *The Pediatric Infectious Disease Journal*, *43*(2), 141–144. https://doi.org/10.1097/INF.0000000000004221
3. Mounadi, N., Nour, H., Abass, K. S., El Kouali, M., & Chtita, S. (2025). Exploring drug repurposing for influenza A (H3N2) virus: A computational approach to identifying commercialized drugs targeting hemagglutinin, neuraminidase, and nucleoprotein. *Journal of Chemical Information and Modeling*, *65*(21), 11837–11848. https://doi.org/10.1021/acs.jcim.5c00992
4. Mahal, A., Duan, M., Zinad, D. S., Mohapatra, R. K., Obaidullah, A. J., Wei, X., & Zhu, Q. (2021). Recent progress in chemical approaches for the development of novel neuraminidase inhibitors. *RSC Advances*, *11*(3), 1804–1840. https://doi.org/10.1039/D0RA07283D
5. Hossain, M. M., Apu, M. J. H., Bin Abdul Aziz, M. F., Tanjil, M. T. R., Das, L. C., Kar, A., Evamoni, F. Z., & Morshed, M. M. (2025). Exploring Dolichos lablab compounds as potential inhibitors for fusion (F) protein of human metapneumovirus (HMPV): A systematic computational approach. *PLOS ONE, 20*(9), e0332170. https://doi.org/10.1371/journal.pone.0332170
6. Mounadi, N., Nour, H., Daoui, O., Elkhattabi, S., Errougui, A., Talbi, M., El Kouali, M., & Chtita, S. (2024). Computational studies of cannabis derivatives as potential inhibitors of SARS-CoV-2 Mpro. *Chemistry Africa, 5,* 2569–2580. https://doi.org/10.1007/s42250-024-00914-5
7. Nour, H., Yamari, I., Abchir, O., Mounadi, N., Samadi, A., Belaidi, S., & Chtita, S. (2025). Exploring Cannabis sativa L. for anti-Alzheimer potential: An extensive computational study including molecular docking, molecular dynamics, and ADMET assessments. *Medicinal Chemistry, 21*(5), 367–384. https://doi.org/10.2174/0115734064318657240822064240

8. Abchir, O., Daoui, O., Nour, H., Yamari, I., Elkhattabi, S., Errougui, A., & Chtita, S. (2023). Exploration of cannabis constituents as potential candidates against diabetes mellitus using molecular docking, dynamics simulations, and ADMET investigations. *Scientific African, 21*, e01745. https://doi.org/10.1016/j.sciaf.2023.e01745

9. Daoui, O., Mali, S. N., Elkhattabi, K., Elkhattabi, S., & Chtita, S. (2023). Repositioning cannabinoids and terpenes as novel EGFR-TKIs candidates for targeted therapy against cancer: A virtual screening model using CADD and biophysical simulations. *Heliyon, 9*(4), e15545. https://doi.org/10.1016/j.heliyon.2023.e15545

10. Bouamri, L. E., Laaouina, S., Lakrim, I., Nour, H., Yamari, I., Samadi, A., Bouachrine, M., & Chtita, S. (2026). Integrated computational investigation of Cannabis sativa phytoconstituents as putative multi-target inhibitors in skin cancer: A molecular docking, dynamics, and ADMET profiling study. *Pharmaceuticals, 19*(2), 315. https://doi.org/10.3390/ph19020315

11. Nour, H., Abdou, A., Belaidi, S., Jamal, J., Elmakssoudi, A., Dakir, M., & Chtita, S. (2022). Discovery of promising cholinesterase inhibitors for Alzheimer's disease treatment through DFT, docking, and molecular dynamics studies of eugenol derivatives. *Journal of the Chinese Chemical Society, 69*(9), 1534–1551. https://doi.org/10.1002/jccs.202200195

12. Mounadi, N., Nour, H., Errougui, A., Talbi, M., ElKouali, M., & Chtita, S. (2024). Discovery of eugenol-derived drug candidates for the treatment of COVID-19 by applying molecular docking, molecular dynamics, and pharmacokinetic analysis. *Physical Chemistry Research, 12*(2), 289–303.

13. Mounadi, N., Nour, H., Bouribab, A., Khedraoui, M., Kouali, M. E., Samadi, A., & Chtita, S. (2025). In silico analysis of Moroccan medicinal plant compounds as potential inhibitors of influenza A (H3N2). *Scientific African, 30***,** e02969. https://doi.org/10.1016/j.sciaf.2025.e02969

14. Protein Data Bank. (2026). Crystal structure of neuraminidase (PDB ID: 2HT7). Retrieved January 3, 2026, from https://www.rcsb.org/structure/2HT7

15. Protein Data Bank. (2026). Fusion protein of human metapneumovirus (PDB ID: 7SEJ). Retrieved January 3, 2026, from https://www.rcsb.org/structure/7SEJ

16. Trott, O., & Olson, A. J. (2010). AutoDock Vina: Improving the speed and accuracy of docking with a new scoring function, efficient optimization, and multithreading. *Journal of Computational Chemistry, 31*(2), 455–461. https://doi.org/10.1002/jcc.21334

17. BIOVIA. (2021). Discovery Studio [Computer software]. Dassault Systèmes.

18. Daina, A., Michielin, O., & Zoete, V. (2017). SwissADME: A free web tool to evaluate pharmacokinetics, drug-likeness and medicinal chemistry friendliness of small molecules. *Scientific Reports*, *7*, 42717. https://doi.org/10.1038/srep42717

19. Banerjee, P., Eckert, A. O., Schrey, A. K., & Preissner, R. (2018). ProTox-II: A webserver for the prediction of toxicity of chemicals. *Nucleic Acids Research*, *46*(W1), W257–W263. https://doi.org/10.1093/nar/gky318

20. Lipinski, C. A., Lombardo, F., Dominy, B. W., & Feeney, P. J. (2001). Experimental and computational approaches to estimate solubility and permeability in drug discovery and development settings. *Advanced Drug Delivery Reviews*, *46*(1–3), 3–26. https://doi.org/10.1016/S0169-409X(00)00129-0

21. Veber, D. F., Johnson, S. R., Cheng, H. Y., Smith, B. R., Ward, K. W., & Kapple, K. D. (2002). Molecular properties that influence the oral bioavailability of drug candidates. *Journal of Medicinal Chemistry*, *45*(12), 2615–2623. https://doi.org/10.1021/jm020017n

22. Schrödinger, LLC. (2020). Desmond [Computer software]. https://www.schrodinger.com/platform/products/desmond

23. Roos, K., Wu, C., Damm, W., Reboul, M., Stevenson, J. M., Lu, C., et al. (2019). OPLS3e: Extending force field coverage for drug-like small molecules. *Journal of Chemical Theory and Computation*, *15*(5), 1863–1874. https://doi.org/10.1021/acs.jctc.8b01026

24. Mark, P., & Nilsson, L. (2001). Structure and dynamics of the TIP3P, SPC, and SPC/E water models at 298 K. *Journal of Physical Chemistry A*, *105*(46), 9954–9960. https://doi.org/10.1021/jp003020w

25. Parrinello, M., & Rahman, A. (1981). Polymorphic transitions in single crystals: A new molecular dynamics method. *Journal of Applied Physics*, *52*(12), 7182–7190. https://doi.org/10.1063/1.328693

Click here to access/download
Supporting Information
SM.docx
