# Supplementary material for: Repositioning antiviral phytoconstituents as broad-spectrum inhibitors of influenza A neuraminidase and human metapneumovirus fusion protein
Source: PLoS One. 2026 Sep 2;21(9):e0348517. doi: 10.1371/journal.pone.0348517 (PMC13537546; doi:10.1371/journal.pone.0348517)
Supplement: Supplementary file 3 [file pone.0348517.s001.docx]

**Table S1.** Names of the studied phytoconstituents, their sources, and chemical structures

| Source (Plant / Origin) | Compound | No. | Structures |
| --- | --- | --- | --- |
| *Cannabis Saliva* L.  (Cannabaceae) | Cannabidiolic Acid | C1 |  |
|  | Cannabigerolic Acid | C2 |  |
|  | Cannabiphorol | C3 |  |
|  | Cannabidiol | C4 |  |
|  | Tetrahydrocannabivarin | C5 |  |
|  | Cannabinol | C6 |  |
|  | Δ9-Tetrahydrocannabinol | C7 |  |
|  | Δ9-Tetrahydrocannabinol | C8 |  |
|  | Cannabicyclol | C9 |  |
|  | Cannabichromene | C10 |  |
|  | Tetrahydrocannabinolic Acid | C11 |  |
|  | Cannabichromenic Acid | C12 |  |
|  | Bulnesol | C13 |  |
|  | Camphene | C14 |  |
|  | d-Limonene | C15 |  |
|  | Eudesma | C16 |  |
|  | Farnesene | C17 |  |
|  | Geraniol | C18 |  |
|  | Isopulegol | C19 |  |
|  | Linalool | C20 |  |
|  | Nerolidol | C21 |  |
|  | Ocimene | C22 |  |
|  | p-Cymene | C23 |  |
|  | Terpinolene | C24 |  |
|  | α-Humulene | C25 |  |
|  | α-Pinene | C26 |  |
|  | α-Terpinene | C27 |  |
|  | β-Caryophyllene | C28 |  |
|  | β-Myrcene | C29 |  |
|  | β-Pinene | C30 |  |
|  | γ-3-Carene | C31 |  |
|  | γ-Terpinene | C32 |  |
| Eugenol derivatives | Eugenol | E1 |  |
|  | Acetyleugenol | E2 |  |
|  | 4-allyl-2-methoxyphenyl benzoate | E3 |  |
|  | 4-(5-allyl-3-methoxy-2-phenooxybenzyl)morpholine | E4 |  |
|  | 4-allyl-2-methoxy-6(morpholinomethyl)phenol | E5 |  |
|  | 4-(5-allyl-2,3-dimethoxybenzyl)morphol | E6 |  |
|  | 4-(5-allyl-2-(4-chlorophenoxy)-3-methoxybenzyl)morpholine | E7 |  |
|  | (2R, 4R, 5R)-2-(acetoxymethyl)-6-(4-allyl-2-methoxyphenoxy)- tetrahydro-2H-pyran-3,4,5triacetate | E8 |  |
|  | (3R, 4R, 6R)-2-(4-allyl-2-methoxyphenoxy)-6-(hydroxymethyl)tetrahydro-2H-pyran-3,4,5-triol | E9 |  |
|  | (4aR,7R,8S,8aS)-6-(4-allyl-2-methoxyphenoxy)-2-phenylhexanhydropyrano[3,2-d][1,3]dioxine-7,8-diol | E10 |  |
|  | 4-allyl-2-methoxyphenyl 2-fluorobenzoate | E11 |  |
|  | 4-allyl-2-methoxyphenyl 3-bromobenzoate | E12 |  |
|  | 4-allyl-2-methoxyphenyl 3-chlorobenzoate | E13 |  |
|  | 4-allyl-2-methoxyphenyl-4-methoxybenzoate | E14 |  |
|  | 4-allyl-2-methoxyphenyl 4-methylbenzoate | E15 |  |
|  | 4-allyl-2-methoxyphenyl-2-phenylacetate | E16 |  |
|  | 5-allyl-7-methoxy-2-phenylbenzo[*d*]-oxazole | E17 |  |
|  | 5-allyl-7-methoxy-2-(4-methoxyphenyl)benzo[*d*]-oxazole | E18 |  |
|  | Iso-eugenol | E19 |  |
|  | Chavicol | E20 |  |
|  | Chavibetol | E21 |  |
|  | Estragol | E22 |  |
|  | Anethol | E23 |  |
|  | Safrole | E24 |  |
|  | Isosafrole | E25 |  |
|  | Myristicine | E26 |  |
|  | Elemicine | E27 |  |
|  | Methyleugenol | E28 |  |
|  | 4-(2-hydroxypropyl)-2-methoxyphemol | E29 |  |
|  | 3-(4-hydroxy-3-methoxyphenyl) propane-1,2-diol | E30 |  |
|  | 4-((2,2-dimethyl-1,3-dioxolan-4-yl)methyl)-2-methoxyphenol | E31 |  |
|  | 4-(2-hydroxy-3-(2-hydroxyphenoxy)propyl)-2-methoxyphenol | E32 |  |
|  | 4-(2-hydroxy-3-(3-hydroxyphenoxy)propyl)-2-methoxyphenol | E33 |  |
|  | 4-(2-hydroxy-3-(4-hydroxyphenoxy)propyl)-2-methoxyphenol | E34 |  |
|  | 5-(4-hydroxy-3-methoxybenzyl)dihydrofuran-2(3H)-one | E35 |  |
|  | 4-(4-hydroxy-3-methoxyphenyl)-butanal | E36 |  |
|  | 3-(4-hydroxy-3-methoxyphenyl)-2-methylpropanal | E37 |  |
|  | 2-methoxy-4-(3-(phenylthio)propyl) phenol | E38 |  |
|  | 2-((4-allyl-2-methoxyphenoxy)methyl) oxirane) | E39 |  |
|  | 1-(4-allyl-2-methoxyphenoxy)-3-(diethylamino)propan-2-ol | E40 |  |
|  | 4-allyl-2-methoxyphenyl phenylmethanesulfonate | E41 |  |
|  | 2-(4-allyl-2-methoxyphenoxy)acetic acid | E42 |  |
|  | methyl 2-(4-allyl-2-methoxyphenoxy)acetate | E43 |  |
|  | 2-(4-allyl-2-methoxyphenoxy)-N,N-bis(2-hydroxyethyl)acetamide | E44 |  |
|  | 4-allyl-2-methoxy-6-nitrophenol | E45 |  |
|  | 5-allyl-3-nitrobenzene-1,2-diol | E46 |  |
|  | 4-allyl-2-methoxy-3-nitrophenol | E47 |  |
|  | 4-allyl-4-hydroperoxy-2-methoxycyclohexa-2,5-dien-1-one | E48 |  |
|  | 4-allyl-2-amino-6-methoxyphenol | E49 |  |
|  | (6-(4-hydroxy-3-methoxybenzyl)-3-methyl-2,4-diazabicyclo[3.2.0]hept-3-en-2-yl)(2R)-3-(2-hydroxynaphthalen-1-yl)oxiran-2-yl)methanone | E50 |  |
|  | 5-allyl-7-methoxybenzo[d]oxazole-2(*3H*)-thione | E51 |  |
|  | 6-allyl-8-methoxy-3-methyl-3,4-dihydro-2H-benzo[e][1,3]oxazine | E52 |  |
|  | 6-allyl-3-(furan-2-ylmethyl)-8-methoxy-3,4-dihydro-2H-benzo[e][1,3]oxazine | E53 |  |
|  | 2,2’-((5,5’-diallyl-3,3’-dimethoxy-[1,1’biphenyl]-2,2’-diyl)bis(oxy)bis(ethan-1-ol) | E54 |  |
|  | 2,2'-((5,5'-di(1λ^2^,2 λ^3^,3 λ^2^-allyl)-3,3'-di( λ^1^-methoxy)-4 λ^3^,4' λ^3^,6λ^3^,6'λ^3^-[1,1'-bi(cyclohexane)]-1,1',3,3',5,5'-hexaene-2,2'-diyl)bis(oxy))bis(1 λ^2^,2 λ^2^-ethan-1-ol) | E55 |  |
|  | 2-[(2-methoxy-4-prop-2-enylphenoxy)methyl]-3,5,6-trimethylpyrazine | E56 |  |
|  | 7-chloro-4-(2-methoxy-4-prop-2-enylphenoxy)quinoline | E57 |  |
|  | 2-((3-( λ^1^-methyl)-1 λ^2^,2 λ^3^,4 λ^1^-but-2-en-1-yl)oxy)-6-(3-(4-((3-( λ^1^-methyl)-1 λ^2^,2 λ^3^,4 λ^1^-but-2-en-1-yl)oxy)-3-(l1-methoxy)-2 λ^3^,5 λ^3^,6 λ^3^-phenyl)-1 λ^2^,2 λ^3^,3 λ^3^-allyl)-3-( λ^1^-methoxy)-4 λ^3^,5 λ^3^-phenol | E58 |  |
|  | 2-(4-(1 λ^2^,2 λ^3^,3 λ^2^-allyl)-2-( λ^1^-methoxy)-3 λ^3^,5 λ^3^,6 λ^3^-phenoxy)-1-(3-(4-chloro-2 λ^3^,3 λ^3^,5 λ^3^,6 λ^3^-phenyl)-5-(4-hydroxy-2 λ^3^,3 λ^3^,5 λ^3^,6 λ^3^-phenyl)-2 λ^2^,3 λ^3^,4 λ^2^,5 λ^3^-pyrazolidin-1-yl)-2 λ^2^-ethan-1-one | E59 |  |
| *Anacyclus pyrethrum* L.  (Astraceae) | Dihydroxybenzoic acid | M110 |  |
| *Anabasis aretioides* L. (Amaranthaceae) | Hesperidin | M17 |  |
| *Ajuga iva* L.  (Lamiaceae) | Apigenin 7-O-(6″-malonyl-apiosyl-glucoside) | M293 |  |
| *Mentha suaveolens* L.  (Lamiaceae) | Luteolin-dihexoside | M319 |  |
| *Mentha suaveolens* L.  (Lamiaceae) | Apigenin-dihexoside | M322 |  |
| *Ajuga iva* L.  (Lamiaceae) | Apigenin-7-(2-O-apiosylglucoside) | M292 |  |
| *Mentha suaveolens* L.  (Lamiaceae) | Salvianolic acid B | M324 |  |
| *Mentha suaveolens* L.  (Lamiaceae) | Salvianolic acid B/E | M378 |  |
| *Ajuga iva* L.  (Lamiaceae) | 24-hydroxycyasterone | M269 |  |
| *Ajuga iva* L.  (Lamiaceae) | Ajugasterone D | M285 |  |
| *Anabasis aretioides* L.  (Amaranthaceae) | Naringin | M16 |  |
| *Mentha suaveolens* L.  (Lamiaceae) | Luteolin-hexoside | M320 |  |
| *Mentha suaveolens* L.  (Lamiaceae) | Luteolin-glucuronide | M321 |  |
| *Ajuga iva* L.  (Lamiaceae) | Ferulic acid 4-O-glucoside | M286 |  |
| *Ajuga iva* L.  (Lamiaceae) | Sidisterone | M274 |  |
| *Anacyclus pyrethrum* L.  (Astraceae) | Isochlorogenic acid b | M109 |  |
| *Anacyclus pyrethrum* L.  (Astraceae) | Isochlorogenic acid C | M111 |  |
| *Ajuga iva* L.  (Lamiaceae) | 22-Dehydrocyasterone 2-glucoside | M270 |  |
| *Ajuga iva* L.  (Lamiaceae) | Abutasterone | M267 |  |
| *Ajuga iva* L.  (Lamiaceae) | Cyasterone | M268 |  |
| *Ajuga iva* L.  (Lamiaceae) | Quercetin-3-O-pentosyl-pentoside | M295 |  |
| *Anacyclus pyrethrum* L.  (Astraceae) | Oleuropein | M148 |  |
| *Anabasis aretioides* L.  (Amaranthaceae) | Luteolin | M22 |  |
| *Ajuga iva* L.  (Lamiaceae) | Myricetin | M276 |  |
| *Mentha suaveolens* L.  (Lamiaceae) | Jaceosidin | M329 |  |
| *Petroselinum crispum* L.  ([Apiacee](https://www.google.com/search?sca_esv=5480f011bd3e2bf8&sxsrf=ACQVn0-LW2ZEc4gOEstzxKK0DIH_c1UPhw:1713709932155&q=Apiac%C3%A9es&stick=H4sIAAAAAAAAAONgVmLXz9U3yDEzf8RoxC3w8sc9YSnNSWtOXmNU5uIKzsgvd80rySypFBLlYoOyuKU4uWB6eBaxcjoWZCYmH16ZWgwAYr2zf0wAAAA&sa=X&ved=2ahUKEwiy54P-wtOFAxXlUqQEHSPRAHoQzIcDKAB6BAgWEAE)) | apigenin | M294 |  |
| *Artemisia ifranensis* L.  (Astraceae) | Cedr-8(15)-en-9- α -ol acetate | M259 |  |
| *Foeniculum vulgare* L.  ([Apiacee](https://www.google.com/search?sca_esv=5480f011bd3e2bf8&sxsrf=ACQVn0-LW2ZEc4gOEstzxKK0DIH_c1UPhw:1713709932155&q=Apiac%C3%A9es&stick=H4sIAAAAAAAAAONgVmLXz9U3yDEzf8RoxC3w8sc9YSnNSWtOXmNU5uIKzsgvd80rySypFBLlYoOyuKU4uWB6eBaxcjoWZCYmH16ZWgwAYr2zf0wAAAA&sa=X&ved=2ahUKEwiy54P-wtOFAxXlUqQEHSPRAHoQzIcDKAB6BAgWEAE)) | 1-Butanone, 2-chloro-3-methyl-1-[4-(1-methylethyl)phenyl]- | M102 |  |
| *Haloxylon scoparium* L.  (Amaranthaceae) | α-D-Glucopyranose pentaacetate | M80 |  |
| *Haloxylon scoparium* L.  (Amaranthaceae) | alpha-D-Glucopyranoside, 1,3,4,6-tetra-O-acetyl-beta-D-fructofuranosyl, 2,3,4,6-tetraacetate | M81 |  |
