# Supplementary material for: Repositioning antiviral phytoconstituents as broad-spectrum inhibitors of influenza A neuraminidase and human metapneumovirus fusion protein
Source: PLoS One. 2026 Sep 2;21(9):e0348517. doi: 10.1371/journal.pone.0348517 (PMC13537546; doi:10.1371/journal.pone.0348517)
Supplement: Supplementary file 4 [file pone.0348517.s002.docx]

**Table S2.** Molecular docking binding energies (BE) of the studied phytoconstituents targeting both NA (PDB ID: 2ht7) and HMPV-F (PDB ID: 7sej) proteins compared with reference compounds.

| Source (Plant / Origin) | Ligand | BE (kcal/mol)  (PDB ID: 2ht7) | BE (kcal/mol)  (PDB ID: 7sej) |
| --- | --- | --- | --- |
| *Cannabis Saliva* L.  (Cannabaceae) | C1 | -7.4 | -6.8 |
|  | C2 | -7.4 | -6.2 |
|  | C3 | -7.0 | -6.7 |
|  | C4 | -6.4 | -6.4 |
|  | C5 | -7.7 | -7.4 |
|  | C6 | -7.2 | -7.1 |
|  | C7 | -7.6 | -7.4 |
|  | C8 | -7.2 | -7.0 |
|  | C9 | -7.3 | -7.2 |
|  | C10 | -7.3 | -6.6 |
|  | C11 | -8.0 | -7.3 |
|  | C12 | -7.4 | -6.7 |
|  | C13 | -6.2 | -5.9 |
|  | C14 | -5.7 | -5.5 |
|  | C15 | -5.9 | -5.7 |
|  | C16 | -7.1 | -7.0 |
|  | C17 | -6.0 | -5.6 |
|  | C18 | -4.4 | -4.2 |
|  | C19 | -5.9 | -5.8 |
|  | C20 | -5.0 | -4.4 |
|  | C21 | -5.0 | -5.7 |
|  | C22 | -5.3 | -5.3 |
|  | C23 | -5.9 | -5.7 |
|  | C24 | -5.5 | -5.7 |
|  | C25 | -6.4 | -6.7 |
|  | C26 | -5.2 | -5.5 |
|  | C27 | -5.7 | -5.6 |
|  | C28 | -6.0 | -7.0 |
|  | C29 | -4.9 | -4.8 |
|  | C30 | -5.9 | -5.4 |
|  | C31 | -5.6 | -5.4 |
|  | C32 | -5.9 | -5.6 |
| *Eugenol derivatives* | E1 | -6.2 | -5.5 |
|  | E2 | -6.4 | -5.8 |
|  | E3 | -7.1 | -6.6 |
|  | E4 | -6.8 | -6.3 |
|  | E5 | -7.1 | -5.9 |
|  | E6 | -7.0 | -5.9 |
|  | E7 | -7.3 | -6.6 |
|  | E8 | -7.3 | -6.3 |
|  | E9 | -7.2 | -6.8 |
|  | E10 | -8.2 | -8.5 |
|  | E11 | -6.6 | -5.7 |
|  | E12 | -6.6 | -6.1 |
|  | E13 | -6.7 | -5.6 |
|  | E14 | -6.9 | -5.6 |
|  | E15 | -6.6 | -6.5 |
|  | E16 | -6.8 | -5.9 |
|  | E17 | -7.0 | -6.5 |
|  | E18 | -7.9 | -7.1 |
|  | E19 | -8.0 | -7.3 |
|  | E20 | -5.7 | -5.7 |
|  | E21 | -5.8 | -5.3 |
|  | E22 | -6.2 | -5.7 |
|  | E23 | -5.7 | -5.5 |
|  | E24 | -5.5 | -5.6 |
|  | E25 | -6.2 | -5.8 |
|  | E26 | -6.1 | -6.0 |
|  | E27 | -6.7 | -5.7 |
|  | E28 | -5.5 | -5.5 |
|  | E29 | -6.1 | -5.2 |
|  | E30 | -5.8 | -5.7 |
|  | E31 | -5.7 | -5.4 |
|  | E32 | -6.4 | -6.2 |
|  | E33 | -6.0 | -6.1 |
|  | E34 | -5.9 | -6.6 |
|  | E35 | -6.4 | -6.0 |
|  | E36 | -6.4 | -6.3 |
|  | E37 | -5.8 | -5.4 |
|  | E38 | -5.6 | -5.9 |
|  | E39 | -6.0 | -5.0 |
|  | E40 | -6.1 | -5.9 |
|  | E41 | -6.1 | -5.7 |
|  | E42 | -7.2 | -6.5 |
|  | E43 | -6.3 | -6.0 |
|  | E44 | -6.1 | -5.9 |
|  | E45 | -6.1 | -5.8 |
|  | E46 | -6.5 | -5.5 |
|  | E47 | -6.4 | -5.9 |
|  | E48 | -6.0 | -6.3 |
|  | E49 | -6.1 | -5.9 |
|  | E50 | -6.9 | -5.8 |
|  | E51 | -9.5 | -9.2 |
|  | E52 | -6.8 | -6.6 |
|  | E53 | -7.0 | -5.9 |
|  | E54 | -7.1 | -6.7 |
|  | E55 | -6.3 | -5.3 |
|  | E56 | -7.2 | -7.2 |
|  | E57 | -7.4 | -7.1 |
|  | E58 | -8.4 | -7.2 |
|  | E59 | -8.3 | -7.7 |
| *Marocain Medicinal Plants* | M110 | -10.4 | -9.1 |
|  | M17 | -10.0 | -8.2 |
|  | M293 | -10.0 | -9.1 |
|  | M319 | -9.1 | -8.0 |
|  | M322 | -9.1 | -8.1 |
|  | M292 | -9.0 | -8.1 |
|  | M324 | -9.0 | -8.7 |
|  | M378 | -9.0 | -8.4 |
|  | M269 | -8.9 | -8.5 |
|  | M285 | -8.9 | -8.1 |
|  | M16 | -8.7 | -8.0 |
|  | M320 | -8.7 | -8.7 |
|  | M321 | -8.7 | -8.5 |
|  | M286 | -8.6 | -8.2 |
|  | M274 | -8.5 | -8.2 |
|  | M109 | -8.4 | -7.3 |
|  | M111 | -8.4 | -8.1 |
|  | M270 | -8.4 | -9.1 |
|  | M267 | -8.1 | -8.6 |
|  | M268 | -8.1 | -8.6 |
|  | M295 | -8.1 | -8.3 |
|  | M148 | -8.1 | -7.0 |
|  | M22 | -8.0 | -7.3 |
|  | M276 | -8.0 | -7.2 |
|  | M329 | -8.0 | -7.3 |
|  | M294 | -7.4 | -8.0 |
|  | M259 | -7.4 | -6.6 |
|  | M102 | -7.4 | -6.7 |
|  | M80 | -7.4 | -6.2 |
|  | M81 | -7.4 | -7.4 |
| References |  | **-6.2** | **-5.8** |

**BE**: binding energies, **Reference^1:^** Oseltamivir, **Reference^2:^** Ribavirin
