# Supplementary material for: Repositioning antiviral phytoconstituents as broad-spectrum inhibitors of influenza A neuraminidase and human metapneumovirus fusion protein
Source: PLoS One. 2026 Sep 2;21(9):e0348517. doi: 10.1371/journal.pone.0348517 (PMC13537546; doi:10.1371/journal.pone.0348517)
Supplement: Supplementary file 5 [file pone.0348517.s003.docx]

**Table S3.** Physicochemical properties of the studied phytoconstituents based on to Lipinski’s and Veber’s rules in comparison with reference compounds.

| Ligands | Formula | Lipinski and Veber parameters | | | | | | Number of violations | |  |
| --- | --- | --- | --- | --- | --- | --- | --- | --- | --- | --- |
|  |  | **Mw** | **LogP** | **RB** | **HBA** | **HBD** | **PSA** | **Lipinski** | **Veber** | |
| C5 | C19H26O2 | 286.41 | 4.68 | 2 | 2 | 1 | 29.46 | 0 | 0 | |
| C7 | C21H30O2 | 314.46 | 5.33 | 4 | 2 | 1 | 29.46 | 1 | 0 | |
| C11 | C22H30O4 | 358.47 | 4.94 | 5 | 4 | 2 | 66.76 | 0 | 0 | |
| E10 | C22H3O7 | 379.26 | 0.78 | 5 | 7 | 3 | 80.14 | 0 | 0 | |
| E18 | C17H2NO2 | 252.20 | 2.40 | 4 | 2 | 1 | 30.49 | 0 | 0 | |
| E19 | C18H2NO3 | 280.21 | 2.30 | 5 | 3 | 1 | 39.72 | 0 | 0 | |
| E51 | C27H2N2O5 | 434.32 | 1.53 | 6 | 6 | 2 | 82.53 | 0 | 0 | |
| E57 | C19ClNO2 | 309.66 | 3.42 | 5 | 3 | 0 | 31.35 | 0 | 0 | |
| E58 | C27HO5 | 405.29 | 3.77 | 11 | 5 | 1 | 57.15 | 0 | 1 | |
| E59 | C27HClN2O4 | 452.76 | 2.83 | 9 | 5 | 1 | 59.00 | 0 | 0 | |
| M110 | C21H9O11 | 437.2 | -1.27 | 5 | 11 | 6 | 164.75 | 1 | 1 | |
| M17 | C28H8O15 | 584.35 | -1.98 | 7 | 15 | 8 | 234.29 | 3 | 1 | |
| M293 | C29H8O17 | 628.36 | -1.86 | 11 | 17 | 8 | 125.81 | 3 | 2 | |
| M319 | C27H10O16 | 590.36 | -2.60 | 7 | 16 | 10 | 269.43 | 3 | 1 | |
| M322 | C27H9O15 | 573.35 | -2.32 | 7 | 15 | 9 | 249.20 | 3 | 1 | |
| M292 | C26H8O14 | 544.33 | -1.75 | 7 | 14 | 8 | 111.05 | 3 | 1 | |
| M324 | C36H30O16 | 718.61 | 2.23 | 14 | 16 | 9 | 278.04 | 3 | 2 | |
| M378 | C36H9O16 | 697.45 | 1.16 | 14 | 16 | 9 | 278.04 | 3 | 2 | |
| M269 | C29H6O9 | 498.35 | -0.22 | 4 | 9 | 6 | 164.75 | 0 | 1 | |
| M285 | C27H42O7 | 478.62 | 1.76 | 2 | 7 | 5 | 127.59 | 0 | 0 | |
| M16 | C27H8O14 | 556.34 | -1.97 | 6 | 14 | 8 | 225.06 | 3 | 1 | |
| M320 | C21H7O11 | 435.27 | -0.72 | 4 | 11 | 7 | 190.28 | 2 | 1 | |
| M321 | C21H7O12 | 451.27 | -0.85 | 4 | 7 | 12 | 207.35 | 2 | 1 | |
| M286 | C16H5O9 | 341.21 | 1.07 | 3 | 9 | 5 | 145.91 | 0 | 1 | |
| M274 | C24H32O6 | 416.51 | 1.93 | 1 | 6 | 3 | 104.06 | 0 | 0 | |
| M109 | C25H24O12 | 516.45 | 0.80 | 9 | 12 | 7 | 211.28 | 3 | 1 | |
| M111 | C25H24O12 | 516.45 | 0.63 | 9 | 12 | 7 | 211.28 | 3 | 1 | |
| M270 | C29H4O8 | 480.34 | 0.40 | 4 | 8 | 4 | 141.36 | 0 | 1 | |
| M267 | C27H7O8 | 459.34 | -0.56 | 5 | 8 | 7 | 158.68 | 1 | 1 | |
| M268 | C29H5O8 | 481.35 | 0.31 | 4 | 8 | 5 | 144.52 | 0 | 1 | |
| M295 | C25H9O15 | 549.33 | -2.19 | 2 | 15 | 9 | 249.20 | 3 | 1 | |
| M148 | C25H32O13 | 540.51 | 0.13 | 11 | 13 | 6 | 201.67 | 3 | 2 | |
| M22 | C15H10O6 | 286.24 | 1.73 | 1 | 6 | 4 | 111.13 | 0 | 0 | |
| M276 | C15H6O8 | 314.20 | 0.41 | 1 | 8 | 6 | 151.59 | 1 | 1 | |
| M329 | C17H14O7 | 330.29 | 2.11 | 3 | 7 | 3 | 109.36 | 0 | 0 | |
| M294 | C15H10O5 | 270.24 | 2.11 | 1 | 5 | 3 | 90.90 | 0 | 0 | |
| M81 | C28H38O19 | 678.59 | 0.12 | 21 | 19 | 0 | 238.09 | 2 | 2 | |
| Ref.^1^ | C16H28N2O4 | 312.40 | 1.38 | 9 | 5 | 2 | 90.65 | 0 | 0 | |
| Ref.^2^ | C8H12N4O5 | 244.20 | -2.14 | 3 | 7 | 4 | 143.72 | 0 | 1 | |
|  |  |  | ≤ 5 | ≤10 | ≤ 10 | ≤ 5 | ≤ 140 | ≤2 | ≤1 | |
