# Supplementary material for: Repositioning antiviral phytoconstituents as broad-spectrum inhibitors of influenza A neuraminidase and human metapneumovirus fusion protein
Source: PLoS One. 2026 Sep 2;21(9):e0348517. doi: 10.1371/journal.pone.0348517 (PMC13537546; doi:10.1371/journal.pone.0348517)
Supplement: Supplementary file 7 [file pone.0348517.s004.docx]

**Comments**

Introduction

1. For the paragraph starting with “Viral respiratory infections remain a global public health problem. Influenza A virus and human metapneumovirus (HMPV) are …antiviral therapeutic strategies”. This section appears wordy with repetition (e.g., "high mortality and morbidity"; "epidemiological impact"). Sentences such as "...influenza A, in particular, has been known..." appear fragmented, with comma splices. Another sentence, "...leading to high mortality... particularly among vulnerable individuals such as children and the elderly..." appears awkwardly phrased. The overall section may require rewording for improved readability and originality.

2. For “Neuraminidase (NA) is an enzyme and a well-known viral target, having been the subject of

extensive research and development of treatments for influenza A, such as oseltamivir and

zanamivir. “The original sentence appears long, dense, and wordy. Phrases like "well-known viral target" are common. Rephrasing the sentence by replacing "well-known" with "key" would add to originality.

Material and methods

1. Provide a clear flow of how the phytochemicals were screened. Kindly provide a list of databases used for shortlisting and screening the phytochemicals. Also mention the total number of phytochemicals/phytoconstituents that were used for the purpose of screening.

2. Since Neuraminidase and fusion proteins are common targets, provide a clear rationale or criteria for selecting them over other target receptors.

3. Kindly note the nomenclature style for PDB entries. Although all capitals are not mandatory, uppercase is the standard convention.

4. Both structures of neuraminidase and fusion protein have a resolution of more than 2.5 Angstroms. Please check for structures with better resolution.

5. Were both the proteins subject to energy minimization before docking? If so, then kindly mention that in the methods

6. Kindly check for spacing. In section 2.2.2. “Autodocktools “

7. For Figure 2 B, it would be recommended to modify the image of the protein to highlight the binding residues better. Also mention (a) and (b) in the figure.

8. In section 2.5, please check the position of the charges. They need to be superscripted. Kindly check the entire manuscript for the same and maintain consistency. “Na+ and Cl− ions “

Results

1. Figures of the protein-ligand docked complexes need to be shown. And the molecular docking results need to be discussed with more clarity.

2. The RMSD graphs appear to have been made using Microsoft Excel. The graphs need to be more refined and clearer. Kindly use Discovery Studio for better high-resolution and sharper images for the graphs.

**Overall comment:**

The manuscript reflects average work using phytochemicals to target already well-established and known receptor targets like Neuraminidase. A clear rationale is required to explain why already known targets were considered and no novel targets were identified and considered. This raises concerns regarding the novelty of the submitted work. Moreover, the data and results are not well-structured.

The Authors need to revisit their work and provide comprehensive details with clarity regarding the choice of the receptors and ligands.

Overall, the manuscript appears incomplete and lacks novelty and may need to be thoroughly revised before submitting.
