# Supplementary material for: Repositioning antiviral phytoconstituents as broad-spectrum inhibitors of influenza A neuraminidase and human metapneumovirus fusion protein
Source: PLoS One. 2026 Sep 2;21(9):e0348517. doi: 10.1371/journal.pone.0348517 (PMC13537546; doi:10.1371/journal.pone.0348517)
Supplement: Supplementary file 8 [file pone.0348517.s008.docx]

Comments and suggestions to the author

1. The abstract can be refined further.
2. In the introduction, sentences like "Phytoconstituents are an in... these phytoconstituents" can be refined
3. In section 2.1.2, “F currently lacks approved therapeutics…” needs to be revised. Although the second fusion protein has been identified as F, starting a sentence directly with F is not correct. Kindly consider changing to “furthermore, protein F…” or “Moreover, protein F…” for better readability.
4. In section 2.2.1, kindly check the format and font style of “10⁻⁴ units”. It appears inconsistent.
5. In section 2.2.1, kindly maintain a consistent language style of either American English or British English as instructed by the journal guidelines. Also, perform a spell check.

“… geometry optimization and energy minimisation were performed in Avocadro using”

6. In section 2.2.2, kindly change …” deduced using Discovery Studio 2021” to “…deduced using Discovery Studio, 2021”
