# Supplementary material for: Repositioning antiviral phytoconstituents as broad-spectrum inhibitors of influenza A neuraminidase and human metapneumovirus fusion protein
Source: PLoS One. 2026 Sep 2;21(9):e0348517. doi: 10.1371/journal.pone.0348517 (PMC13537546; doi:10.1371/journal.pone.0348517)
Supplement: Supplementary file 9 [file pone.0348517.s009.docx]

**General response:**

We sincerely thank the reviewer for their constructive comments and positive assessment of our manuscript. We have addressed each point in detail, clarifying methodological aspects, improving figures and tables, and ensuring consistency throughout the manuscript. All modifications made in response to the reviewer’s comments have been highlighted in yellow in the revised manuscript. Specific responses to each comment are provided below.

**Reviewer 1:**

**1. Graphical abstract**

The graphical abstract is clear but would benefit from additional quantitative detail. I recommend that the authors indicate the number of compounds at key stages of the workflow (initial library, post-docking/ADMET filtering, and final hits) directly in the figure. Adding these counts will improve transparency of the screening process and help readers quickly understand the scale and progression of the study from raw phytochemicals to proposed lead candidates.

**1. Author response:** We have updated the graphical abstract to indicate the number of compounds at each stage: initial library (121 compounds), post-docking filtering (37 compounds), post-ADMET filtering (6 compounds), and final hits (2 compounds). This provides a clear overview of the screening process.

# 2. Rationale for target selection (NA and F)

In the Introduction, the rationale for selecting neuraminidase (NA) and HMPV fusion protein (F) as targets—particularly in the context of the authors’ prior work on hemagglutinin (HA) and SARS-CoV-2 Mpro—is not fully articulated. The authors should expand the relevant paragraph to: (i) explain how targeting NA and F complements or extends the previous HA/Mpro findings, (ii) justify these proteins as logical choices for a broad-spectrum antiviral strategy, and (iii) clarify how they address distinct, non-redundant steps in the viral life cycle (entry/fusion vs. release). This will strengthen the conceptual framework of the study.

**2. Author response: We have expanded the Introduction to justify our target selection. Specifically, we explain that NA and F target distinct steps (viral release and entry/fusion, respectively) and complement our previous work on HA and Mpro. This strengthens the rationale for pursuing a broad-spectrum antiviral strategy.**

# 3. Ligand preparation: optimization details

The Methods state that “for the ligands, the MMFF94 force field and the gradient descent algorithm implemented in Avogadro were applied.” To enhance reproducibility, the authors should specify the convergence criteria used for ligand geometry optimization (e.g., energy or gradient threshold, maximum number of steps, and any stopping conditions). These details are important for others wishing to replicate the ligand preparation workflow.

**3. Author response:** We thank the reviewer for this valuable comment. To improve reproducibility, the Methods section has been revised to include the detailed ligand geometry optimization parameters used during structure preparation. Specifically, ligand geometry optimization and energy minimization were performed in Avogadro using the MMFF94 force field and the conjugate gradient algorithm with a simple line-search method. The convergence criterion was set to an energy threshold of 10⁻⁴ with a maximum of 2500 optimization steps. Following optimization, ligands were prepared in AutoDockTools by assigning Gasteiger charges and adding polar hydrogen atoms. These details have now been incorporated into the revised manuscript to ensure better transparency and reproducibility of the ligand preparation workflow.

# 4. ADMET: definition of “optimal water solubility”

In the ADMET section, the phrase “all the compounds studied… have optimal water solubility” is vague. The term “optimal” should be replaced or explicitly mapped to the solubility categories used in Table 4 (e.g., soluble, moderately soluble, partially soluble). I suggest that the authors (i) state how many compounds fall into each category and (ii) clearly describe the exceptions (C11, E59, M22) using the same terminology as in Table 4, to ensure consistency between text and table.

**4. Author response:** We thank the reviewer for this valuable comment. The text now defines solubility categories (soluble, moderately soluble, poorly soluble) as in Table 4. Exceptions (C11, E59, M22) are explicitly described using this terminology.

# 5. Justification for retaining C11, C5, and M274

The conclusion that “compounds C11, C5, and M274 are generally considered acceptable… justifying their inclusion in the list of candidates for further exploration” is reasonable but should be made more explicit. I recommend that the authors:

- Restate the specific toxicity issues for each compound (C11 and C5: only LD50 < 1000 mg/kg; M274: only a carcinogenicity flag, with other endpoints inactive).
- Clearly indicate that these are **in silico** leads for further investigation, not ready drug candidates, given the presence of these safety flags.
- Optionally note that their overall ADME/T profiles are more favorable than some discarded molecules and at least one reference drug, which supports their retention as exploratory leads.

These clarifications will make the selection criteria more transparent.

**5. Author response:** We thank the reviewer for this valuable comment. The revised manuscript now provides a clearer justification for retaining compounds C11, C5, and M274 as exploratory in silico leads for further investigation, based on their overall favorable ADME/T profiles relative to several discarded compounds and reference drugs.

# 6. ProTox-II outputs: “active/inactive” and probability

In Table 5, ProTox-II results are reported only as “active” or “inactive” for each toxicity endpoint (neurotoxicity, cytotoxicity, carcinogenicity, mutagenicity, hepatotoxicity), without explanation. The authors should:

- Clarify in the Methods or a table footnote that “active” denotes a positive toxicity prediction for that endpoint and “inactive” denotes a negative prediction.
- Include the ProTox-II prediction probabilities (or accuracy/confidence scores) for these calls, at least for key candidate compounds, and specify the probability threshold considered meaningful (e.g., >0.70).

Reporting and interpreting these probabilities will help readers assess the robustness of the toxicity predictions.

**6. Author response:** We now clarify that “active” denotes a positive toxicity prediction, “inactive” a negative prediction. For key candidate compounds, ProTox-II probabilities are included, with a threshold of >0.70 considered meaningful.

# 7. Figure 3a: missing legends

In Figure 3a (RMSD of 7sej complexes), some curves appear to lack corresponding entries in the legend, which makes it difficult to identify each protein–ligand trajectory. The authors should ensure that every RMSD curve is clearly assigned to a ligand in the legend (or directly annotated) and that the legend matches the ligands discussed in the Results.

**7. Author response:** All RMSD curves are now clearly labeled in the legend and annotated in the figure to match the ligands discussed in Results.

**Reviewer 2:**

**Introduction**

**1. For the paragraph starting with “Viral respiratory infections remain a global public health problem. Influenza A virus and human metapneumovirus (HMPV) are …antiviral therapeutic strategies”. This section appears wordy with repetition (e.g., "high mortality and morbidity"; "epidemiological impact"). Sentences such as "...influenza A, in particular, has been known..." appear fragmented, with comma splices. Another sentence, "...leading to high mortality... particularly among vulnerable individuals such as children and the elderly..." appears awkwardly phrased. The overall section may require rewording for improved readability and originality.**

**1. Author response:** We thank the reviewer for this insightful comment. The paragraph has been revised to improve clarity, readability, and overall flow by removing redundancies, correcting sentence structure issues, and enhancing the academic writing style.

**2. For “Neuraminidase (NA) is an enzyme and a well-known viral target, having been the subject of extensive research and development of treatments for influenza A, such as oseltamivir and zanamivir. “The original sentence appears long, dense, and wordy. Phrases like "well-known viral target" are common. Rephrasing the sentence by replacing "well-known" with "key" would add to originality.**

**2. Author response:** We thank the reviewer for this helpful suggestion. The sentence has been revised to improve clarity and conciseness, and the phrase “well-known viral target” has been replaced with “key viral target” to enhance originality and readability.

**Material and methods**

**1. Provide a clear flow of how the phytochemicals were screened. Kindly provide a list of databases used for shortlisting and screening the phytochemicals. Also mention the total number of phytochemicals/phytoconstituents that were used for the purpose of screening.**

**1. Author response:** We thank the reviewer for this valuable comment. The manuscript has been revised to provide a clearer description of the phytochemical screening workflow. Specifically, we have clarified the flow of compound selection and included the databases used for phytochemical retrieval and shortlisting. In addition, the total number of phytochemicals used for virtual screening has now been explicitly stated in the revised manuscript.

**2. Since Neuraminidase and fusion proteins are common targets, provide a clear rationale or criteria for selecting them over other target receptors.**

**2. Author response:** We thank the reviewer for this valuable comment. The manuscript has been revised to include a clearer rationale for selecting neuraminidase and the HMPV fusion protein as target receptors. These targets were chosen due to their essential roles in distinct stages of the viral life cycle (viral entry/fusion and viral release), their established relevance as antiviral drug targets (in the case of neuraminidase), and the structural and functional importance of the HMPV fusion protein, which currently lacks approved therapeutics. The revised section also clarifies the multi-target strategy adopted in this study and its relevance for evaluating potential broad-spectrum antiviral phytoconstituents.

**3. Kindly note the nomenclature style for PDB entries. Although all capitals are not mandatory, uppercase is the standard convention.**

**3. Author response:** We thank the reviewer for this helpful comment. The manuscript has been revised to ensure consistency in the nomenclature of Protein Data Bank (PDB) entries, and all PDB identifiers have been formatted according to the standard convention in uppercase throughout the manuscript.

**4. Both structures of neuraminidase and fusion protein have a resolution of more than 2.5 Angstroms. Please check for structures with better resolution.**

**4. Author response:** We acknowledge the reviewer’s comment regarding the resolution of the PDB structures. Although the structures used—2HT7 for influenza A neuraminidase (2.60 Å) and 7SEJ for HMPV fusion protein (2.51 Å)—have resolutions slightly above 2.5 Å, they represent the **highest-resolution experimentally determined structures currently available** for these specific targets. Both structures have been widely employed in published studies for **molecular docking and molecular dynamics simulations**, demonstrating their reliability for computational analyses. Given these factors, these structures were deemed **appropriate and scientifically valid** for the present study.

**5. Were both the proteins subject to energy minimization before docking? If so, then kindly mention that in the methods**

**5. Author response:** We thank the reviewer for this comment. Ligand structures were geometry-optimized and energy-minimized prior to docking using the MMFF94 force field implemented in Avogadro. In contrast, protein structures were not subjected to energy minimization before docking. Only standard protein preparation steps were performed using AutoDockTools, including removal of water molecules and co-crystallized ligands, addition of hydrogen atoms, and assignment of Kollman charges. The Materials and Methods section, specifically subsection 2.2 (Dataset Preparation), including 2.2.1 (Ligands and Protein Preparation), has been revised accordingly to clarify the ligand preparation procedure and to explicitly state that no energy minimization was performed on the protein structures prior to docking.

**6. Kindly check for spacing. In section 2.2.2. “Autodocktools “**

**6. Author response:** We thank the reviewer for this helpful comment. The manuscript has been carefully proofread and corrected to fix spacing and formatting issues throughout the text, including the term “AutoDockTools” in section 2.2.2, to ensure consistency and adherence to proper nomenclature.

**7. For Figure 2 B, it would be recommended to modify the image of the protein to highlight the binding residues better. Also mention (a) and (b) in the figure.**

**7. Author response:** We thank the reviewer for this helpful suggestion. Figure 2 has been revised to improve the visualization of the binding sites, with better highlighting of the interacting residues to enhance clarity. In addition, the figure has been updated to include panel labels (a) and (b) to better distinguish the two protein structures.

**8. In section 2.5, please check the position of the charges. They need to be superscripted. Kindly check the entire manuscript for the same and maintain consistency. “Na+ and Cl− ions “**

**8. Author response:** We thank the reviewer for this helpful comment. The manuscript has been carefully revised to correct the formatting of ionic charges, and all charges (e.g., Na⁺ and Cl⁻) have been superscripted consistently throughout the text.

**Results**

**1. Figures of the protein-ligand docked complexes need to be shown. And the molecular docking results need to be discussed with more clarity.**

**1. Author response:** We thank the reviewer for this valuable suggestion. In response, representative 2D interaction diagrams of the best multitarget docked complexes, along with the reference compounds, have been added to the revised manuscript (Figure 3). These figures illustrate the binding modes and key interactions established within the active sites of both NA and HMPV-F proteins. In addition, the molecular docking section has been revised to provide a clearer discussion of the docking results and ligand–protein interactions. The updated discussion now highlights the main interaction types involved in complex stabilization, including hydrogen bonding, hydrophobic contacts, π-interactions, and electrostatic interactions, as well as the key amino acid residues participating in ligand binding. Furthermore, the superior binding affinities of compounds such as E51 and M110 relative to the reference drugs are now discussed in greater detail to better support their potential multitarget antiviral activity.

**2. The RMSD graphs appear to have been made using Microsoft Excel. The graphs need to be more refined and clearer. Kindly use Discovery Studio for better high-resolution and sharper images for the graphs.**

**2. Author response:** We thank the reviewer for this helpful suggestion. The RMSD plots have been revised to improve clarity and overall visual presentation, including refined formatting and enhanced readability. The underlying RMSD data remain unchanged.
