# Supplementary material for: Repositioning antiviral phytoconstituents as broad-spectrum inhibitors of influenza A neuraminidase and human metapneumovirus fusion protein
Source: PLoS One. 2026 Sep 2;21(9):e0348517. doi: 10.1371/journal.pone.0348517 (PMC13537546; doi:10.1371/journal.pone.0348517)
Supplement: Supplementary file 11 [file pone.0348517.s011.docx]

**Response to the Reviewers**

We sincerely thank the Editor and the Reviewers for their careful evaluation of our manuscript and for their valuable comments and constructive suggestions. We greatly appreciate the time and effort devoted to reviewing our work. Their insightful recommendations have helped us improve the quality, clarity, and scientific rigor of the manuscript. All comments have been carefully addressed, and the manuscript has been revised accordingly. The modifications introduced in the revised version are **highlighted in yellow** to facilitate their identification. A detailed, point-by-point response to each comment is provided below.

Comments and suggestions to the author

1. The abstract can be refined further.

**Response:** Thank you for your comment. The abstract has been revised to improve its clarity, conciseness, and overall readability while preserving the scientific content.

2. In the introduction, sentences like "Phytoconstituents are an in... these phytoconstituents" can be refined

**Response:** Thank you for your suggestion. The indicated sentences have been revised to improve clarity, readability, and overall language flow in the Introduction.

3. In section 2.1.2, “F currently lacks approved therapeutics…” needs to be revised. Although the second fusion protein has been identified as F, starting a sentence directly with F is not correct. Kindly consider changing to “furthermore, protein F…” or “Moreover, protein F…” for better readability.

**Response:** Thank you for your suggestion. The sentence has been revised by replacing “F” with “Moreover, protein F…” to improve readability and sentence structure.

4. In section 2.2.1, kindly check the format and font style of “10⁻⁴ units”. It appears inconsistent.

**Response:** Thank you for pointing this out. The formatting and font style of “10⁻⁴ units” have been corrected to ensure consistency throughout the manuscript.

5. In section 2.2.1, kindly maintain a consistent language style of either American English or British English as instructed by the journal guidelines. Also, perform a spell check.

“… geometry optimization and energy minimisation were performed in Avocadro using”

**Response:** Thank you for your comment. The manuscript has been thoroughly proofread, and the language has been revised to ensure consistent American English usage throughout, in accordance with the journal guidelines. Spelling and terminology have also been corrected where necessary.

6. In section 2.2.2, kindly change …” deduced using Discovery Studio 2021” to “…deduced using Discovery Studio, 2021

**Response:** Thank you for your suggestion. The sentence has been revised to read “deduced using Discovery Studio, 2021” as recommended.

**Reviewer #3: Major Comments:**
**1**. The novelty of the study is not clearly established. The work primarily involves selecting a subset of phytoconstituents from an existing database followed by molecular docking, which does not sufficiently demonstrate innovation. Experimental validation through in vitro antiviral assays is necessary to support the proposed therapeutic potential of the identified compounds.

**Response:** We sincerely thank the reviewer for this constructive comment. We agree that the novelty of the study should be stated more explicitly. Accordingly, the **Introduction** and **Section 2.1.2 (Biological Target)** were revised to better emphasize the innovative aspects of our study, and the **Conclusion** was updated to further clarify the originality of the proposed computational strategy and the scope of the findings (highlighted in yellow in the revised manuscript).

The novelty of this study does not reside in the use of previously reported phytoconstituents alone, but rather in the integrated computational strategy applied to identify broad-spectrum antiviral candidates. Specifically, our work simultaneously investigates two distinct respiratory viruses, influenza A virus and human metapneumovirus (HMPV), by targeting two key viral proteins involved in different stages of the viral life cycle, namely neuraminidase (NA) and the fusion protein (F). To the best of our knowledge, computational studies combining these two unrelated viral targets within a single screening workflow remain limited.

In addition, our study extends beyond conventional molecular docking by integrating successive filtering through drug-likeness assessment, comprehensive ADMET and toxicity prediction, followed by molecular dynamics simulations and MM-PBSA binding free-energy calculations to evaluate the stability of the selected protein–ligand complexes. This sequential workflow enabled the rational prioritization of the most promising candidates rather than relying solely on docking scores.

Regarding the request for experimental validation, we fully agree that in vitro antiviral assays are essential to confirm the computational predictions. However, the present work was designed as an in silico screening study aimed at identifying and prioritizing promising lead compounds for future experimental investigations. We have therefore revised the **Conclusion** to emphasize that the proposed compounds should be considered computational lead candidates requiring subsequent experimental validation, particularly through **in vitro antiviral assays**, followed by **in vivo** investigations, before any therapeutic conclusions can be drawn.

**2**. The claim that several phytochemicals outperform oseltamivir and ribavirin is based primarily on docking scores. Since docking energy alone does not reliably predict biological potency, additional binding free-energy calculations or experimental validation are needed.

**Response:** We sincerely thank the reviewer for this valuable comment. We agree that molecular docking scores alone are not sufficient to predict biological potency and should not be interpreted as evidence of superior antiviral activity. Accordingly, we have revised the manuscript to avoid overinterpretation of the docking results. Throughout the manuscript (Abstract, Results, and Conclusion), statements suggesting that the selected phytoconstituents "outperformed" the reference drugs have been replaced with more appropriate wording indicating more favorable predicted binding energies.

In response to the reviewer's recommendation, we performed MM-PBSA binding free-energy calculations as an additional validation step. The corresponding methodology and results have been incorporated into the revised manuscript (**Sections 2.5 and 3.3.4, Table 7**). The MM-PBSA analysis provided an independent energetic assessment of the selected protein–ligand complexes and further supported the molecular docking and molecular dynamics findings, reinforcing the prioritization of M294 and C11 as the most promising broad-spectrum antiviral lead compounds.

Furthermore, we clarified that the prioritization of the selected compounds was not based solely on docking scores but on an integrated computational workflow combining molecular docking, drug-likeness evaluation, ADMET and toxicity profiling, molecular dynamics simulations, and MM-PBSA binding free-energy calculations to improve the reliability of candidate selection.

Regarding the reviewer's recommendation for experimental validation, we fully agree that in vitro and in vivo studies are essential to confirm the computational predictions. However, the present study was designed as an in silico screening study for lead identification. Therefore, we have revised the Conclusion to explicitly state that the identified compounds should be considered computational lead candidates whose predicted antiviral potential requires experimental validation, particularly through in vitro antiviral assays followed by in vivo investigations.

**3**. The rationale for selecting the final lead compounds (M294 and C11) should be presented more systematically by integrating docking, ADMET, toxicity, and molecular dynamics results into a clear ranking or scoring framework.

**Response:** We sincerely thank the reviewer for this valuable suggestion. We agree that the rationale for selecting the final lead compounds should be presented more systematically. Accordingly, we have revised the manuscript to clarify the sequential prioritization strategy adopted throughout the study.

Specifically, we have added a summary table entitled **"Sequential prioritization framework for the selection of the final broad-spectrum antiviral lead compounds (Table 8.** )**"**, which integrates the successive selection criteria, including molecular docking, drug-likeness evaluation, ADMET and toxicity profiling, molecular dynamics analyses and MM-PBSA binding free-energy calculations. This framework clearly illustrates how the initial library of phytoconstituents was progressively narrowed down to the final lead compounds.

In addition, the end of the Molecular Dynamics section has been revised to explicitly explain that M294 and C11 were prioritized because they consistently fulfilled all selection criteria by combining favorable multi-target docking performance, acceptable predicted pharmacokinetic and toxicological profiles, and the most stable interactions with both viral targets during the molecular dynamics simulations.

**4**. The molecular dynamics analysis mainly reports RMSD, RMSF, and interaction profiles, but lacks complementary analyses such as MM-PBSA/MM-GBSA binding free-energy calculations.

**Response:** Thank you for this valuable suggestion. In response to the reviewer's comment, we performed MM-PBSA binding free-energy calculations as a complementary analysis to the molecular dynamics simulations. The MM-PBSA results have been incorporated into the revised manuscript and provide a quantitative estimation of the binding free energies of the selected protein–ligand complexes. These calculations further support the stability and binding affinity observed from the RMSD, RMSF, and protein–ligand interaction analyses, reinforcing the prioritization of M294 and C11 as the most promising broad-spectrum antiviral candidates. The corresponding methodology, results, and discussion have been added to the revised manuscript (**Section 3.3.4 , Table 7**).

**5**. The manuscript does not adequately discuss the limitations of using predicted ADMET and toxicity data, which should be interpreted cautiously.

**Response:** We sincerely thank the reviewer for this valuable comment. We agree that computational ADMET and toxicity predictions should be interpreted with appropriate caution, as they are based on predictive models and cannot fully reflect the complexity of in vivo pharmacokinetic and toxicological behavior.

Accordingly, we have revised **Section 3.2.2 (Evaluation of pharmacokinetics and toxicity properties)** to explicitly acknowledge the limitations of these computational predictions. The revised manuscript has been updated to clarify that the predicted ADMET and toxicity profiles provide preliminary estimates that are valuable for early-stage compound prioritization but cannot replace experimental pharmacokinetic and toxicological evaluations. We also emphasize that the selected compounds should be considered exploratory in silico lead candidates requiring further experimental validation before any definitive conclusions regarding their safety or therapeutic potential can be drawn.

**6**. Although two viral proteins were investigated, the manuscript provides limited discussion on the conservation of the selected binding sites across different influenza A and HMPV strains.

**Response:** We sincerely thank the reviewer for this valuable comment. We agree that the conservation of the selected binding sites across different influenza A and HMPV strains deserved further clarification. Accordingly, we have revised **Section 2.1.2 (Biological Target)** to better justify the selection of the two crystallographic structures used in this study. Specifically, we added a discussion highlighting that the catalytic site of influenza A neuraminidase is highly conserved among the major influenza A subtypes, whereas the HMPV fusion (F) protein contains conserved functional regions across the principal HMPV genotypes. These characteristics support the use of PDB structures **2HT7** and **7SEJ** as biologically relevant models for the computational identification of potential broad-spectrum antiviral candidates. We believe that this addition clarifies the rationale for target selection and strengthens the biological relevance of our computational approach.

**Minor Comments:**
**1**. The manuscript contains several grammatical errors and awkward sentence constructions that require careful English language editing to improve readability.

**Response:** We sincerely thank the reviewer for this valuable comment. The entire manuscript has been carefully revised to improve the quality of the English language, sentence structure, and overall readability. Grammatical errors, awkward sentence constructions, and stylistic inconsistencies were corrected throughout the text. In addition, the manuscript was thoroughly edited to ensure consistent use of American English spelling and terminology, thereby improving its clarity and readability.

**2**. The Introduction is longer than necessary and includes repeated discussion of previous studies, which could be condensed to improve focus on the objectives of the current work.

**Response:** We sincerely thank the reviewer for this constructive suggestion. The Introduction has been revised to improve its conciseness and focus. Repetitive background information, particularly regarding the clinical presentation of respiratory viral infections and the discussion of our previous studies, has been condensed or removed. The revised Introduction now places greater emphasis on the scientific rationale, the novelty of the proposed multi-target computational strategy, and the objectives of the present study.

**3.** The docking interaction figures would benefit from clearer labeling of interacting residues and improved annotation of key hydrogen bonds, hydrophobic contacts, and electrostatic interactions.

**Response:** We sincerely thank the reviewer for this helpful suggestion. The molecular docking interaction figures have been revised to improve their clarity and readability. The labeling of interacting amino acid residues has been enhanced, and the key hydrogen bonds, hydrophobic interactions, and electrostatic interactions have been more clearly annotated. These improvements facilitate the interpretation of the protein–ligand binding modes and highlight the principal interactions responsible for ligand stabilization within the binding sites.
