# Supplementary material for: Repositioning antiviral phytoconstituents as broad-spectrum inhibitors of influenza A neuraminidase and human metapneumovirus fusion protein
Source: PLoS One. 2026 Sep 2;21(9):e0348517. doi: 10.1371/journal.pone.0348517 (PMC13537546; doi:10.1371/journal.pone.0348517)
Supplement: Supplementary file 12 [file pone.0348517.s012.docx]

**Response to the Reviewers**

Dear Editorial Office,

Thank you for your email and for reviewing our revised submission.

We would like to confirm that we have now updated the manuscript files by uploading the latest revised version as the main article file, ensuring that it does not contain any tracked changes or highlighting, as requested.

We would also like to clarify that, to the best of our understanding, we had already addressed all of the reviewer comments that were communicated to us during the second round of revision. Since no additional reviewer comments were provided in this latest request, we did not make any further modifications to the manuscript itself. We explicitly indicated this in both the **"Enter Comments"** section of the online submission system and in the **Response to Reviewers and Editor** document accompanying our resubmission.

We hope that the updated files now meet all the submission requirements. Please let us know if any further action is required on our part.

Thank you for your time and consideration.

Kind regards,

Prof. Samir Chtita

Comments and suggestions to the author

1. The abstract can be refined further.

**Response:** Thank you for your comment. The abstract has been revised to improve its clarity, conciseness, and overall readability while preserving the scientific content.

2. In the introduction, sentences like "Phytoconstituents are an in... these phytoconstituents" can be refined

**Response:** Thank you for your suggestion. The indicated sentences have been revised to improve clarity, readability, and overall language flow in the Introduction.

3. In section 2.1.2, “F currently lacks approved therapeutics…” needs to be revised. Although the second fusion protein has been identified as F, starting a sentence directly with F is not correct. Kindly consider changing to “furthermore, protein F…” or “Moreover, protein F…” for better readability.

**Response:** Thank you for your suggestion. The sentence has been revised by replacing “F” with “Moreover, protein F…” to improve readability and sentence structure.

4. In section 2.2.1, kindly check the format and font style of “10⁻⁴ units”. It appears inconsistent.

**Response:** Thank you for pointing this out. The formatting and font style of “10⁻⁴ units” have been corrected to ensure consistency throughout the manuscript.

5. In section 2.2.1, kindly maintain a consistent language style of either American English or British English as instructed by the journal guidelines. Also, perform a spell check.

“… geometry optimization and energy minimisation were performed in Avocadro using”

**Response:** Thank you for your comment. The manuscript has been thoroughly proofread, and the language has been revised to ensure consistent American English usage throughout, in accordance with the journal guidelines. Spelling and terminology have also been corrected where necessary.

6. In section 2.2.2, kindly change …” deduced using Discovery Studio 2021” to “…deduced using Discovery Studio, 2021

**Response:** Thank you for your suggestion. The sentence has been revised to read “deduced using Discovery Studio, 2021” as recommended.
