# Supplementary material for: Repositioning antiviral phytoconstituents as broad-spectrum inhibitors of influenza A neuraminidase and human metapneumovirus fusion protein
Source: PLoS One. 2026 Sep 2;21(9):e0348517. doi: 10.1371/journal.pone.0348517 (PMC13537546; doi:10.1371/journal.pone.0348517)
Supplement: Peer review history

# Peer review: Decision Letter 0

**DOI:** 10.1371/journal.pone.0348517.r001

**Authors:** Lalit Samant

12 May 2026

Dear Dr. Chtita,

Thank you for submitting your manuscript to PLOS ONE. After careful consideration, we feel that it has merit but does not fully meet PLOS ONE’s publication criteria as it currently stands. Therefore, we invite you to submit a revised version of the manuscript that addresses the points raised during the review process.

Please submit your revised manuscript by Jun 26 2026 11:59PM. If you will need more time than this to complete your revisions, please reply to this message or contact the journal office at plosone@plos.org. When you're ready to submit your revision, log on to https://www.editorialmanager.com/pone/ and select the 'Submissions Needing Revision' folder to locate your manuscript file.

- A letter that responds to each point raised by the academic editor and reviewer(s). You should upload this letter as a separate file labeled 'Response to Reviewers'.
- A marked-up copy of your manuscript that highlights changes made to the original version. You should upload this as a separate file labeled 'Revised Manuscript with Track Changes'.
- An unmarked version of your revised paper without tracked changes. You should upload this as a separate file labeled 'Manuscript'.

If you would like to make changes to your financial disclosure, please include your updated statement in your cover letter. Guidelines for resubmitting your figure files are available below the reviewer comments at the end of this letter.

If applicable, we recommend that you deposit your laboratory protocols in protocols.io to enhance the reproducibility of your results. Protocols.io assigns your protocol its own identifier (DOI) so that it can be cited independently in the future. For instructions see: https://journals.plos.org/plosone/s/submission-guidelines#loc-laboratory-protocols. Additionally, PLOS ONE offers an option for publishing peer-reviewed Lab Protocol articles, which describe protocols hosted on protocols.io. Read more information on sharing protocols at https://plos.org/protocols?utm_medium=editorial-email&utm_source=authorletters&utm_campaign=protocols.

As the corresponding author, your ORCID iD is verified in the submission system and will appear in the published article. PLOS supports the use of ORCID, and we encourage all coauthors to register for an ORCID iD and use it as well. Please encourage your coauthors to verify their ORCID iD within the submission system before final acceptance, as unverified ORCID iDs will not appear in the published article. *Only*  the individual author can complete the verification step; PLOS staff *cannot*  verify ORCID iDs on behalf of authors.

We look forward to receiving your revised manuscript.

Kind regards,

Lalit Samant

Academic Editor

PLOS One

Journal Requirements:

When submitting your revision, we need you to address these additional requirements.

1. Please ensure that your manuscript meets PLOS ONE's style requirements, including those for file naming. The PLOS ONE style templates can be found at https://journals.plos.org/plosone/s/file?id=wjVg/PLOSOne_formatting_sample_main_body.pdf and https://journals.plos.org/plosone/s/file?id=ba62/PLOSOne_formatting_sample_title_authors_affiliations.pdf

2. Please note that PLOS One has specific guidelines on code sharing for submissions in which author-generated code underpins the findings in the manuscript. In these cases, we expect all author-generated code to be made available without restrictions upon publication of the work. Please review our guidelines at https://journals.plos.org/plosone/s/materials-and-software-sharing#loc-sharing-code and ensure that your code is shared in a way that follows best practice and facilitates reproducibility and reuse.

3. Thank you for stating in your Funding Statement:

[This work was supported by the Zayed Bin Sultan Center for Health Sciences and UAEU-University of Sharjah joint Grant (#12R282).].

Please provide an amended statement that declares *all* the funding or sources of support (whether external or internal to your organization) received during this study, as detailed online in our guide for authors at http://journals.plos.org/plosone/s/submit-now.  Please also include the statement “There was no additional external funding received for this study.” in your updated Funding Statement.

Please include your amended Funding Statement within your cover letter. We will change the online submission form on your behalf.

4. We note that the Graphical Abstract and Figure 1 in your submission contain images which may be copyrighted. All PLOS content is published under the Creative Commons Attribution License (CC BY 4.0), which means that the manuscript, images, and Supporting Information files will be freely available online, and any third party is permitted to access, download, copy, distribute, and use these materials in any way, even commercially, with proper attribution. For more information, see our copyright guidelines: http://journals.plos.org/plosone/s/licenses-and-copyright.

We require you to either (1) present written permission from the copyright holder to publish these figures specifically under the CC BY 4.0 license, or (2) remove the figures from your submission:

1. You may seek permission from the original copyright holder of the images in Graphical Abstract and Figure 1 to publish the content specifically under the CC BY 4.0 license.

We recommend that you contact the original copyright holder with the Content Permission Form (http://journals.plos.org/plosone/s/file?id=7c09/content-permission-form.pdf) and the following text:

“I request permission for the open-access journal PLOS ONE to publish XXX under the Creative Commons Attribution License (CCAL) CC BY 4.0 (http://creativecommons.org/licenses/by/4.0/). Please be aware that this license allows unrestricted use and distribution, even commercially, by third parties. Please reply and provide explicit written permission to publish XXX under a CC BY license and complete the attached form.”

Please upload the completed Content Permission Form or other proof of granted permissions as an "Other" file with your submission.

In the figure caption of the copyrighted figure, please include the following text: “Reprinted from [ref] under a CC BY license, with permission from [name of publisher], original copyright [original copyright year].”

2. If you are unable to obtain permission from the original copyright holder to publish these figures under the CC BY 4.0 license or if the copyright holder’s requirements are incompatible with the CC BY 4.0 license, please either i) remove the figure or ii) supply a replacement figure that complies with the CC BY 4.0 license. Please check copyright information on all replacement figures and update the figure caption with source information. If applicable, please specify in the figure caption text when a figure is similar but not identical to the original image and is therefore for illustrative purposes only.

5. Please include captions for your Supporting Information files at the end of your manuscript, and update any in-text citations to match accordingly. Please see our Supporting Information guidelines for more information: http://journals.plos.org/plosone/s/supporting-information.

If the reviewer comments include a recommendation to cite specific previously published works, please review and evaluate these publications to determine whether they are relevant and should be cited. There is no requirement to cite these works unless the editor has indicated otherwise.

Please review your reference list to ensure that it is complete and correct. If you have cited papers that have been retracted, please include the rationale for doing so in the manuscript text, or remove these references and replace them with relevant current references. Any changes to the reference list should be mentioned in the rebuttal letter that accompanies your revised manuscript. If you need to cite a retracted article, indicate the article’s retracted status in the References list and also include a citation and full reference for the retraction notice.

Additional Editor Comments:

Please go through each reviewer comments and address accordingly.

[Note: HTML markup is below. Please do not edit.]

Reviewers' comments:

Reviewer's Responses to Questions

**Comments to the Author**

1. Is the manuscript technically sound, and do the data support the conclusions?

Reviewer #1: Partly

Reviewer #2: Yes

**********

2. Has the statistical analysis been performed appropriately and rigorously?

Reviewer #1: N/A

Reviewer #2: Yes

**********

3. Have the authors made all data underlying the findings in their manuscript fully available?

The PLOS Data policy

Reviewer #1: Yes

Reviewer #2: Yes

**********

4. Is the manuscript presented in an intelligible fashion and written in standard English?

Reviewer #1: No

Reviewer #2: Yes

**********

Reviewer #1: The manuscript is fairly written with long sentences using common phrases. The writing lacks originality. Target receptors selected are common and standard proteins for influenza. No clear rationale or justification provided for selecting common receptors over novel ones. This would raise a question regarding the novelty of the work. Simply put, some phytohemicals are used to target already known and well established receptors. With respect to the methods, no clear selection criteria or approach has been mentioned for selecting the ligands. The energy minimization for the receptor proteins is not mentioned. Overall, the manuscript highlights average work and would require more robust and refined approach to highlight the novelty of the work. Also the language would need to be more refined with rewording and rephrasing, especially in the introduction

Reviewer #2: Recommendation: Accept with minor revisions

General assessment

This manuscript presents a well-executed in silico study repositioning antiviral phytoconstituents as potential broad spectrum inhibitors of influenza A neuraminidase (NA) and HMPV fusion protein (F). The work is clearly written, methodologically sound, and builds logically on the authors’ previous investigations of related phytochemical libraries. The docking, ADME/T profiling, and MD simulations are generally appropriate, and the conclusions are supported by the presented data. I believe the manuscript can be accepted after the authors address the minor issues listed below, which mainly concern clarity, completeness of methodological description, and alignment between text, tables, and figures.

1. Graphical abstract

The graphical abstract is clear but would benefit from additional quantitative detail. I recommend that the authors indicate the number of compounds at key stages of the workflow (initial library, post docking/ADMET filtering, and final hits) directly in the figure. Adding these counts will improve transparency of the screening process and help readers quickly understand the scale and progression of the study from raw phytochemicals to proposed lead candidates.

2. Rationale for target selection (NA and F)

In the Introduction, the rationale for selecting neuraminidase (NA) and HMPV fusion protein (F) as targets—particularly in the context of the authors’ prior work on hemagglutinin (HA) and SARS CoV 2 Mpro—is not fully articulated. The authors should expand the relevant paragraph to: (i) explain how targeting NA and F complements or extends the previous HA/Mpro findings, (ii) justify these proteins as logical choices for a broad spectrum antiviral strategy, and (iii) clarify how they address distinct, non redundant steps in the viral life cycle (entry/fusion vs. release). This will strengthen the conceptual framework of the study.

3. Ligand preparation: optimization details

The Methods state that “for the ligands, the MMFF94 force field and the gradient descent algorithm implemented in Avogadro were applied.” To enhance reproducibility, the authors should specify the convergence criteria used for ligand geometry optimization (e.g., energy or gradient threshold, maximum number of steps, and any stopping conditions). These details are important for others wishing to replicate the ligand preparation workflow.

4. ADMET: definition of “optimal water solubility”

In the ADMET section, the phrase “all the compounds studied… have optimal water solubility” is vague. The term “optimal” should be replaced or explicitly mapped to the solubility categories used in Table 4 (e.g., soluble, moderately soluble, poorly/partially soluble). I suggest that the authors (i) state how many compounds fall into each category and (ii) clearly describe the exceptions (C11, E59, M22) using the same terminology as in Table 4, to ensure consistency between text and table.

5. Justification for retaining C11, C5, and M274

The conclusion that “compounds C11, C5, and M274 are generally considered acceptable… justifying their inclusion in the list of candidates for further exploration” is reasonable but should be made more explicit. I recommend that the authors:

• Restate the specific toxicity issues for each compound (C11 and C5: only LD50 < 1000 mg/kg; M274: only a carcinogenicity flag, with other endpoints inactive).

• Clearly indicate that these are in silico leads for further investigation, not ready drug candidates, given the presence of these safety flags.

• Optionally note that their overall ADME/T profiles are more favorable than some discarded molecules and at least one reference drug, which supports their retention as exploratory leads.

These clarifications will make the selection criteria more transparent.

6. ProTox-II outputs: “active/inactive” and probability

In Table 5, ProTox-II results are reported only as “active” or “inactive” for each toxicity endpoint (neurotoxicity, cytotoxicity, carcinogenicity, mutagenicity, hepatotoxicity), without explanation. The authors should:

• Clarify in the Methods or a table footnote that “active” denotes a positive toxicity prediction for that endpoint and “inactive” denotes a negative prediction.

• Include the ProTox-II prediction probabilities (or accuracy/confidence scores) for these calls, at least for key candidate compounds, and specify the probability threshold considered meaningful (e.g., >0.70).

Reporting and interpreting these probabilities will help readers assess the robustness of the toxicity predictions.

7. Figure 3a: missing legends

In Figure 3a (RMSD of 7sej complexes), some curves appear to lack corresponding entries in the legend, which makes it difficult to identify each protein–ligand trajectory. The authors should ensure that every RMSD curve is clearly assigned to a ligand in the legend (or directly annotated) and that the legend matches the ligands discussed in the Results.

Overall recommendation:

The manuscript addresses an interesting and relevant topic, the data are solid, and the required changes are mainly clarifications and minor presentation improvements. Provided the authors satisfactorily address the above points, I recommend acceptance after minor revision.

**********

what does this mean?). If published, this will include your full peer review and any attached files.

If you choose “no”, your identity will remain anonymous but your review may still be made public.

**Do you want your identity to be public for this peer review?**  For information about this choice, including consent withdrawal, please see our Privacy Policy

Reviewer #1: No

Reviewer #2: No

**********

[NOTE: If reviewer comments were submitted as an attachment file, they will be attached to this email and accessible via the submission site. Please log into your account, locate the manuscript record, and check for the action link "View Attachments". If this link does not appear, there are no attachment files.]

To ensure your figures meet our technical requirements, please review our figure guidelines: https://journals.plos.org/plosone/s/figures

You may also use PLOS’s free figure tool, NAAS, to help you prepare publication quality figures: https://journals.plos.org/plosone/s/figures#loc-tools-for-figure-preparation.

NAAS will assess whether your figures meet our technical requirements by comparing each figure against our figure specifications.

Attachment Submitted filename: Comments.docx

Attachment Submitted filename: PONE-D-26-18338_reviewer.pdf

Attachment Submitted filename: Reviewer comments.pdf

# Author comment: Author response to Decision Letter 1

**DOI:** 10.1371/journal.pone.0348517.r002

1 Jun 2026

General response:

We sincerely thank the reviewer for their constructive comments and positive assessment of our manuscript. We have addressed each point in detail, clarifying methodological aspects, improving figures and tables, and ensuring consistency throughout the manuscript. All modifications made in response to the reviewer’s comments have been highlighted in yellow in the revised manuscript. Specific responses to each comment are provided below.

Reviewer 1:

1. Graphical abstract

The graphical abstract is clear but would benefit from additional quantitative detail. I recommend that the authors indicate the number of compounds at key stages of the workflow (initial library, post-docking/ADMET filtering, and final hits) directly in the figure. Adding these counts will improve transparency of the screening process and help readers quickly understand the scale and progression of the study from raw phytochemicals to proposed lead candidates.

1. Author response: We have updated the graphical abstract to indicate the number of compounds at each stage: initial library (121 compounds), post-docking filtering (37 compounds), post-ADMET filtering (6 compounds), and final hits (2 compounds). This provides a clear overview of the screening process.

2. Rationale for target selection (NA and F)

In the Introduction, the rationale for selecting neuraminidase (NA) and HMPV fusion protein (F) as targets—particularly in the context of the authors’ prior work on hemagglutinin (HA) and SARS-CoV-2 Mpro—is not fully articulated. The authors should expand the relevant paragraph to: (i) explain how targeting NA and F complements or extends the previous HA/Mpro findings, (ii) justify these proteins as logical choices for a broad-spectrum antiviral strategy, and (iii) clarify how they address distinct, non-redundant steps in the viral life cycle (entry/fusion vs. release). This will strengthen the conceptual framework of the study.

2. Author response: We have expanded the Introduction to justify our target selection. Specifically, we explain that NA and F target distinct steps (viral release and entry/fusion, respectively) and complement our previous work on HA and Mpro. This strengthens the rationale for pursuing a broad-spectrum antiviral strategy.

3. Ligand preparation: optimization details

The Methods state that “for the ligands, the MMFF94 force field and the gradient descent algorithm implemented in Avogadro were applied.” To enhance reproducibility, the authors should specify the convergence criteria used for ligand geometry optimization (e.g., energy or gradient threshold, maximum number of steps, and any stopping conditions). These details are important for others wishing to replicate the ligand preparation workflow.

3. Author response: We thank the reviewer for this valuable comment. To improve reproducibility, the Methods section has been revised to include the detailed ligand geometry optimization parameters used during structure preparation. Specifically, ligand geometry optimization and energy minimization were performed in Avogadro using the MMFF94 force field and the conjugate gradient algorithm with a simple line-search method. The convergence criterion was set to an energy threshold of 10_−⁴_ with a maximum of 2500 optimization steps. Following optimization, ligands were prepared in AutoDockTools by assigning Gasteiger charges and adding polar hydrogen atoms. These details have now been incorporated into the revised manuscript to ensure better transparency and reproducibility of the ligand preparation workflow.

4. ADMET: definition of “optimal water solubility”

In the ADMET section, the phrase “all the compounds studied… have optimal water solubility” is vague. The term “optimal” should be replaced or explicitly mapped to the solubility categories used in Table 4 (e.g., soluble, moderately soluble, partially soluble). I suggest that the authors (i) state how many compounds fall into each category and (ii) clearly describe the exceptions (C11, E59, M22) using the same terminology as in Table 4, to ensure consistency between text and table.

4. Author response: We thank the reviewer for this valuable comment. The text now defines solubility categories (soluble, moderately soluble, poorly soluble) as in Table 4. Exceptions (C11, E59, M22) are explicitly described using this terminology.

5. Justification for retaining C11, C5, and M274

The conclusion that “compounds C11, C5, and M274 are generally considered acceptable… justifying their inclusion in the list of candidates for further exploration” is reasonable but should be made more explicit. I recommend that the authors:

• Restate the specific toxicity issues for each compound (C11 and C5: only LD50 < 1000 mg/kg; M274: only a carcinogenicity flag, with other endpoints inactive).

• Clearly indicate that these are in silico leads for further investigation, not ready drug candidates, given the presence of these safety flags.

• Optionally note that their overall ADME/T profiles are more favorable than some discarded molecules and at least one reference drug, which supports their retention as exploratory leads.

These clarifications will make the selection criteria more transparent.

5. Author response: We thank the reviewer for this valuable comment. The revised manuscript now provides a clearer justification for retaining compounds C11, C5, and M274 as exploratory in silico leads for further investigation, based on their overall favorable ADME/T profiles relative to several discarded compounds and reference drugs.

6. ProTox-II outputs: “active/inactive” and probability

In Table 5, ProTox-II results are reported only as “active” or “inactive” for each toxicity endpoint (neurotoxicity, cytotoxicity, carcinogenicity, mutagenicity, hepatotoxicity), without explanation. The authors should:

• Clarify in the Methods or a table footnote that “active” denotes a positive toxicity prediction for that endpoint and “inactive” denotes a negative prediction.

• Include the ProTox-II prediction probabilities (or accuracy/confidence scores) for these calls, at least for key candidate compounds, and specify the probability threshold considered meaningful (e.g., >0.70).

Reporting and interpreting these probabilities will help readers assess the robustness of the toxicity predictions.

6. Author response: We now clarify that “active” denotes a positive toxicity prediction, “inactive” a negative prediction. For key candidate compounds, ProTox-II probabilities are included, with a threshold of >0.70 considered meaningful.

7. Figure 3a: missing legends

In Figure 3a (RMSD of 7sej complexes), some curves appear to lack corresponding entries in the legend, which makes it difficult to identify each protein–ligand trajectory. The authors should ensure that every RMSD curve is clearly assigned to a ligand in the legend (or directly annotated) and that the legend matches the ligands discussed in the Results.

7. Author response: All RMSD curves are now clearly labeled in the legend and annotated in the figure to match the ligands discussed in Results.

Reviewer 2:

Introduction

1. For the paragraph starting with “Viral respiratory infections remain a global public health problem. Influenza A virus and human metapneumovirus (HMPV) are …antiviral therapeutic strategies”. This section appears wordy with repetition (e.g., "high mortality and morbidity"; "epidemiological impact"). Sentences such as "...influenza A, in particular, has been known..." appear fragmented, with comma splices. Another sentence, "...leading to high mortality... particularly among vulnerable individuals such as children and the elderly..." appears awkwardly phrased. The overall section may require rewording for improved readability and originality.

1. Author response: We thank the reviewer for this insightful comment. The paragraph has been revised to improve clarity, readability, and overall flow by removing redundancies, correcting sentence structure issues, and enhancing the academic writing style.

2. For “Neuraminidase (NA) is an enzyme and a well-known viral target, having been the subject of extensive research and development of treatments for influenza A, such as oseltamivir and zanamivir. “The original sentence appears long, dense, and wordy. Phrases like "well-known viral target" are common. Rephrasing the sentence by replacing "well-known" with "key" would add to originality.

2. Author response: We thank the reviewer for this helpful suggestion. The sentence has been revised to improve clarity and conciseness, and the phrase “well-known viral target” has been replaced with “key viral target” to enhance originality and readability.

Material and methods

1. Provide a clear flow of how the phytochemicals were screened. Kindly provide a list of databases used for shortlisting and screening the phytochemicals. Also mention the total number of phytochemicals/phytoconstituents that were used for the purpose of screening.

1. Author response: We thank the reviewer for this valuable comment. The manuscript has been revised to provide a clearer description of the phytochemical screening workflow. Specifically, we have clarified the flow of compound selection and included the databases used for phytochemical retrieval and shortlisting. In addition, the total number of phytochemicals used for virtual screening has now been explicitly stated in the revised manuscript.

2. Since Neuraminidase and fusion proteins are common targets, provide a clear rationale or criteria for selecting them over other target receptors.

2. Author response: We thank the reviewer for this valuable comment. The manuscript has been revised to include a clearer rationale for selecting neuraminidase and the HMPV fusion protein as target receptors. These targets were chosen due to their essential roles in distinct stages of the viral life cycle (viral entry/fusion and viral release), their established relevance as antiviral drug targets (in the case of neuraminidase), and the structural and functional importance of the HMPV fusion protein, which currently lacks approved therapeutics. The revised section also clarifies the multi-target strategy adopted in this study and its relevance for evaluating potential broad-spectrum antiviral phytoconstituents.

3. Kindly note the nomenclature style for PDB entries. Although all capitals are not mandatory, uppercase is the standard convention.

3. Author response: We thank the reviewer for this helpful comment. The manuscript has been revised to ensure consistency in the nomenclature of Protein Data Bank (PDB) entries, and all PDB identifiers have been formatted according to the standard convention in uppercase throughout the manuscript.

4. Both structures of neuraminidase and fusion protein have a resolution of more than 2.5 Angstroms. Please check for structures with better resolution.

4. Author response: We acknowledge the reviewer’s comment regarding the resolution of the PDB structures. Although the structures used—2HT7 for influenza A neuraminidase (2.60 Å) and 7SEJ for HMPV fusion protein (2.51 Å)—have resolutions slightly above 2.5 Å, they represent the highest-resolution experimentally determined structures currently available for these specific targets. Both structures have been widely employed in published studies for molecular docking and molecular dynamics simulations, demonstrating their reliability for computational analyses. Given these factors, these structures were deemed appropriate and scientifically valid for the present study.

5. Were both the proteins subject to energy minimization before docking? If so, then kindly mention that in the methods

5. Author response: We thank the reviewer for this comment. Ligand structures were geometry-optimized and energy-minimized prior to docking using the MMFF94 force field implemented in Avogadro. In contrast, protein structures were not subjected to energy minimization before docking. Only standard protein preparation steps were performed using AutoDockTools, including removal of water molecules and co-crystallized ligands, addition of hydrogen atoms, and assignment of Kollman charges. The Materials and Methods section, specifically subsection 2.2 (Dataset Preparation), including 2.2.1 (Ligands and Protein Preparation), has been revised accordingly to clarify the ligand preparation procedure and to explicitly state that no energy minimization was performed on the protein structures prior to docking.

6. Kindly check for spacing. In section 2.2.2. “Autodocktools “

6. Author response: We thank the reviewer for this helpful comment. The manuscript has been carefully proofread and corrected to fix spacing and formatting issues throughout the text, including the term “AutoDockTools” in section 2.2.2, to ensure consistency and adherence to proper nomenclature.

7. For Figure 2 B, it would be recommended to modify the image of the protein to highlight the binding residues better. Also mention (a) and (b) in the figure.

7. Author response: We thank the reviewer for this helpful suggestion. Figure 2 has been revised to improve the visualization of the binding sites, with better highlighting of the interacting residues to enhance clarity. In addition, the figure has been updated to include panel labels (a) and (b) to better distinguish the two protein structures.

8. In section 2.5, please check the position of the charges. They need to be superscripted. Kindly check the entire manuscript for the same and maintain consistency. “Na+ and Cl− ions “

8. Author response: We thank the reviewer for this helpful comment. The manuscript has been carefully revised to correct the formatting of ionic charges, and all charges (e.g., Na⁺ and Cl−) have been superscripted consistently throughout the text.

Results

1. Figures of the protein-ligand docked complexes need to be shown. And the molecular docking results need to be discussed with more clarity.

1. Author response: We thank the reviewer for this valuable suggestion. In response, representative 2D interaction diagrams of the best multitarget docked complexes, along with the reference compounds, have been added to the revised manuscript (Figure 3). These figures illustrate the binding modes and key interactions established within the active sites of both NA and HMPV-F proteins. In addition, the molecular docking section has been revised to provide a clearer discussion of the docking results and ligand–protein interactions. The updated discussion now highlights the main interaction types involved in complex stabilization, including hydrogen bonding, hydrophobic contacts, π-interactions, and electrostatic interactions, as well as the key amino acid residues participating in ligand binding. Furthermore, the superior binding affinities of compounds such as E51 and M110 relative to the reference drugs are now discussed in greater detail to better support their potential multitarget antiviral activity.

2. The RMSD graphs appear to have been made using Microsoft Excel. The graphs need to be more refined and clearer. Kindly use Discovery Studio for better high-resolution and sharper images for the graphs.

2. Author response: We thank the reviewer for this helpful suggestion. The RMSD plots have been revised to improve clarity and overall visual presentation, including refined formatting and enhanced readability. The underlying RMSD data remain unchanged.

Attachment Submitted filename: Rebutal letter.docx

# Peer review: Decision Letter 1

**DOI:** 10.1371/journal.pone.0348517.r003

**Authors:** Lalit Samant

1 Jul 2026

Dear Dr. Chtita,

Thank you for submitting your manuscript to PLOS ONE. After careful consideration, we feel that it has merit but does not fully meet PLOS ONE’s publication criteria as it currently stands. Therefore, we invite you to submit a revised version of the manuscript that addresses the points raised during the review process.

Please submit your revised manuscript by Aug 15 2026 11:59PM. If you will need more time than this to complete your revisions, please reply to this message or contact the journal office at plosone@plos.org. When you're ready to submit your revision, log on to https://www.editorialmanager.com/pone/ and select the 'Submissions Needing Revision' folder to locate your manuscript file.

- A letter that responds to each point raised by the academic editor and reviewer(s). You should upload this letter as a separate file labeled 'Response to Reviewers'.
- A marked-up copy of your manuscript that highlights changes made to the original version. You should upload this as a separate file labeled 'Revised Manuscript with Track Changes'.
- An unmarked version of your revised paper without tracked changes. You should upload this as a separate file labeled 'Manuscript'.

If you would like to make changes to your financial disclosure, please include your updated statement in your cover letter. Guidelines for resubmitting your figure files are available below the reviewer comments at the end of this letter.

If applicable, we recommend that you deposit your laboratory protocols in protocols.io to enhance the reproducibility of your results. Protocols.io assigns your protocol its own identifier (DOI) so that it can be cited independently in the future. For instructions see: https://journals.plos.org/plosone/s/submission-guidelines#loc-laboratory-protocols. Additionally, PLOS ONE offers an option for publishing peer-reviewed Lab Protocol articles, which describe protocols hosted on protocols.io. Read more information on sharing protocols at https://plos.org/protocols?utm_medium=editorial-email&utm_source=authorletters&utm_campaign=protocols.

As the corresponding author, your ORCID iD is verified in the submission system and will appear in the published article. PLOS supports the use of ORCID, and we encourage all coauthors to register for an ORCID iD and use it as well. Please encourage your coauthors to verify their ORCID iD within the submission system before final acceptance, as unverified ORCID iDs will not appear in the published article. *Only*  the individual author can complete the verification step; PLOS staff *cannot*  verify ORCID iDs on behalf of authors.

We look forward to receiving your revised manuscript.

Kind regards,

Lalit Samant

Academic Editor

PLOS One

Journal Requirements:

If the reviewer comments include a recommendation to cite specific previously published works, please review and evaluate these publications to determine whether they are relevant and should be cited. There is no requirement to cite these works unless the editor has indicated otherwise.

Please review your reference list to ensure that it is complete and correct. If you have cited papers that have been retracted, please include the rationale for doing so in the manuscript text, or remove these references and replace them with relevant current references. Any changes to the reference list should be mentioned in the rebuttal letter that accompanies your revised manuscript. If you need to cite a retracted article, indicate the article’s retracted status in the References list and also include a citation and full reference for the retraction notice.

Additional Editor Comments:

Please check the suggestions mentioned and do the needful.

[Note: HTML markup is below. Please do not edit.]

Reviewers' comments:

Reviewer's Responses to Questions

**Comments to the Author**

Reviewer #1: All comments have been addressed

Reviewer #3: All comments have been addressed

**********

2. Is the manuscript technically sound, and do the data support the conclusions?

Reviewer #1: Yes

Reviewer #3: Yes

**********

3. Has the statistical analysis been performed appropriately and rigorously?

Reviewer #1: Yes

Reviewer #3: No

**********

4. Have the authors made all data underlying the findings in their manuscript fully available?

The PLOS Data policy

Reviewer #1: Yes

Reviewer #3: Yes

**********

5. Is the manuscript presented in an intelligible fashion and written in standard English?

Reviewer #1: Yes

Reviewer #3: Yes

**********

Reviewer #1: There is significant improvement in the revised manuscript. However, there still remains some stylistic and consistency issues that need to be addressed.

Reviewer #3: Major Comments:

1. The novelty of the study is not clearly established. The work primarily involves selecting a subset of phytoconstituents from an existing database followed by molecular docking, which does not sufficiently demonstrate innovation. Experimental validation through in vitro antiviral assays is necessary to support the proposed therapeutic potential of the identified compounds.

2. The claim that several phytochemicals outperform oseltamivir and ribavirin is based primarily on docking scores. Since docking energy alone does not reliably predict biological potency, additional binding free-energy calculations or experimental validation are needed.

3. The rationale for selecting the final lead compounds (M294 and C11) should be presented more systematically by integrating docking, ADMET, toxicity, and molecular dynamics results into a clear ranking or scoring framework.

4. The molecular dynamics analysis mainly reports RMSD, RMSF, and interaction profiles, but lacks complementary analyses such as MM-PBSA/MM-GBSA binding free-energy calculations.

5. The manuscript does not adequately discuss the limitations of using predicted ADMET and toxicity data, which should be interpreted cautiously.

6. Although two viral proteins were investigated, the manuscript provides limited discussion on the conservation of the selected binding sites across different influenza A and HMPV strains.

Minor Comments:

1. The manuscript contains several grammatical errors and awkward sentence constructions that require careful English language editing to improve readability.

2. The Introduction is longer than necessary and includes repeated discussion of previous studies, which could be condensed to improve focus on the objectives of the current work.

3. The docking interaction figures would benefit from clearer labeling of interacting residues and improved annotation of key hydrogen bonds, hydrophobic contacts, and electrostatic interactions.

**********

what does this mean?). If published, this will include your full peer review and any attached files.

If you choose “no”, your identity will remain anonymous but your review may still be made public.

**Do you want your identity to be public for this peer review?**  For information about this choice, including consent withdrawal, please see our Privacy Policy

Reviewer #1: No

Reviewer #3: No

**********

[NOTE: If reviewer comments were submitted as an attachment file, they will be attached to this email and accessible via the submission site. Please log into your account, locate the manuscript record, and check for the action link "View Attachments". If this link does not appear, there are no attachment files.]

To ensure your figures meet our technical requirements, please review our figure guidelines: https://journals.plos.org/plosone/s/figures

You may also use PLOS’s free figure tool, NAAS, to help you prepare publication quality figures: https://journals.plos.org/plosone/s/figures#loc-tools-for-figure-preparation.

NAAS will assess whether your figures meet our technical requirements by comparing each figure against our figure specifications.

Attachment Submitted filename: Comments and suggestions to the author.docx

# Author comment: Author response to Decision Letter 2

**DOI:** 10.1371/journal.pone.0348517.r004

19 Jul 2026

Response to the Reviewers

We sincerely thank the Editor and the Reviewers for their careful evaluation of our manuscript and for their valuable comments and constructive suggestions. We greatly appreciate the time and effort devoted to reviewing our work. Their insightful recommendations have helped us improve the quality, clarity, and scientific rigor of the manuscript. All comments have been carefully addressed, and the manuscript has been revised accordingly. The modifications introduced in the revised version are highlighted in yellow to facilitate their identification. A detailed, point-by-point response to each comment is provided below.

Comments and suggestions to the author

1. The abstract can be refined further.

Response: Thank you for your comment. The abstract has been revised to improve its clarity, conciseness, and overall readability while preserving the scientific content.

2. In the introduction, sentences like "Phytoconstituents are an in... these phytoconstituents" can be refined

Response: Thank you for your suggestion. The indicated sentences have been revised to improve clarity, readability, and overall language flow in the Introduction.

3. In section 2.1.2, “F currently lacks approved therapeutics…” needs to be revised. Although the second fusion protein has been identified as F, starting a sentence directly with F is not correct. Kindly consider changing to “furthermore, protein F…” or “Moreover, protein F…” for better readability.

Response: Thank you for your suggestion. The sentence has been revised by replacing “F” with “Moreover, protein F…” to improve readability and sentence structure.

4. In section 2.2.1, kindly check the format and font style of “10⁻⁴ units”. It appears inconsistent.

Response: Thank you for pointing this out. The formatting and font style of “10⁻⁴ units” have been corrected to ensure consistency throughout the manuscript.

5. In section 2.2.1, kindly maintain a consistent language style of either American English or British English as instructed by the journal guidelines. Also, perform a spell check.

“… geometry optimization and energy minimisation were performed in Avocadro using”

Response: Thank you for your comment. The manuscript has been thoroughly proofread, and the language has been revised to ensure consistent American English usage throughout, in accordance with the journal guidelines. Spelling and terminology have also been corrected where necessary.

6. In section 2.2.2, kindly change …” deduced using Discovery Studio 2021” to “…deduced using Discovery Studio, 2021

Response: Thank you for your suggestion. The sentence has been revised to read “deduced using Discovery Studio, 2021” as recommended.

Reviewer #3: Major Comments:

1. The novelty of the study is not clearly established. The work primarily involves selecting a subset of phytoconstituents from an existing database followed by molecular docking, which does not sufficiently demonstrate innovation. Experimental validation through in vitro antiviral assays is necessary to support the proposed therapeutic potential of the identified compounds.

Response: We sincerely thank the reviewer for this constructive comment. We agree that the novelty of the study should be stated more explicitly. Accordingly, the Introduction and Section 2.1.2 (Biological Target) were revised to better emphasize the innovative aspects of our study, and the Conclusion was updated to further clarify the originality of the proposed computational strategy and the scope of the findings (highlighted in yellow in the revised manuscript).

The novelty of this study does not reside in the use of previously reported phytoconstituents alone, but rather in the integrated computational strategy applied to identify broad-spectrum antiviral candidates. Specifically, our work simultaneously investigates two distinct respiratory viruses, influenza A virus and human metapneumovirus (HMPV), by targeting two key viral proteins involved in different stages of the viral life cycle, namely neuraminidase (NA) and the fusion protein (F). To the best of our knowledge, computational studies combining these two unrelated viral targets within a single screening workflow remain limited.

In addition, our study extends beyond conventional molecular docking by integrating successive filtering through drug-likeness assessment, comprehensive ADMET and toxicity prediction, followed by molecular dynamics simulations and MM-PBSA binding free-energy calculations to evaluate the stability of the selected protein–ligand complexes. This sequential workflow enabled the rational prioritization of the most promising candidates rather than relying solely on docking scores.

Regarding the request for experimental validation, we fully agree that in vitro antiviral assays are essential to confirm the computational predictions. However, the present work was designed as an in silico screening study aimed at identifying and prioritizing promising lead compounds for future experimental investigations. We have therefore revised the Conclusion to emphasize that the proposed compounds should be considered computational lead candidates requiring subsequent experimental validation, particularly through in vitro antiviral assays, followed by in vivo investigations, before any therapeutic conclusions can be drawn.

2. The claim that several phytochemicals outperform oseltamivir and ribavirin is based primarily on docking scores. Since docking energy alone does not reliably predict biological potency, additional binding free-energy calculations or experimental validation are needed.

Response: We sincerely thank the reviewer for this valuable comment. We agree that molecular docking scores alone are not sufficient to predict biological potency and should not be interpreted as evidence of superior antiviral activity. Accordingly, we have revised the manuscript to avoid overinterpretation of the docking results. Throughout the manuscript (Abstract, Results, and Conclusion), statements suggesting that the selected phytoconstituents "outperformed" the reference drugs have been replaced with more appropriate wording indicating more favorable predicted binding energies.

In response to the reviewer's recommendation, we performed MM-PBSA binding free-energy calculations as an additional validation step. The corresponding methodology and results have been incorporated into the revised manuscript (Sections 2.5 and 3.3.4, Table 7). The MM-PBSA analysis provided an independent energetic assessment of the selected protein–ligand complexes and further supported the molecular docking and molecular dynamics findings, reinforcing the prioritization of M294 and C11 as the most promising broad-spectrum antiviral lead compounds.

Furthermore, we clarified that the prioritization of the selected compounds was not based solely on docking scores but on an integrated computational workflow combining molecular docking, drug-likeness evaluation, ADMET and toxicity profiling, molecular dynamics simulations, and MM-PBSA binding free-energy calculations to improve the reliability of candidate selection.

Regarding the reviewer's recommendation for experimental validation, we fully agree that in vitro and in vivo studies are essential to confirm the computational predictions. However, the present study was designed as an in silico screening study for lead identification. Therefore, we have revised the Conclusion to explicitly state that the identified compounds should be considered computational lead candidates whose predicted antiviral potential requires experimental validation, particularly through in vitro antiviral assays followed by in vivo investigations.

3. The rationale for selecting the final lead compounds (M294 and C11) should be presented more systematically by integrating docking, ADMET, toxicity, and molecular dynamics results into a clear ranking or scoring framework.

Response: We sincerely thank the reviewer for this valuable suggestion. We agree that the rationale for selecting the final lead compounds should be presented more systematically. Accordingly, we have revised the manuscript to clarify the sequential prioritization strategy adopted throughout the study.

Specifically, we have added a summary table entitled "Sequential prioritization framework for the selection of the final broad-spectrum antiviral lead compounds (Table 8. )", which integrates the successive selection criteria, including molecular docking, drug-likeness evaluation, ADMET and toxicity profiling, molecular dynamics analyses and MM-PBSA binding free-energy calculations. This framework clearly illustrates how the initial library of phytoconstituents was progressively narrowed down to the final lead compounds.

In addition, the end of the Molecular Dynamics section has been revised to explicitly explain that M294 and C11 were prioritized because they consistently fulfilled all selection criteria by combining favorable multi-target docking performance, acceptable predicted pharmacokinetic and toxicological profiles, and the most stable interactions with both viral targets during the molecular dynamics simulations.

4. The molecular dynamics analysis mainly reports RMSD, RMSF, and interaction profiles, but lacks complementary analyses such as MM-PBSA/MM-GBSA binding free-energy calculations.

Response: Thank you for this valuable suggestion. In response to the reviewer's comment, we performed MM-PBSA binding free-energy calculations as a complementary analysis to the molecular dynamics simulations. The MM-PBSA results have been incorporated into the revised manuscript and provide a quantitative estimation of the binding free energies of the selected protein–ligand complexes. These calculations further support the stability and binding affinity observed from the RMSD, RMSF, and protein–ligand interaction analyses, reinforcing the prioritization of M294 and C11 as the most promising broad-spectrum antiviral candidates. The corresponding methodology, results, and discussion have been added to the revised manuscript (Section 3.3.4 , Table 7).

5. The manuscript does not adequately discuss the limitations of using predicted ADMET and toxicity data, which should be interpreted cautiously.

Response: We sincerely thank the reviewer for this valuable comment. We agree that computational ADMET and toxicity predictions should be interpreted with appropriate caution, as they are based on predictive models and cannot fully reflect the complexity of in vivo pharmacokinetic and toxicological behavior.

Accordingly, we have revised Section 3.2.2 (Evaluation of pharmacokinetics and toxicity properties) to explicitly acknowledge the limitations of these computational predictions. The revised manuscript has been updated to clarify that the predicted ADMET and toxicity profiles provide preliminary estimates that are valuable for early-stage compound prioritization but cannot replace experimental pharmacokinetic and toxicological evaluations. We also emphasize that the selected compounds should be considered exploratory in silico lead candidates requiring further experimental validation before any definitive conclusions regarding their safety or therapeutic potential can be drawn.

6. Although two viral proteins were investigated, the manuscript provides limited discussion on the conservation of the selected binding sites across different influenza A and HMPV strains.

Response: We sincerely thank the reviewer for this valuable comment. We agree that the conservation of the selected binding sites across different influenza A and HMPV strains deserved further clarification. Accordingly, we have revised Section 2.1.2 (Biological Target) to better justify the selection of the two crystallographic structures used in this study. Specifically, we added a discussion highlighting that the catalytic site of influenza A neuraminidase is highly conserved among the major influenza A subtypes, whereas the HMPV fusion (F) protein contains conserved functional regions across the principal HMPV genotypes. These characteristics support the use of PDB structures 2HT7 and 7SEJ as biologically relevant models for the computational identification of potential broad-spectrum antiviral candidates. We believe that this addition clarifies the rationale for target selection and strengthens the biological relevance of our computational approach.

Minor Comments:

1. The manuscript contains several grammatical errors and awkward sentence constructions that require careful English language editing to improve readability.

Response: We sincerely thank the reviewer for this valuable comment. The entire manuscript has been carefully revised to improve the quality of the English language, sentence structure, and overall readability. Grammatical errors, awkward sentence constructions, and stylistic inconsistencies were corrected throughout the text. In addition, the manuscript was thoroughly edited to ensure consistent use of American English spelling and terminology, thereby improving its clarity and readability.

2. The Introduction is longer than necessary and includes repeated discussion of previous studies, which could be condensed to improve focus on the objectives of the current work.

Response: We sincerely thank the reviewer for this constructive suggestion. The Introduction has been revised to improve its conciseness and focus. Repetitive background information, particularly regarding the clinical presentation of respiratory viral infections and the discussion of our previous studies, has been condensed or removed. The revised Introduction now places greater emphasis on the scientific rationale, the novelty of the proposed multi-target computational strategy, and the objectives of the present study.

3. The docking interaction figures would benefit from clearer labeling of interacting residues and improved annotation of key hydrogen bonds, hydrophobic contacts, and electrostatic interactions.

Response: We sincerely thank the reviewer for this helpful suggestion. The molecular docking interaction figures have been revised to improve their clarity and readability. The labeling of interacting amino acid residues has been enhanced, and the key hydrogen bonds, hydrophobic interactions, and electrostatic interactions have been more clearly annotated. These improvements facilitate the interpretation of the protein–ligand binding modes and highlight the principal interactions responsible for ligand stabilization within the binding sites.

Attachment Submitted filename: Comments_and_suggestions_to_the_author_auresp_2.docx

# Editor report: Decision Letter 2

**DOI:** 10.1371/journal.pone.0348517.r005

**Authors:** Lalit Samant

3 Aug 2026

Dear Dr. Chtita,

Thank you for submitting your manuscript to PLOS ONE. After careful consideration, we feel that it has merit but does not fully meet PLOS ONE’s publication criteria as it currently stands. Therefore, we invite you to submit a revised version of the manuscript that addresses the points raised during the review process.

Please submit your revised manuscript by Sep 17 2026 11:59PM. If you will need more time than this to complete your revisions, please reply to this message or contact the journal office at plosone@plos.org. When you're ready to submit your revision, log on to https://www.editorialmanager.com/pone/ and select the 'Submissions Needing Revision' folder to locate your manuscript file.

- A letter that responds to each point raised by the academic editor and reviewer(s). You should upload this letter as a separate file labeled 'Response to Reviewers'.
- A marked-up copy of your manuscript that highlights changes made to the original version. You should upload this as a separate file labeled 'Revised Manuscript with Track Changes'.
- An unmarked version of your revised paper without tracked changes. You should upload this as a separate file labeled 'Manuscript'.

If you would like to make changes to your financial disclosure, please include your updated statement in your cover letter. Guidelines for resubmitting your figure files are available below the reviewer comments at the end of this letter.

If applicable, we recommend that you deposit your laboratory protocols in protocols.io to enhance the reproducibility of your results. Protocols.io assigns your protocol its own identifier (DOI) so that it can be cited independently in the future. For instructions see: https://journals.plos.org/plosone/s/submission-guidelines#loc-laboratory-protocols. Additionally, PLOS ONE offers an option for publishing peer-reviewed Lab Protocol articles, which describe protocols hosted on protocols.io. Read more information on sharing protocols at https://plos.org/protocols?utm_medium=editorial-email&utm_source=authorletters&utm_campaign=protocols.

As the corresponding author, your ORCID iD is verified in the submission system and will appear in the published article. PLOS supports the use of ORCID, and we encourage all coauthors to register for an ORCID iD and use it as well. Please encourage your coauthors to verify their ORCID iD within the submission system before final acceptance, as unverified ORCID iDs will not appear in the published article. *Only*  the individual author can complete the verification step; PLOS staff *cannot*  verify ORCID iDs on behalf of authors.

We look forward to receiving your revised manuscript.

Kind regards,

Lalit Samant

Academic Editor

PLOS One

Journal Requirements:

If the reviewer comments include a recommendation to cite specific previously published works, please review and evaluate these publications to determine whether they are relevant and should be cited. There is no requirement to cite these works unless the editor has indicated otherwise.

Please review your reference list to ensure that it is complete and correct. If you have cited papers that have been retracted, please include the rationale for doing so in the manuscript text, or remove these references and replace them with relevant current references. Any changes to the reference list should be mentioned in the rebuttal letter that accompanies your revised manuscript. If you need to cite a retracted article, indicate the article’s retracted status in the References list and also include a citation and full reference for the retraction notice.

[Note: HTML markup is below. Please do not edit.]

[NOTE: If reviewer comments were submitted as an attachment file, they will be attached to this email and accessible via the submission site. Please log into your account, locate the manuscript record, and check for the action link "View Attachments". If this link does not appear, there are no attachment files.]

To ensure your figures meet our technical requirements, please review our figure guidelines: https://journals.plos.org/plosone/s/figures

You may also use PLOS’s free figure tool, NAAS, to help you prepare publication quality figures: https://journals.plos.org/plosone/s/figures#loc-tools-for-figure-preparation.

NAAS will assess whether your figures meet our technical requirements by comparing each figure against our figure specifications.

Attachment Submitted filename: Comments and suggestions to the author.docx

# Author comment: Author response to Decision Letter 3

**DOI:** 10.1371/journal.pone.0348517.r006

5 Aug 2026

Dear Editor

We would like to emphasize that these comments have already been addressed in our previous round of revisions. We have carefully responded to each point raised by the reviewer and incorporated the corresponding modifications into the revised manuscript. For the Editor's and Reviewer's convenience, we provide our responses below once again.

Best regards

Response to the Reviewers

We sincerely thank the Editor and the Reviewers for their careful evaluation of our manuscript and for their valuable comments and constructive suggestions. We greatly appreciate the time and effort devoted to reviewing our work. Their insightful recommendations have helped us improve the quality, clarity, and scientific rigor of the manuscript. All comments have been carefully addressed, and the manuscript has been revised accordingly. The modifications introduced in the revised version are highlighted in yellow to facilitate their identification. A detailed, point-by-point response to each comment is provided below.

Comments and suggestions to the author

1. The abstract can be refined further.

Response: Thank you for your comment. The abstract has been revised to improve its clarity, conciseness, and overall readability while preserving the scientific content.

2. In the introduction, sentences like "Phytoconstituents are an in... these phytoconstituents" can be refined

Response: Thank you for your suggestion. The indicated sentences have been revised to improve clarity, readability, and overall language flow in the Introduction.

3. In section 2.1.2, “F currently lacks approved therapeutics…” needs to be revised. Although the second fusion protein has been identified as F, starting a sentence directly with F is not correct. Kindly consider changing to “furthermore, protein F…” or “Moreover, protein F…” for better readability.

Response: Thank you for your suggestion. The sentence has been revised by replacing “F” with “Moreover, protein F…” to improve readability and sentence structure.

4. In section 2.2.1, kindly check the format and font style of “10⁻⁴ units”. It appears inconsistent.

Response: Thank you for pointing this out. The formatting and font style of “10⁻⁴ units” have been corrected to ensure consistency throughout the manuscript.

5. In section 2.2.1, kindly maintain a consistent language style of either American English or British English as instructed by the journal guidelines. Also, perform a spell check.

“… geometry optimization and energy minimisation were performed in Avocadro using”

Response: Thank you for your comment. The manuscript has been thoroughly proofread, and the language has been revised to ensure consistent American English usage throughout, in accordance with the journal guidelines. Spelling and terminology have also been corrected where necessary.

6. In section 2.2.2, kindly change …” deduced using Discovery Studio 2021” to “…deduced using Discovery Studio, 2021

Response: Thank you for your suggestion. The sentence has been revised to read “deduced using Discovery Studio, 2021” as recommended.

Attachment Submitted filename: Responses to reviewers.docx

# Editor report: Decision Letter 3

**DOI:** 10.1371/journal.pone.0348517.r007

**Authors:** Lalit Samant

12 Aug 2026

Repositioning antiviral phytoconstituents as broad-spectrum inhibitors of influenza A neuraminidase and human metapneumovirus fusion protein

PONE-D-26-18338R3

Dear Dr. Chtita,

We’re pleased to inform you that your manuscript has been judged scientifically suitable for publication and will be formally accepted for publication once it meets all outstanding technical requirements.

Within one week, you’ll receive an e-mail detailing the required amendments. When these have been addressed, you’ll receive a formal acceptance letter and your manuscript will be scheduled for publication.

An invoice will be generated when your article is formally accepted. Please note, if your institution has a publishing partnership with PLOS and your article meets the relevant criteria, all or part of your publication costs will be covered. Please make sure your user information is up-to-date by logging into Editorial Manager at Editorial Manager® and clicking the ‘Update My Information' link at the top of the page. For questions related to billing, please contact billing support.

If your institution or institutions have a press office, please notify them about your upcoming paper to help maximize its impact. If they’ll be preparing press materials, please inform our press team as soon as possible -- no later than 48 hours after receiving the formal acceptance. Your manuscript will remain under strict press embargo until 2 pm Eastern Time on the date of publication. For more information, please contact onepress@plos.org.

Kind regards,

Lalit Samant

Academic Editor

PLOS One

Additional Editor Comments (optional):

Reviewers' comments:

# Editor report: Acceptance letter

**DOI:** 10.1371/journal.pone.0348517.r008

**Authors:** Lalit Samant

PONE-D-26-18338R3

PLOS One

Dear Dr. Chtita,

I'm pleased to inform you that your manuscript has been deemed suitable for publication in PLOS One. Congratulations! Your manuscript is now being handed over to our production team.

At this stage, our production department will prepare your paper for publication. This includes ensuring the following:

* All references, tables, and figures are properly cited

* All relevant supporting information is included in the manuscript submission,

* There are no issues that prevent the paper from being properly typeset

You will receive further instructions from the production team, including instructions on how to review your proof when it is ready. Please keep in mind that we are working through a large volume of accepted articles, so please give us a few days to review your paper and let you know the next and final steps.

Lastly, if your institution or institutions have a press office, please let them know about your upcoming paper now to help maximize its impact. If they'll be preparing press materials, please inform our press team within the next 48 hours. Your manuscript will remain under strict press embargo until 2 pm Eastern Time on the date of publication. For more information, please contact onepress@plos.org.

You will receive an invoice from PLOS for your publication fee after your manuscript has reached the completed accept phase. If you receive an email requesting payment before acceptance or for any other service, this may be a phishing scheme. Learn how to identify phishing emails and protect your accounts at https://explore.plos.org/phishing.

If we can help with anything else, please email us at customercare@plos.org.

Thank you for submitting your work to PLOS One and supporting open access.

Kind regards,

PLOS ONE Editorial Office Staff

on behalf of

Dr. Lalit Samant

Academic Editor

PLOS One
